# Microarray Analysis of Gene Expression in *Saccharomyces cerevisiae kap108*Δ Mutants upon Addition of Oxidative Stress

**DOI:** 10.1534/g3.116.027011

**Published:** 2016-02-17

**Authors:** Kenneth D. Belanger, Nathaniel Larson, Jonathan Kahn, Dmitry Tkachev, Ahmet Ay

**Affiliations:** * Department of Biology, Colgate University, Hamilton, New York 13346; † Department of Mathematics, Colgate University, Hamilton, New York 13346

**Keywords:** karyopherin, importin, nuclear transport, gene expression, oxidative stress

## Abstract

Protein transport between the nucleus and cytoplasm of eukaryotic cells is tightly regulated, providing a mechanism for controlling intracellular localization of proteins, and regulating gene expression. In this study, we have investigated the importance of nucleocytoplasmic transport mediated by the karyopherin Kap108 in regulating cellular responses to oxidative stress in *Saccharomyces cerevisiae*. We carried out microarray analyses on wild-type and *kap108* mutant cells grown under normal conditions, shortly after introduction of oxidative stress, after 1 hr of oxidative stress, and 1 hr after oxidative stress was removed. We observe more than 500 genes that undergo a 40% or greater change in differential expression between wild-type and *kap108Δ* cells under at least one of these conditions. Genes undergoing changes in expression can be categorized in two general groups: 1) those that are differentially expressed between wild-type and *kap108*Δ cells, no matter the oxidative stress conditions; and 2) those that have patterns of response dependent upon both the absence of Kap108, and introduction or removal of oxidative stress. Gene ontology analysis reveals that, among the genes whose expression is reduced in the absence of Kap108 are those involved in stress response and intracellular transport, while those overexpressed are largely involved in mating and pheromone response. We also identified 25 clusters of genes that undergo similar patterns of change in gene expression when oxidative stresses are added and subsequently removed, including genes involved in stress response, oxidation–reduction processing, iron homeostasis, ascospore wall assembly, transmembrane transport, and cell fusion during mating. These data suggest that Kap108 is important for regulating expression of genes involved in a variety of specific cell functions.

The regulated transport of proteins between the cytoplasm and the nucleus of eukaryotic cells provides an important mechanism for controlling gene expression. Since protein synthesis occurs in the cytoplasm, and the genetic material resides within the nucleus, proteins responsible for regulating gene expression must enter the nucleus to activate or repress gene activity. Transcription factors active within the nucleus can subsequently have their control of transcription modulated by regulated shuttling back out of the nucleus. This regulated nucleocytoplasmic shuttling into, and out of, the nucleus occurs specifically through large, macromolecular assemblages termed nuclear pore complexes (NPCs). Each NPC is comprised of more than 30 different proteins, termed nucleoporins (Nups), which assemble in multiples of eight to form a complex that includes nearly 500 total polypeptides (reviewed in Wente and Rout 2010; Hoelz *et al.* 2011). NPCs perforate both phospholipid bilayers of the nuclear envelope, forming a nuclear pore through which proteins, RNAs, and other molecules can pass. These NPCs are the conduit for the vast majority of molecular transit between the cytoplasm and nucleus in eukaryotes (see Kabachinski and Schwartz 2015).

The regulated passage of most proteins through the NPCs is mediated by soluble transport factors referred to as karyopherins (Kaps) (Quan *et al.* 2008; Kimura and Imamoto 2014). Kaps that transport cargo proteins into the nucleus are referred to as “importins,” and those that export specific substrates from the nucleus are “exportins” (for reviews, see Wente and Rout 2010; Bauer *et al.* 2015). Importins function by associating with a nuclear localization signal (NLS) on the cargo protein to be imported, accompanying the NLS-containing cargo to the NPCs, and mediating translocation of the Kap/NLS-cargo complex through the nuclear pore. Once inside the nucleus, the Kap and cargo disassemble upon interaction with the GTP-bound form of the small G-protein Ran (Fried and Kutay 2003). Exportins bind to a nuclear export signal (NES) on their cargo in a Ran-GTP-dependent manner, are exported as an exportin/cargo/Ran-GTP heterotrimer, and dissociate from cargo upon GTP hydrolysis in the cytoplasm (see Terry *et al.* 2007; Cook and Conti 2010; Bauer *et al.* 2015).

Yeast cells encode 14 distinct karyopherin proteins, at least 10 of which function as importins (Quan *et al.* 2008; Kimura and Imamoto 2014). For some of the Kaps, the proteins they recognize, and the cargoes they transport are well characterized (Lange *et al.* 2007; Kimura and Imamoto 2014). But the cargoes and function of other karyopherins are much less well defined. An example is the yeast importin Kap108/Sxm1, for which only two cargoes have been defined: the Pab1 poly(A)-binding protein (Brune *et al.* 2005), and the Lhp1 tRNA maturation factor (Rosenblum *et al.* 1997). The mammalian karyopherin with which Kap108 has greatest sequence similarity is the human Importin-8, for which no NLS has been isolated, and only a few potential transport substrates have been identified (Dean *et al.* 2001; Yao *et al.* 2008; Weinmann *et al.* 2009).

Some intracellular activities are regulated, at least in part, by the controlled nucleocytoplasmic shuttling of specific polypeptides. Cellular responses to oxidative stresses, both in the form of superoxide radicals and other chemical oxidative agents, are also dependent, at least in part, on the nucleocytoplasmic shuttling of proteins (Yan *et al.* 1998; Kuge *et al.* 2001; Görner *et al.* 2002; Crampton *et al.* 2009; Gulshan *et al.* 2012). Introduction of an oxidative stress to the yeast *Saccharomyces cerevisiae* results in changes in the expression—either upward or downward—of nearly 2000 genes (Causton *et al.* 2001; Gasch *et al.* 2000). A great many of these genes are controlled by two distinct transcription factors, Yap1 and Msn2-Msn4, each of which regulate increases and decreases in transcription of distinct, but overlapping, sets of genes in response to oxidative stress and other stressors (Toone and Jones 1999; Gasch *et al.* 2000; Causton *et al.* 2001; Moye-Rowley 2002; Hasan *et al.* 2002; Hao *et al.* 2013). Both of these transcription factors are located primarily in the cytoplasm during log growth in yeast, and undergo rapid nuclear import and accumulation upon introduction of an oxidative stress. Yap1 and Msn2-Msn4 are both imported by the karyopherins Kap121 and Kap123 (Isoyama *et al.* 2001; De Wever *et al.* 2005; Garmendia-Torres *et al.* 2007). Yap1 undergoes regulated export by Crm1/Xpo1 (Kuge *et al.* 1998; Yan *et al.* 1998), while Msn2-Msn4 is exported by Msn5/Kap142 in response to oxidative stress (Görner *et al.* 2002). However, Yap1 and Msn2/Msn4 are not the sole regulators of gene expression changes in response to oxidative stress in yeast (Winzeler *et al.* 1999; Gasch *et al.* 2000; Causton *et al.* 2001; Moye-Rowley 2002), and it is likely that nucleocytoplasmic regulation of other transcription factors impacts stress response.

In order to identify genes whose expression is influenced by the importin Kap108, we performed microarray analyses on wild-type and *kap108*Δ cells grown under normal conditions. We observed that over 70 genes underwent differential expression, suggesting that Kap108 likely mediates the nuclear import of some transcription factors, and/or other transcriptional regulators. Genes constitutively upregulated in the absence of Kap108 include those involved in mating and pheromone response. Genes whose expression is reduced in the absence of Kap108 are associated with cellular transport and stress response. Analysis of gene expression upon addition, and subsequent removal, of oxidative stress revealed more than 500 genes that undergo change to differential expression between wild-type and *kap108*Δ yeast, with enrichment for genes involved in stress response, oxidation–reduction process, iron ion homeostasis, ascospore wall assembly, transmembrane transport, and cytogamy. Taken together, these data indicate that Kap108-mediated nucleocytoplasmic transport is likely important for expression of genes involved in multiple cellular functions.

## Materials and Methods

### Microarray analysis

Yeast strains BY4742 and KBY1357 (BY4742 *kap108*Δ) were obtained from OpenBiosystems (Lafayette, CO). Media recipes and cell culture conditions are described in Adams *et al.* (1997). KBY1357 and BY4742 cells were grown to A_600_ = 0.3 in 1 L YPD; 200 ml of culture was removed, spun at 3000 rpm for 10 min, and frozen at –80°. Fresh H_2_O_2_ was added to the remaining culture to a 1.0 mM final concentration, and each culture incubated for 10 min at 30°, after which 200 ml of culture was removed, spun, and frozen as above. This was repeated with another 200 ml of cells, 1 hr after H_2_O_2_ addition. The remaining culture was then pelleted, YPD + H_2_O_2_ media removed, cells resuspended in 250 ml YPD, and culture incubated for 1 hr at 30°. Cells were then pelleted and frozen as before. RNA from each pellet was isolated using the RiboPure-Yeast RNA isolation kit (Ambion, Inc.) as per the manufacturer’s instructions. RNA probes were labeled and hybridized to Agilent Yeast 4x44K microarray kit by MoGene, LC (St. Louis, MO). RNA isolation for each condition was done in triplicate, and analysis was done with dye reversal.

The Agilent spectrometer used by MoGene applied a linear loess dye normalization to the two channels on each array. Replicate probes on each array were summarized by taking their geometric mean. Once replicate probes had been summarized, further background correction, and data normalization took place using GeneSpring GX 13.0, which involved a threshold step that set all probes with intensity values of less than one up to one, a log_2_ transformation, and a percentile shift to the 75th percentile.

Data from cells on the preoxidation arrays were analyzed using an unpaired *t*-test. Genes that underwent a 1.5-fold change with *P <* 0.05 (raw) were considered to have significant differential expression. Gene ontology (GO) analysis of these data took place using YEASTRACT for both overexpressed and underexpressed genes (Teixeira *et al.* 2006; 2014; Monteiro *et al.* 2008; Abdulrehman *et al.* 2011).

Differential expression values were then converted back from their log_2_ transformation, and the effect of addition of oxidative stress was determined by filtering all genes with over a 40% change in at least one of the samples subjected to oxidative stress as compared to the preoxidation samples. This cutoff was chosen to provide both stringency and a sizable gene list. These filtered genes were then clustered according to patterns of change in differential expression. This was done by considering three possible changes to differential expression that could occur between time points: an increase, a decrease, or no change. To qualify as increasing or decreasing, differential expression had to change by at least 20%. The 20% cutoff was chosen because it is less than 40%, but still stringent enough to effectively cluster genes. Ultimately, this gives 25 groups of genes that are representative of 25 different possible patterns of change in differential gene expression. GO analysis was performed for each group using YEASTRACT, which also calculated *P*-values using a hypergeometric distribution with a Bonferroni correction (Teixeira *et al.* 2006, 2014; Monteiro *et al.* 2008; Abdulrehman *et al.* 2011).

### Data availability

The data discussed in this publication have been deposited in NCBI’s Gene Expression Omnibus (Edgar *et al.* 2002), and are accessible through GEO Series accession number GSE71068 (http://www.ncbi.nlm.nih.gov/geo/query/acc.cgi?acc=GSE71068). Strains are available upon request. Supplemental Material, Figure S1 contains YEASTRACT GO analysis of underexpressed genes in mutant cells under normal growth conditions. Figure S2 contains YEASTRACT GO analysis of overexpressed genes in mutant cells under normal growth conditions. Figure S3 contains YEASTRACT GO analysis of genes filtered based on having at least a 40% change to differential gene expression in at least one of three postoxidation time points as compared to normal growth conditions. Figure S4 contains all 25 clusters, including the genes that comprise them, graphical depictions of changes to expression, and links to YEASTRACT GO analyses performed for each one. Figure S5 contains all custom code used in this study, which was implemented in R.

## Results

The mammalian importin-8 protein is essential for normal intracellular localization of the Smad4 protein that functions as a component of the TGF-β transcription factor (Yao *et al.* 2008), and for nuclear transport and proper function of the Ago2 component of the miRNA-processing argonaute complex (Weinmann *et al.* 2009). Kap108, the yeast importin with greatest similarity to importin-8, also has had few transport targets identified (Rosenblum *et al.* 1997; Brune *et al.* 2005), but those targets are involved, at least indirectly, in gene expression. To identify potential transport substrates for Kap108, or downstream targets of those substrates, we performed microarray analyses comparing genome-wide expression patterns in wild-type cells, and cells lacking Kap108. We examined changes in gene expression between wild-type and *kap108*Δ cells under normal growth conditions, 10 min after introduction of 1 mM H_2_O_2_, 1 hr after addition of H_2_O_2_, and 1 hr after the oxidative stress was removed.

**Figure 1:**
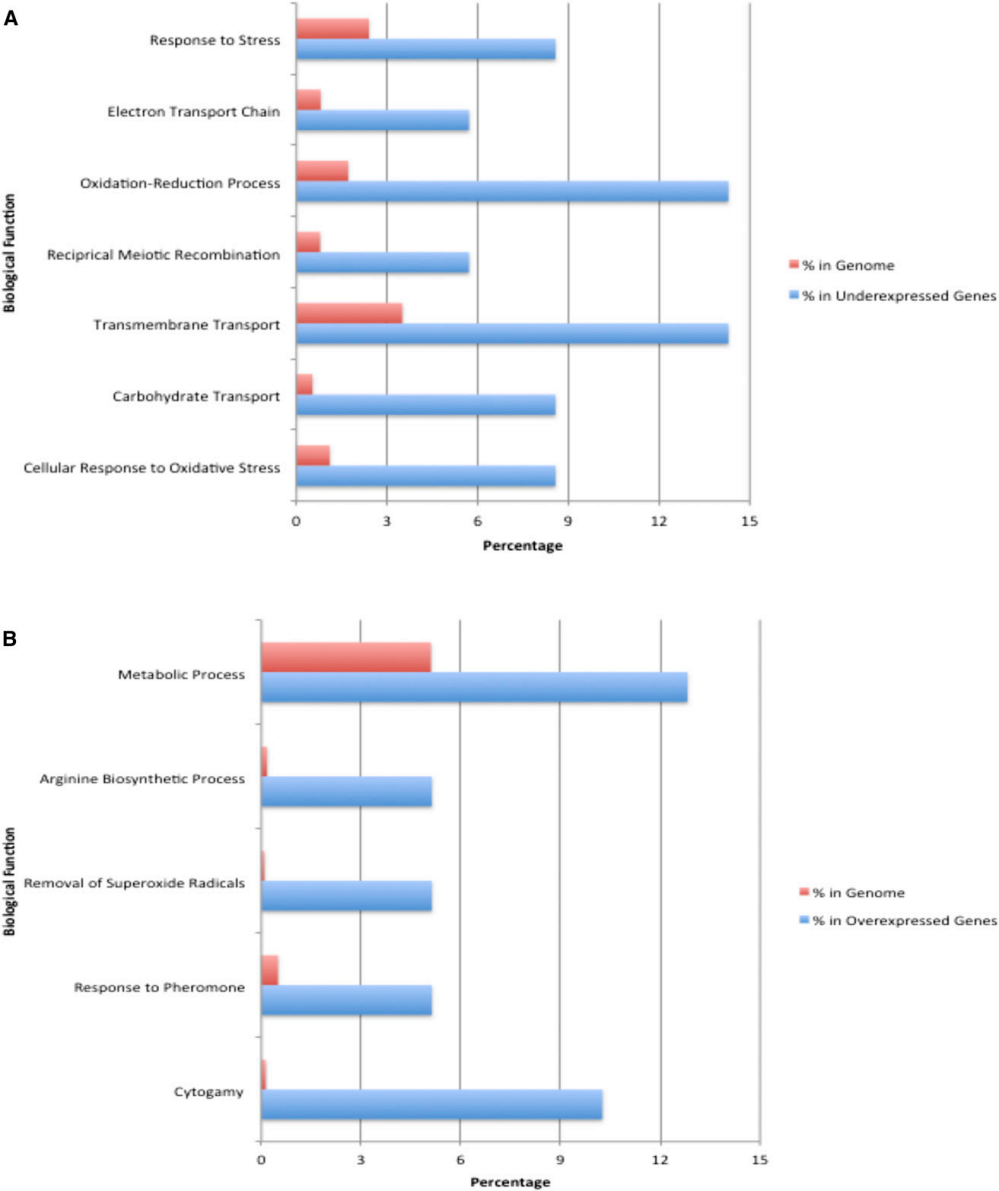
Genes involved in seven biological functions have decreased expression in *kap108∆* cells, while genes for five functions are overexpressed. Wild-type and *kap108∆* yeast were grown in YPD media for 72 hr at 30°, and normalized Agilent two-channel microarray gene expression data from three replicate arrays of these cultures were filtered for genes that experience at least a 1.5-fold change in differential expression with *P <* 0.05 as determined by an unpaired *t*-test. YEASTRACT was used to perform gene ontology (GO) analysis on the (A) 35 genes with reduced expression, and (B) 39 overexpressed genes, and only groups with significant differences from representation in the yeast genome (*P <* 0.01) are shown. “Percent in genome” refers to the percentage of genes in the entire yeast genome that have the given biological function. “Percent in underexpressed genes” refers to what percentage of genes significantly underexpressed by at least 1.5-fold have the given biological function (A). “Percent in overexpressed genes” refers to what percentage of genes significantly overexpressed by at least 1.5-fold have the given biological function (B).

**Figure 2:**
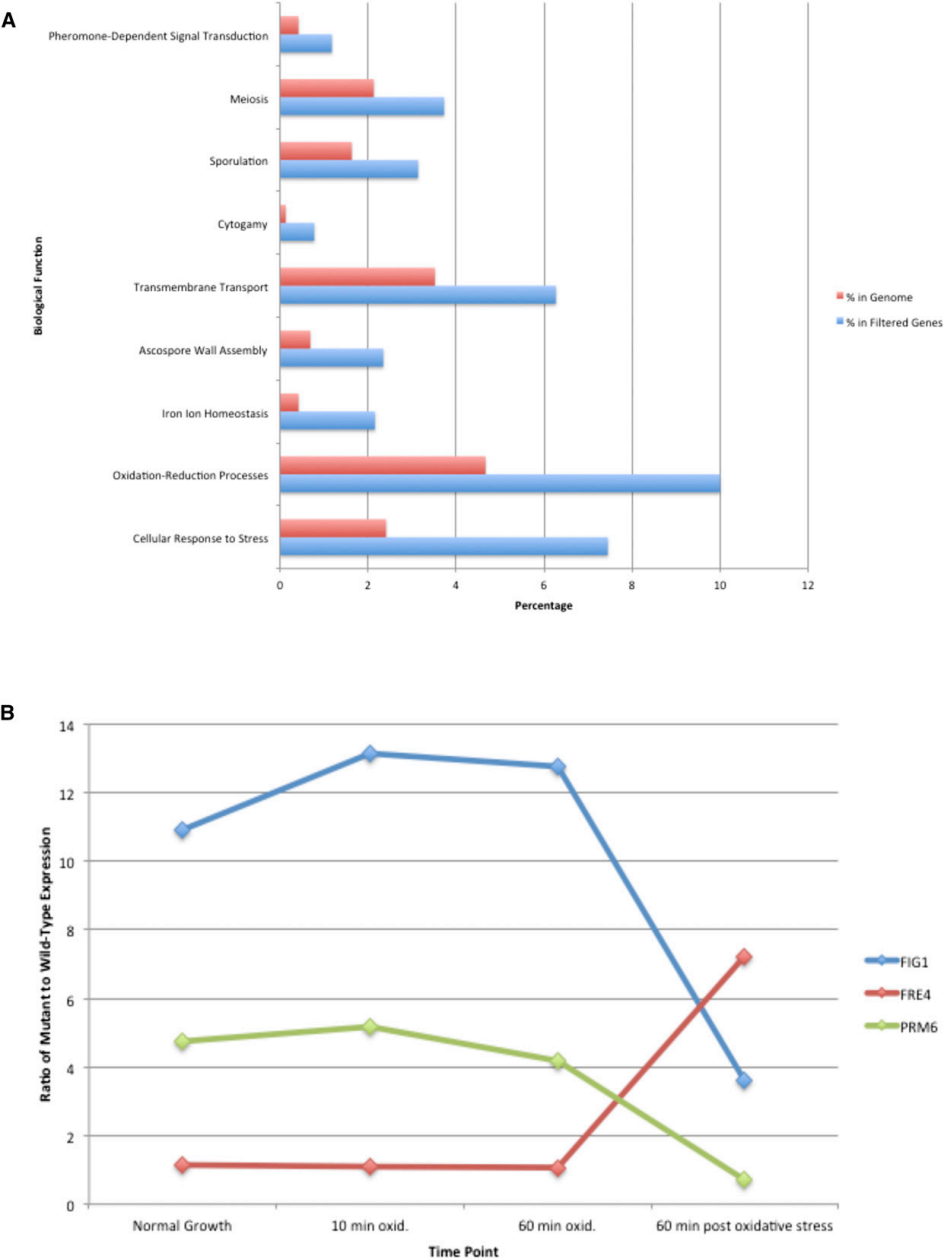
Nine biological functions are overrepresented among genes that undergo significant changes in differential expression between wild-type and *kap108∆* yeast cells after the addition of oxidative stress. Wild-type and *kap108∆* yeast cultures were grown at 30° for 72 hr on YPD (normal growth). At *t* = 0, H_2_O_2_ was added, and yeast cells were removed after 10 min (10 min oxid), and 60 min (60 min oxid). Cells were then rinsed, resuspended in YPD, and incubated for 60 min in the absence of H_2_O_2_ (60 min postoxidative stress). (A) Normalized Agilent two-channel microarray gene expression data for three replicates of each of these four conditions were filtered for genes that undergo at least a 40% change in differential expression between preoxidation and any of the other three time points. YEASTRACT was used to perform a GO analysis of the 511 resulting genes. “Percent in genome” refers to the percentage of genes in the genome with the given biological function, while “percent in filtered genes” refers to the percentage of genes with the given biological function in the filtered gene list. (B) Filtered genes were clustered based on three possible changes to differential expression between time points: an increase of at least 20% (“Up”), a decrease of at least 20% (“Dn”), or no change (“Nc”). All 27 possible patterns were considered. Plotted are the mutant to wild-type expression ratios at all four time points for three noteworthy example genes: FIG1, which clusters in UpNcDn, *FRE4*, which is NcNcUp, and *PRM6*, which is NcNcDn.

Two channel microarray data for gene expression from wild-type and *kap108∆* cells were examined to determine which genes are under and overexpressed in the absence of the Kap108 importin. Under normal conditions, 74 genes have statistically significant, 1.5-fold, or greater, differences in gene expression between wild-type and *kap108∆* cells, as determined by an unpaired *t*-test (*P <* 0.05, see Table S1). Of these, 35 are underexpressed, including *GPX2*, *TRX2*, and *TSA1*, which are associated with response to oxidative stress (*P <* 0.001). These genes are representative of 4.35% of all oxidative stress response genes in the yeast genome according to GO analysis done using YEASTRACT (Teixeira *et al.* 2006, 2014; Monteiro *et al.* 2008; Abdulrehman *et al.* 2011). Genes related to carbohydrate transport were also underexpressed, including *HXT1*, *HXT5*, and *HXT10* (*P <* 1E–4), and are representative of 9.09% of all carbohydrate transport genes in the genome. The oxidative stress response genes comprise 8.57% of the 35 underexpressed genes, while all oxidative stress response genes represent 1.10% of the entire genome, and the carbohydrate transport genes represent 8.57% of the underexpressed genes, as compared to the 0.53% of the genome that all carbohydrate transport proteins make up (Figure 1A). Other notable underexpressed groups include transmembrane transport (five genes, *P <* 0.001), reciprocal meiotic recombination (two genes, *P <* 0.01), oxidation–reduction process (five genes, *P <* 0.01), electron transport chain (two genes, *P <* 0.01), and response to stress (three genes, *P <* 0.01) (see Figure 1A and Figure S1). On the other hand, 39 genes are overexpressed when kap108 is absent. GO analysis done using YEASTRACT (Teixeira *et al.* 2006, 2014; Monteiro *et al.* 2008; Abdulrehman *et al.* 2011) reveals that four of these genes are related to cytogamy, or cell fusion in mating. They represent 50% of cytogamy genes in the yeast genome, and include *FIG1*, *FIG2*, *STE2*, and *FUS2* (*P <* 1E–9). These four genes are representative of 10.26% of the overexpressed genes, whereas all cytogamy genes constitute only 0.13% of the yeast genome (Figure 1B). Other notable overexpressed groups include arginine biosynthetic process (two genes, *P <* 1E–4), removal of superoxide radicals (two genes, *P <* 1E–5), response to pheromone (two genes, *P <* 0.001), and metabolic process (five genes, *P <* 0.01) (see Figure 1B and Figure S2).

To determine how the absence of Kap108 affects gene expression normally induced by oxidative stress, we extracted mRNA from wild-type and *kap108∆* mutant cells after 10 min and 60 min exposure to oxidative stress, and 60 min after the stress was removed. Two channel microarray analyses were then performed to compare expression in wild-type and *kap108∆* cells at each time point. To identify genes undergoing differential expression, we used a filter that selected for genes exhibiting at least a 40% difference in expression in at least one of the three timepoints (Table S2). The filtered gene list comprises 511 of the 6253 genes considered. YEASTRACT GO analysis (Teixeira *et al.* 2006, 2014; Monteiro *et al.* 2008; Abdulrehman *et al.* 2011) of the list of filtered genes shows that overrepresented functions from this group include cellular response to stress (*P <* 1E–12), oxidation–reduction processes (*P <* 1E–9), iron ion homeostasis (*P <* 1E–7), ascospore wall assembly (*P <* 1E–5), transmembrane transport (*P <* 1E–4), and cytogamy (*P <* 1E–4) (Figure 2A, see also Figure S3 for the full GO analysis). Genes involved in response to stress made up 7.45% of these filtered genes (*i.e.*, 38 out of 510 genes, with *PFF1* excluded due to its failure to be recognized by YEASTRACT), while this function makes up about 2.41% of all genes in the genome. Similarly, genes involved with oxidation–reduction processes make up 4.67% of the genome, while they represent 10.0% (51 genes) of the filtered genes. There were 11 filtered iron ion homeostasis genes, making up 2.16% of the list, while this function represents just 0.42% of the genome. Ascospore wall assembly was represented by 12 genes, and made up 2.35% of the gene list, while in the genome it represents only 0.69% of all genes. Transmembrane transport and cytogamy made up 6.27% (32 genes), and 0.78% (four genes) of the filtered gene list, respectively, but comprise only 3.52% and 0.13% of the genome. Finally, sporulation, meiosis, and pheromone-dependent signal transduction involved in conjugation with cellular fusion made up 3.14% (16 genes), 3.73% (19 genes), and 1.18% (6 genes) of the filtered gene list, respectively, while they comprise just 1.63%, 2.13%, and 0.42% of the genome. These data suggest that Kap108 is essential for normal expression of genes encoding proteins involved in these functions either during or after exposure to oxidative stress.

To look more closely at how differential expression was changing for this group of filtered genes, 27 clusters were formed based on whether differential expression increased, decreased, or did not change between time points. These 27 clusters are representative of every possible pattern of change over the four time points (preoxidation, 10 min oxidation, 60 min oxidation, 60 min postoxidation), and are named accordingly. For example, the cluster “UpNcDn” would contain all genes for which the ratio between mutant and wild-type cells increased between time 0 and time 10 (“Up”), did not change between time 10 and time 60 (“Nc”), and went down between time 60 and time 120 (“Dn”). To be classified as an increase or decrease, differential expression between wild-type and mutant cells had to change by at least 20% between time points. The ratios of mutant to wild-type expression for *FIG1*, *FRE4*, and *PRM6*—noteworthy genes from the UpNcDn, NcNcUp, and NcNcDn clusters, respectively—are shown in Figure 2B as an example of the way this clustering method detects patterns.

Overall, at least one gene filled 25 out of the 27 possible clusters (Figure S4). To examine which biological patterns are overrepresented in each cluster, YEASTRACT was used to perform a GO analysis of each cluster (Teixeira *et al.* 2006, 2014; Monteiro *et al.* 2008; Abdulrehman *et al.* 2011). Notable groups revealed by this analysis are outlined in Table 1, while data for all clusters that contained genes are available in Figure S4. Prominent biological functions identified within these clusters include those related to sporulation, cytogamy, stress response, cellular transport, oxidation–reduction processes, and protein production and degradation. The largest clusters included NcNcUp (241 genes) and NcNcDn (50 genes), which contain genes that did not exhibit a divergence in differential expression until after the oxidative stress was removed. Additionally, in many other clusters—including UpNcDn (cytogamy: *FIG1*, *FIG2*, *STE2*), NcUpDn (cellular fusion: *PRM2*), and NcDnUp (stress response: *HSP26*, *AAD4*, *HSP42*, *HSP72*, *SSA4*, *MTL1*, *DDR2*; protein folding: *HSP26*, *SSA4*, *BTN2*, *APJ1*, *CUR1*; oxidation–reduction processes: *HBN1*, *YCR102C*, *AAD4*, *TSA2*, *AAD16*, *YKL070W*, *YKL071W*, *ECM4*, *ALD3*, *OYE3*)—genes related to the biological functions listed above generally showed the greatest change to differential expression after oxidative stress was removed (Figure S4). This is further emphasized by the fact that raising the clustering cutoff to 30% increases the number of genes in NcNcUp and NcNcDn to 270 and 56, respectively; the increase in cutoff stringency results in more genes failing to undergo changes to differential expression until the final time point. These data suggest that many of the major changes to differential expression between wild-type and *kap108∆* cells occur either slowly or after oxidative stress has been removed.

**Table 1:** List of biological functions that undergo changes in differential gene expression between *kap108∆* and wild-type yeast cells under changing conditions of oxidative stress

Function	Clusters	GO Term – Genes With Function in Cluster (Total Genes in Cluster)	Genes	*P*-Value
Sporulation/ascospore formation	UpUpDn	Ascospore wall assembly – 2 (15)	MPC54, MUM3	*P* < 1E–4
	UpNcNc	Ascospore formation – 2 (8)	SPO16, SPS4	*P* < 1E–4
	NcDnDn	Ascospore formation – 2 (3)	SPO74, YOR338W	*P* < 1E–6
		Sporulation resulting in the formation of a cellular spore – 2 (3)	SPO74, IME4	*P* < 1E–5
Meiosis and cytogamy	UpNcNc	Meiosis – 3 (8)	ZIP2, SPO16, SPS4	*P* < 1E–5
	UpNcDn	Cytogamy – 3 (17)	FIG1, FIG2, STE2	*P* < 1E–8
	UpDnDn	Conjugation with cellular fusion – 1 (5)	ASG7	*P* < 1E–4
		Response to pheromone – 1 (5)	BAR1	*P* < 1E–4
	NcNcDn	Karyogamy involved in conjugation with cellular fusion – 3 (50)	KAR4, FUS2, PRM3	*P* < 1E–5
		Plasma membrane fusion involved in cytogamy – 1 (50)	PRM1	*P* = 0
Response to stress	UpDnUp	Response to stress – 3 (19)	MRK1, HSP12, XBP1	*P* < 0.001
		Cellular response to oxidative stress – 2 (19)	HSP12, GPX1	*P* < 0.001
	NcUpNc	Response to stress – 3 (12)	RTA1, WSC4, DAN1	*P* < 1E–4
	NcNcUp	Response to stress – 15 (241)	PAU3, TPS2, TIR1, DAK2, FMP43, PAU15, DAN4, PAU17, RIM11, DDR48, TPS3, PAU19, ZEO1, PAU21, PAU22	*P* < 1E–4
	NcDnUp	Response to stress – 7 (44)	HSP26, AAD4, HSP42, HSP78, SSA4, MTL1, DDR2	*P* < 1E–5
Cellular transport oxidation-reduction process	DnUpNc	Response to stress – 2 (6)	HSP33, HSP32	*P* < 0.001
	DnDnUp	Cellular response to oxidative stress – 2 (10)	FRM2, SRX1	*P* < 0.001
	UpNcUp	Transmembrane transport – 4 (21)	YDL199C, HXT5, HXT14, DIP5	*P* < 0.001
	NcNcUp	Transmembrane transport – 17 (241)	MAL31, GEX1, YCF1, PIC2, HXT10, YFL054C, YGL114W, TPO2, ARN2, YHK8, MRS4, ATR1, MMT1, FET4, POR1, TPO4, MMT2	*P* < 0.001
		Ion transport (many – Fe-related) – 14 (241)	GEX1, FIT1, ARN1, ARN2, YHK8, FRE1, MMT1, FET4, POR1, ATO2, FRE4, FRE3, MFM1, MMT2	*P* < 1E–5
	NcDnUp	Oxidation-reduction process – 10 (44)	HBN1, YCR102C, AAD4, TSA2, AAD16, YKL070W,YKL071W, ECM4, ALD3, OYE3	*P* < 1E–6
	DnNcUp	Oxidation-reduction process – 3 (8)	SOR1, SOR2, FRE2	*P* < 0.001
	DnDnUp	Oxidation-reduction process – 4 (10)	FRM2, AAD6, SRX1, AAD15	*P* < 1E–4
	NcNcUp	Oxidation-reduction process – 23 (241)	ZTA1, ADH5, GPX2, AAD3, SFA1, TRR1, SER3, OLE1, AIM17, SOD1, YJR149W, AHP1, HMX1, FRE1, TSA1, CYB2, YML131W, ARA2, YNL134C, FRE4, GRE2, IDH2, FRE3	*P* < 1E–4
Protein synthesis, modification, and degradation	NcNcDn	Ribosome biogenesis – 9 (50)	UTP20, RSA4, AMP3, UTP10, RIX7, UTP13, NOG2, RCLI, RRS1	*P* < 1E–6
		rRNA processing – 6 (50)	UTP20, SSB1, NSR1, IMP3, UTP10, UTP13	*P* < 0.001
	NcDnUp	Protein folding – 5 (44)	HSP26, SSA4, BTN2, APJ1, CUR1	*P* < 1E–4
	DnUpNc	Proteolysis – 3 (6)	SNO4, HSP33, HSP32	*P* < 1E–5

For each cluster in which each function is found, YEASTRACT GO terms, gene counts (both for the function and the total within the cluster), and gene names are given. *P*-values reflect the significance of the difference in frequency of genes within the GO functional group compared to the frequency of genes with that GO term found in the genome. GO, gene ontology.

## Discussion

In this study, we explored changes in gene expression in cells lacking the karyopherin Kap108, both in the absence and the presence of oxidative stress. Using microarray analysis, we observe that the absence of Kap108 results in changes in expression of over 70 genes under normal growth conditions, and that adding or removing oxidative stresses results in variations in expression of more than 500 genes between wild-type and *kap108*Δ cells. Using clustering and GO analysis, we have identified a number of cellular functions likely to be affected by the absence of *kap108*.

Genes related to responses to oxidative stress were underexpressed in the *kap108∆* mutants under normal conditions. These results suggest that Kap108 facilitates proper cellular response to oxidative stress in yeast cells, perhaps by transporting as yet unidentified transcription factors that act as transcriptional activators for a gene, or genes, necessary for producing an appropriate response. Analysis of gene expression in *kap108∆* and wild-type strains after the addition of oxidative stress reveals that 511 genes related to six biological functions—stress response, oxidation–reduction process, iron ion homeostasis, sporulation, transmembrane transport, and cytogamy—have their differential expression changed by at least 40% between one or more of the three time points considered. This suggests that the addition of oxidative stress affects the performance of these functions in *kap108∆* cells differently than it does in wild-type cells. Interestingly, grouping these genes into clusters based on how differential expression changes between each time point reveals that a majority of the genes do not demonstrate differential expression changes between wild-type and *kap108*Δ cells upon introduction of oxidative stress, but instead diverge in gene expression patterns when the stressor is removed, with most appearing in clusters NcNcUp and NcNcDn. Additionally, genes related to these six functions found in other clusters also demonstrate the greatest change to differential expression in the final time point. The fact that the greatest change to the ratio of mutant to wild-type expression occurs after the oxidative stress has been removed suggests that *kap108∆* cells are not able to readjust the performance of these functions appropriately, indicating that the transport function of Kap108 may be more important for recovery from oxidative stress than immediate response to stress.

An explanation for such a failure for a gene to readjust its expression after the removal of a stressor is that Kap108 not only transports factors responsible for activating or repressing genes involved in oxidative stress response, but also transports proteins that facilitate a return to “nonstress” patterns of gene expression. If wild-type cells returned to normal patterns of gene expression while *kap108∆* mutant cells did not, this would show up in the data as large changes to differential expression following the removal of oxidative stress, which is what is occurring for those genes expressing the frequently observed NcNcUp or NcNcDn patterns. Interestingly, yeast exposed to hydrogen peroxide induced oxidative stress often undergo programmed cell death (Madeo *et al.* 1999), a process that requires complex communication among the cytoplasmic, nuclear, and mitochondrial compartments in the cell (see Shaughnessy *et al.* 2014). Recent observations have implicated the regulation of nucleocytoplasmic transport of specific proteins in the mediation of the apoptotic response to oxidative stress (Cooper *et al.* 2012). An interesting future avenue of work will be to determine if links exist between the changes in gene expression observed in our screen, and those observed in apoptotic cells.

Previous screens of gene expression in yeast exposed to oxidative stress have revealed the expression of genes related to a number of different biological functions to be impacted, including the repression of ribosome synthesis and the activation of oxidative stress response, cellular redox reactions, protein folding and degradation, iron homeostasis, and transmembrane and ion transport (Gasch *et al.* 2000; O’Doherty *et al.* 2013). These are all functions performed by proteins encoded by genes that appeared in this study, and thus further support the idea that they are functions that do not properly recover in *kap108∆* cells. If ribosome synthesis is repressed, for example, wild-type expression of genes related to this function should increase once oxidative stress is removed, and thus decrease the ratio of mutant to wild-type expression at this time point. This is exactly what we observe: ribosomal synthesis genes appear in the NcNcDn cluster. Similarly, if the other functions undergo increased expression in wild-type yeast cells in response to oxidative stress, then removing the stress should cause that expression to go back down and, consequently, for the ratio of mutant to wild-type expression to go up. This is again what we observe: NcNcUp has far more genes than any other, and the third largest group is NcDnUp, and these clusters contain genes related to stress response, ion transport, transmembrane transport, oxidation–reduction processes, and protein folding.

We report here that genes related to mating, cytogamy, and some metabolic processes (see Figure 1B) are upregulated in *kap108∆* mutants as compared to wild-type cells, even under conditions lacking oxidative or other stresses. A constitutive increase in expression in cells lacking a nuclear import factor was not entirely expected. The simplest explanation for this increase is that Kap108 facilitates the transport of at least one transcription factor that inhibits expression of mating genes identified in this study. Without Kap108, expression of these mating genes would no longer be inhibited, and would increase as a result. A second, more indirect explanation is that Kap108 imports protein(s) into the nucleus that inhibit activators of mating genes. Ste12, for example, is a transcription factor that has been shown to enhance the expression of mating genes (Fields and Herskowitz 1985; Dolan *et al.* 1989), but we have observed that the nuclear localization of Ste12 does not change between wild-type and *kap108∆* cells (data not shown). While this suggests that Ste12 is not the cargo of Kap108, this does not mean that the absence of Kap108 does not alter Ste12 activity; Kap108 may transport another factor that inhibits transcription activation by Ste12, such as Kss1, a protein that has been shown to bind Ste12 directly and repress its function (Bardwell *et al.* 1998), and has also been shown to be concentrated in the nucleus (Ma *et al.* 1995). Future experiments will explore the translocation of various nuclear proteins, including Kss1, which may function to modulate the expression of diverse sets of genes through functional interactions with Kap108.

## 

## Supplementary Material

Supporting Information
