# Supplementary material for: Microarray Analysis of Gene Expression in Saccharomyces cerevisiae kap108Δ Mutants upon Addition of Oxidative Stress
Source: G3 (Bethesda). 2016 Feb 17;6(4):1131–9. doi: 10.1534/g3.116.027011 (PMC4825647; doi:10.1534/g3.116.027011)
Supplement: Supporting Information [file supp_6_4_1131__index.html]

Microarray Analysis of Gene Expression in Saccharomyces cerevisiae kap108Δ Mutants upon Addition of Oxidative Stress — Supporting Information 

# Microarray Analysis of Gene Expression in *Saccharomyces cerevisiae kap108*Δ Mutants upon Addition of Oxidative Stress

## Supporting Information for Belanger *et al.*, 2016

**Files in this Data Supplement:**

- Figure S1 - YEASTRACT Gene Ontology analysis of underexpressed genes in mutant cells under normal growth conditions. (.pdf, 2063 KB)
- Figure S2 - YEASTRACT Gene Ontology analysis of overexpressed genes in mutant cells under normal growth conditions. (.pdf, 2339 KB)
- Figure S5 - All custom code used in this study. All code was implemented in R. (.pdf, 20 KB)
- Figure S3 - YEASTRACT Gene Ontology analysis of genes filtered based on having at least a 40% change to differential gene expression in at least one of three post-oxidation time points as compared to normal growth conditions. (.zip, 217 MB)
- Figure S4 - : Excel file containing all 27 clusters and the genes that comprise them, as well as graphical depictions of how differential expression for each gene changes between the four time points considered, summaries of YEASTRACT GO analyses, and links to YEASTRACT GO analyses for each individual cluster. (.xlsx, 211 KB)
- Table S1 - List of genes with 1.5 fold or greater difference in expression between *kap108 Δ* (M) and wild-type (WT) cells (p<0.05 by unpaired t-test with Bonferonni correction). (.docx, 83 KB)
