# Supplementary material for: Microarray Analysis of Gene Expression in Saccharomyces cerevisiae kap108Δ Mutants upon Addition of Oxidative Stress
Source: G3 (Bethesda). 2016 Feb 17;6(4):1131–9. doi: 10.1534/g3.116.027011 (PMC4825647; doi:10.1534/g3.116.027011)
Supplement: Supporting Information [file supp_g3.116.027011_FigureS1.pdf]

GO ID	GO term	Level	% in user set	% in Yeastract	p-value	Genes/ORF
GO:0061424	positive regulation of peroxisome organization by positive regulation of transcription from RNA polymerase II promoter	9	2.86%	100.00%	0.000000000000000	ADR1
GO:0061425	positive regulation of ethanol catabolic process by positive regulation of transcription from RNA polymerase II promoter	8	2.86%	100.00%	0.000000000000000	ADR1
GO:0097235	positive regulation of fatty acid beta-oxidation by positive regulation of transcript	9	2.86%	100.00%	0.000000000000000	ADR1

	[illegible]ion from RNA polymerase II promoter					
GO:0005999	xylulose biosynthetic process	8	2.86%	100.00%	0.000000000000000	XYL2
GO:0019569	L-arabinose catabolic process to xylulose 5-phosphate	7	2.86%	100.00%	0.000000000000000	XYL2
GO:0033194	response to hydroperoxide	5	2.86%	100.00%	0.000000000000000	TSA1
GO:0042262	DNA protection	5	2.86%	100.00%	0.000000000000000	TSA1
GO:0035952	negative regulation of oligopeptide transport by negative regulation of transcription from RNA polymerase II promoter	7	2.86%	100.00%	0.000000000000000	CUP9
GO:0015[illegible]	glucose transport	7	5.71%	25.00%	0.000006043706[illegible]	HXT1 HXT5

[illegible]758	[illegible]				[illegible]293	[illegible]
GO:0008643	carbohydrate transport	4	8.57%	9.09%	0.000018298051212	HXT10 HXT1 HXT5
GO:0015876	acetyl-CoA transport	6	2.86%	50.00%	0.000023609752613	YBR219C
GO:0045980	negative regulation of nucleotide metabolic process	8	2.86%	50.00%	0.000023609752613	INH1
GO:0061429	positive regulation of transcription from RNA polymerase II promoter by oleic acid	9	2.86%	50.00%	0.000023609752613	ADR1
GO:0080058	protein deglutathionylation	7	2.86%	50.00%	0.000023609752613	TRX2
GO:0015671	oxygen transport	5	2.86%	50.00%	0.000023609752613	YHB1
GO:0015761	mannose transport	7	2.86%	50.00%	0.000023609752613	HXT1
GO:0009081	branched-chain amino acid metabolic process	5	2.86%	50.00%	0.000023609752613	BAT2
GO:[illegible]	D-xylose [illegible]	[illegible]	[illegible]	[illegible]	0.00002[illegible]	[illegible]

GO:0042732	[illegible] metabolic process	8	2.86%	50.00%	0.000023609752613	XYL2
GO:0031503	protein complex localization	5	2.86%	50.00%	0.000023609752613	ATG34
GO:0015805	S-adenosyl-L-methionine transport	5	2.86%	50.00%	0.000023609752613	SAM3
GO:0097201	negative regulation of transcription from RNA polymerase II promoter in response to stress	7	2.86%	50.00%	0.000023609752613	ROX1
GO:0008645	hexose transport	6	5.71%	12.50%	0.000058822796543	HXT10 HXT5
GO:0032780	negative regulation of ATPase activity	7	2.86%	33.33%	0.000070609725060	INH1
GO:0061410	positive regulation of transcription from RNA polymerase II promoter in response to [illegible]	8	2.86%	33.33%	0.000070609725060	ADR1

	to ethanol					
GO:0006089	lactate metabolic process	5	2.86%	33.33%	0.000070609725060	CYB2
GO:0035955	negative regulation of dipeptide transport by negative regulation of transcription from RNA polymerase II promoter	8	2.86%	33.33%	0.000070609725060	CUP9
GO:0006075	(1->3)-beta-D-glucan biosynthetic process	9	2.86%	25.00%	0.000140781869353	GSC2
GO:0015847	putrescine transport	5	2.86%	25.00%	0.000140781869353	SAM3
GO:0015848	spermidine transport	5	2.86%	25.00%	0.000140781869353	SAM3
GO:0031936	negative regulation of chromatin silencing	7	2.86%	20.00%	0.000233909613649	ADR1
GO:0006662	glycerol ether metabolic process	5	2.86%	20.00%	0.000233909613649	TRX2
	response					

GO:0009636	response to toxic substance	4	2.86%	20.00%	0.000233909613649	YHB1
GO:0034599	cellular response to oxidative stress	5	8.57%	4.35%	0.000340864023043	GPX2 TRX2 TSA1
GO:0009263	deoxyribonucleotide biosynthetic process	6	2.86%	16.67%	0.000349777853645	TRX2
GO:0009099	valine biosynthetic process	7	2.86%	16.67%	0.000349777853645	BAT2
GO:0045454	cell redox homeostasis	5	5.71%	6.67%	0.000406756383883	TRX2 TSA1
GO:0071400	cellular response to oleic acid	7	2.86%	14.29%	0.000488172944021	ADR1
GO:0009098	leucine biosynthetic process	7	2.86%	14.29%	0.000488172944021	BAT2
GO:0055085	transmembrane transport	4	14.29%	2.27%	0.000631844658496	HXT10 VPS73 HXT1 HXT5 SAM3
GO:0007130	synaptonemal complex assembly	5	2.86%	12.50%	0.000648882689930	ZIP2
GO:0000103	sulfate assimilation	5	2.86%	12.50%	0.000648882689930	TRX2
	regulation of [illegible]					

GO:0043618	[illegible]n of transcription from RNA polymerase II promoter in response to stress	6	2.86%	12.50%	0.000648882689930	ROX1
GO:0009083	branched-chain amino acid catabolic process	6	2.86%	11.11%	0.000831696338528	BAT2
GO:0019509	L-methionine salvage from methylthioadenosine	7	2.86%	11.11%	0.000831696338528	BAT2
GO:0006979	response to oxidative stress	4	2.86%	10.00%	0.001036404570552	GPX2
GO:0007129	synapsis	5	2.86%	10.00%	0.001036404570552	ZIP2
GO:0009097	isoleucine biosynthetic process	7	2.86%	10.00%	0.001036404570552	BAT2
GO:0007131	reciprocal meiotic recombination	4	5.71%	4.08%	0.001731250576716	ECM11 ZIP2
GO:0006325	chromatin organization	6	2.86%	7.69%	0.001779824902647	ADR1
[illegible]					[illegible]	

GO:0015918	sterol transport	5	2.86%	7.69%	0.001779824902647	PRY1
GO:0009082	branched-chain amino acid biosynthetic process	6	2.86%	7.69%	0.001779824902647	BAT2
GO:0022900	electron transport chain	5	5.71%	4.00%	0.001835561965672	TRX2 CYB2
GO:0008360	regulation of cell shape	4	2.86%	7.14%	0.002070046655082	GSC2
GO:0055114	oxidation-reduction process	4	14.29%	1.71%	0.002722113383082	GPX2 YHB1 XYL2 TSA1 CYB2
GO:0034401	regulation of transcription by chromatin organization	7	2.86%	5.56%	0.003437623606021	ADR1
GO:0006913	nucleocytoplasmic transport	6	2.86%	5.56%	0.003437623606021	SXM1
GO:0000011	vacuole inheritance	6	2.86%	5.56%	0.003437623606021	TRX2
GO:0000077	DNA damage checkpoint	6	2.86%	5.26%	0.003830196150925	TSA1
GO:0003333	amino acid transmembrane transport	6	2.86%	4.17%	0.006087373251224	SAM3

	transport					
GO:0006950	response to stress	3	8.57%	1.99%	0.006165219896777	YHB1 DDR48 ZEO1
GO:0007031	peroxisome organization	5	2.86%	3.85%	0.007124023486029	ADR1
GO:0006890	retrograde vesicle-mediated transport, Golgi to ER	5	2.86%	3.57%	0.008234865191594	TRX2
GO:0006461	protein complex assembly	5	2.86%	3.57%	0.008234865191594	ATG34
GO:0042144	vacuole fusion, non-autophagic	6	2.86%	3.33%	0.009418424238044	TRX2
GO:0006406	mRNA export from nucleus	6	2.86%	2.94%	0.011997899532504	SXM1
GO:0006810	transport	3	22.86%	1.01%	0.012628300530402	SXM1 HXT10 VPS73 HXT1 HXT5 ATG34CRC1 SAM3
GO:0007005	mitochondrion organization	5	2.86%	2.86%	0.012685969560789	NCA3
GO:0006631	fatty acid metabolic process	5	2.86%	2.78%	0.013390968500723	CRC1
GO:0001302	replicative cell aging	5	2.86%	2.56%	0.015605808475155	TSA1
GO:	amino [illegible]				0.01716[illegible]	

0006865	acid transport	5	2.86%	2.44%	3861909710	SAM3
GO:0030476	ascospore wall assembly	4	2.86%	2.33%	0.018785537180183	GSC2
GO:0006623	protein targeting to vacuole	5	2.86%	2.08%	0.023109408502379	VPS73
GO:0006914	autophagy	5	2.86%	1.96%	0.025882195655667	ATG34
GO:0051028	mRNA transport	5	2.86%	1.59%	0.038218614763577	SXM1
GO:0015031	protein transport	4	11.43%	1.03%	0.040660817610133	SXM1 VPS73 TRX2 ATG34
GO:0007059	chromosome segregation	4	2.86%	1.45%	0.045077931262748	ZIP2
GO:0008150	biological_process	1	25.71%	0.76%	0.053670143171499	YBR219C YCL001W-B YGL204C YGR273CANS1 YHR214W YMR317W RRT8 YOR365C
GO:0006888	ER to Golgi vesicle-mediated transport	5	2.86%	1.25%	0.058723460737773	TRX2
GO:0000122	negative regulation of transcription from RNA polymerase II promoter	9	2.86%	1.25%	0.058723460737773	ROX1
GO:	intracellu				0.06806	

GO:0006886	[illegible]lar protein transport	5	2.86%	1.15%	[illegible]1962051667	SXM1
GO:0031505	fungal-type cell wall organization	5	2.86%	1.10%	0.073606892572329	ZEO1
GO:0006457	protein folding	6	2.86%	1.06%	0.077859350072223	TSA1
GO:0008652	cellular amino acid biosynthetic process	5	2.86%	1.02%	0.083648410994306	BAT2
GO:0005975	carbohydrate metabolic process	4	2.86%	1.02%	0.083648410994306	XYL2
GO:0030435	sporulation resulting in formation of a cellular spore	4	2.86%	0.98%	0.089567056549660	GSC2
GO:0007126	meiosis	4	2.86%	0.75%	0.139102618291985	ZIP2
GO:0006281	DNA repair	6	2.86%	0.53%	0.235009273912799	DDR48
GO:0006355	regulation of transcription, DNA-dependent	7	8.57%	0.59%	0.238892121294809	ADR1 CUP9 ROX1
GO:[illegible]	metaboli[illegible]	[illegible]	[illegible]	[illegible]	0.46590[illegible]	[illegible]

GO:0008152	[illegible] metabolic process	2	2.86%	0.32%	[illegible]1468640063	BAT2
GO:0007049	cell cycle	4	2.86%	0.32%	0.467563754340974	ZIP2
GO:0006351	transcription, DNA-dependent	6	5.71%	0.38%	0.485868348200102	ADR1 ROX1
