# Supplementary material for: Microarray Analysis of Gene Expression in Saccharomyces cerevisiae kap108Δ Mutants upon Addition of Oxidative Stress
Source: G3 (Bethesda). 2016 Feb 17;6(4):1131–9. doi: 10.1534/g3.116.027011 (PMC4825647; doi:10.1534/g3.116.027011)
Supplement: Supporting Information [file supp_g3.116.027011_FigureS2.pdf]

GO ID	GO term	Level	% in user set	% in Yeastract	p-value	Genes/ORF
GO:0030031	cell projection assembly	5	2.56%	100.00%	0.000000000000	STE2
GO:0010273	detoxification of copper ion	5	5.13%	100.00%	0.000000000000	CUP1-1 CUP1-2
GO:0071585	detoxification of cadmium ion	5	5.13%	100.00%	0.000000000000	CUP1-1 CUP1-2
GO:0009442	allantoin assimilation pathway	7	2.56%	100.00%	0.000000000000	DAL1
GO:0035445	borate transmembrane transport	6	2.56%	100.00%	0.000000000000	YMR279C
GO:0000755	cytogamy	5	10.26%	50.00%	0.000000021218 5	FIG1 FIG2 STE2 FUS2
GO:0015886	heme transport	5	5.13%	50.00%	0.000000610749342	AAC3 PUG1
GO:0046688	response to copper ion	6	5.13%	40.00%	0.000001521065305	CUP1-1 CUP1-2

GO:0019430	removal of superoxide radicals	6	5.13%	33.33%	0.000003030560346	CUP1-1 CUP1-2
GO:0000753	cell morphogenesis involved in conjugation with cellular fusion	4	5.13%	25.00%	0.000008421158437	FIG1 FIG2
GO:0006526	arginine biosynthetic process	7	5.13%	18.18%	0.000024530468681	ARG5,6 CPA2
GO:0009061	anaerobic respiration	7	2.56%	50.00%	0.000029403070061	AAC3
GO:0044182	filamentous growth of a population of unicellular organisms	4	2.56%	50.00%	0.000029403070061	FIG2
GO:0045905	positive regulation of translational termination	8	2.56%	50.00%	0.000029403070061	ANB1
GO:007[illegible]	synaptonemal comple[illegible]	[illegible]	[illegible]	50.00[illegible]	0.0000[illegible]	

[illegible]0193	[illegible]x organization	6	2.56%	[illegible]%	[illegible]29403070061	GMC2
GO:0000742	karyogamy involved in conjugation with cellular fusion	4	5.13%	13.33%	0.000066622476605	PRM2 FUS2
GO:0035948	positive regulation of gluconeogenesis by positive regulation of transcription from RNA polymerase II promoter	9	2.56%	33.33%	0.000087902669302	SIP4
GO:0045722	positive regulation of gluconeogenesis	8	2.56%	33.33%	0.000087902669302	SIP4
GO:0061414	positive regulation of transcription from RNA polymerase II promoter by a[illegible]	9	2.56%	33.33%	0.000087902669302	SIP4

	er by a nonfermentable carbon source					
GO:2000218	negative regulation of invasive growth in response to glucose limitation	9	2.56%	33.33%	0.0000879026 69302	SIP4
GO:0045901	positive regulation of translational elongation	9	2.56%	33.33%	0.0000879026 69302	ANB1
GO:0042631	cellular response to water deprivation	5	2.56%	33.33%	0.0000879026 69302	SIP18
GO:0006592	ornithine biosynthetic process	7	2.56%	25.00%	0.0001751945 89262	ARG5,6
GO:0008612	peptidyl-lysine modification to hypusine	7	2.56%	25.00%	0.0001751945 89262	ANB1
	regulati					

GO:0031385	on of termination of mating projection growth	6	2.56%	25.00%	0.000175194589262	FUS2
GO:0000746	conjugation	4	2.56%	20.00%	0.000290976938566	FIG2
GO:0051083	'de novo' cotranslational protein folding	8	2.56%	20.00%	0.000290976938566	SSB1
GO:0006452	translational frameshifting	7	2.56%	20.00%	0.000290976938566	ANB1
GO:0070898	RNA polymerase III transcriptional preinitiation complex assembly	8	2.56%	16.67%	0.000434950127676	NHP6b
GO:0051260	protein homooligomerization	7	2.56%	16.67%	0.000434950127676	STE2
GO:0046513	ceramide biosynthetic process	7	2.56%	16.67%	0.000434950127676	LAC1
GO:005[illegible]	meiotic recomb[illegible]	5	2.56%	16.67[illegible]	0.0004[illegible]	

[illegible]1598	[illegible]ination checkpoint	5	2.56%	[illegible]%	[illegible]34950127676	MEK1
GO:0015802	basic amino acid transport	6	2.56%	14.29%	0.000606816853734	VBA3
GO:0042149	cellular response to glucose starvation	6	2.56%	14.29%	0.000606816853734	SSB1
GO:0007186	G-protein coupled receptor signaling pathway	6	2.56%	14.29%	0.000606816853734	STE2
GO:0040020	regulation of meiosis	6	2.56%	14.29%	0.000606816853734	RCK1
GO:0000256	allantoin catabolic process	6	2.56%	14.29%	0.000606816853734	DAL1
GO:0035023	regulation of Rho protein signal transduction	8	2.56%	14.29%	0.000606816853734	FUS2
GO:0007155	cell adhesion	3	2.56%	12.50%	0.000806282085500	FIG2

[illegible]						
GO:0006144	purine nucleobase metabolic process	6	2.56%	12.50%	0.000806282085500	DAL1
GO:0019236	response to pheromone	5	5.13%	5.88%	0.000815163770371	FIG2 STE2
GO:0006278	RNA-dependent DNA replication	7	2.56%	11.11%	0.001033053048373	YIL082W-A
GO:0000054	ribosomal subunit export from nucleus	5	2.56%	8.33%	0.001874306115296	SSB1
GO:0006415	translational termination	7	2.56%	7.69%	0.002207416870328	SSB1
GO:0006446	regulation of translational initiation	8	2.56%	7.69%	0.002207416870328	TIF2
GO:0006807	nitrogen compound metabolic process	3	2.56%	7.69%	0.002207416870328	CPA2
GO:000836[illegible]	regulation of cell[illegible]	4	2.56%	7.14%	0.0025664028[illegible]	FIG2

[illegible]8360	[illegible] cell shape				[illegible]15235	FIG2
GO:0006098	pentose-phosphate shunt	6	2.56%	6.67%	0.002950984405793	PGI1
GO:0006450	regulation of translational fidelity	4	2.56%	5.88%	0.003795827104234	SSB1
GO:0006407	rRNA export from nucleus	5	2.56%	5.56%	0.004255539838210	RPS26a
GO:0006094	gluconeogenesis	8	2.56%	5.26%	0.004739751441741	PGI1
GO:0008152	metabolic process	2	12.82%	1.58%	0.006981523208190	DSF1 ARG5,6 YIL082W-A CPA2 YNR073C
GO:0006096	glycolysis	5	2.56%	3.85%	0.008792873016127	PGI1
GO:0006520	cellular amino acid metabolic process	4	2.56%	3.85%	0.008792873016127	ARG5,6
GO:0000750	pheromone-dependent signal transduction involved in [illegible]	5	2.56%	3.85%	0.008792873016127	STE2

	conjugation with cellular fusion					
GO:0002181	cytoplasmic translation	7	7.69%	1.80%	0.012796772676068	SSB1 RPS26a RPP0
GO:0000027	ribosomal large subunit assembly	6	2.56%	3.03%	0.013944235782431	RPP0
GO:0032197	transposition, RNA-mediated	5	5.13%	2.11%	0.014976213565946	YGR109W-A YIL082W-A
GO:0008652	cellular amino acid biosynthetic process	5	5.13%	2.04%	0.016272094431332	ARG5,6 CPA2
GO:0006412	translation	6	10.26%	1.45%	0.016466030904083	SSB1 RPS26a TIF2 ANB1
GO:0001302	replicative cell aging	5	2.56%	2.56%	0.019169502380335	LAC1
GO:0006366	transcription from RNA polymerase II promoter	7	5.13%	1.89%	0.020029363394989	NHP6b SIP4
GO:	amino				0.0210	

[illegible] 0006865	[illegible] acid transport	5	2.56%	2.44%	[illegible]67902899884	VBA3
GO:0001403	invasive growth in response to glucose limitation	7	2.56%	2.27%	0.024056076628461	FIG2
GO:0046797	viral procapsid maturation	4	2.56%	2.27%	0.024056076628461	YIL082W-A
GO:0016310	phosphorylation	6	7.69%	1.46%	0.025219821596876	ARG5,6 RCK1 MEK1
GO:0015074	DNA integration	6	2.56%	2.17%	0.026139311128098	YIL082W-A
GO:0006413	translational initiation	4	2.56%	2.08%	0.028293400374085	TIF2
GO:0055085	transmembrane transport	4	7.69%	1.36%	0.031617065272471	AAC3 VBA3 YMR279C
GO:0006338	chromatin remodeling	8	2.56%	1.92%	0.032807300881554	NHP6b
GO:0006468	protein phosphorylation	7	5.13%	1.50%	0.035973320245152	RCK1 MEK1
GO:000[illegible]	[illegible]	[illegible]	[illegible]	[illegible]	0.0359[illegible]	[illegible]

[illegible]7126	meiosis	4	5.13%	1.50%	[illegible]73320245152	GMC2 MEK1
GO:0008150	biological_process	1	25.64%	0.85%	0.048162620489796	YDR374C DSF1 PRM8 YGR050C YJR146WYKR015C YKR041W YLR179C YNR073C YOR387C
GO:0006950	response to stress	3	5.13%	1.32%	0.049365824746836	SSB1 PUG1
GO:0007165	signal transduction	4	2.56%	1.43%	0.056192761014271	STE2
GO:0055114	oxidation-reduction process	4	7.69%	1.03%	0.074728711234426	DSF1 ARG5,6 YNR073C
GO:0090305	nucleic acid phosphodiester bond hydrolysis	6	2.56%	1.14%	0.083801119088091	YIL082W-A
GO:0006310	DNA recombination	6	2.56%	1.08%	0.092086054444577	YIL082W-A
GO:0006355	regulation of transcription, DNA-dependent	7	10.26%	0.79%	0.143142564075412	NHP6b ARG5,6 SIP4 GMC2
GO:0016192	vesicle-mediated transport	4	2.56%	0.71%	0.179026982560727	PRM8

GO:0006629	lipid metabolic process	4	2.56%	0.68%	0.192933668482838	LAC1
GO:0006508	proteolysis	5	2.56%	0.62%	0.223187231112316	YIL082W-A
GO:0006414	translational elongation	6	5.13%	0.63%	0.254823281886537	ANB1 RPP0
GO:0042254	ribosome biogenesis	5	2.56%	0.56%	0.257931975693649	RPP0
GO:0006281	DNA repair	6	2.56%	0.53%	0.274353053621905	NHP6b
GO:0006364	rRNA processing	6	2.56%	0.51%	0.296925223733152	SSB1
GO:0006974	response to DNA damage stimulus	5	2.56%	0.50%	0.305118020767571	NHP6b
GO:0006351	transcription, DNA-dependent	6	7.69%	0.57%	0.326778166609068	NHP6b SIP4 GMC2
GO:0006810	transport	3	10.26%	0.50%	0.445579061179763	AAC3 VBA3 PRM8 YMR279C
