# Supplementary material for: Microarray Analysis of Gene Expression in Saccharomyces cerevisiae kap108Δ Mutants upon Addition of Oxidative Stress
Source: G3 (Bethesda). 2016 Feb 17;6(4):1131–9. doi: 10.1534/g3.116.027011 (PMC4825647; doi:10.1534/g3.116.027011)
Supplement: Supporting Information [file supp_g3.116.027011_FigureS5.pdf]

```
foldChange<-function(data_subset,col1,col2) {
	#Calculates fold change. Converts log2 values back. Col1
should be M values, col2 should be WT values.
	foldchange<- 2^(col1 - col2)

	return(foldchange)
}
filterGenes<-function(data, data_subset,foldchange,cutoff) {
	#filters genes that have a foldchange greater than the
specified cutoff. Foldchange variable to input should be the output of
the foldchange function above.
	data_subset$foldchange<-foldchange
	data_new<-data[abs(data_subset$foldchange)>cutoff,]

	return(data_new)
}
addFC<-function(data_new,data_new_col1,data_new_col2,fc) {
	#Adds a fold change column
	data_new$fc<-foldChange(data_new,data_new_col1,data_new_col2)

	return(data_new)
}


####################################################


readGSfiles<-function(files_location,cutoff) {
	#Returns gene names with ratios of fold changes between
different time points (10 and 0, 60 and 0, and 120 and 	#0)
	#Assumes 4 files with very specific specifications/formatting
(3 columns: "Gene_Name"; "M#"; "WT#", where # reflects the time
point).
	#Returns 4 ratios for each gene: times 0 to 0, 10 to 0, 60 to
0, and 120 to 0.
	setwd(files_location)
	filelist<-list.files(pattern=".*.txt")
	print(filelist)

	t0<-read.table(filelist[1],header=TRUE)
	t10<-read.table(filelist[2],header=TRUE)
	t60<-read.table(filelist[3],header=TRUE)
	t120<-read.table(filelist[4],header=TRUE)

	t0_sub<-t0[,-1]
	t10_sub<-t10[,-1]
	t60_sub<-t60[,-1]
	t120_sub<-t120[,-1]

	fc_t0<-foldChange(t0_sub,t0_sub$M,t0_sub$WT)
```

```
	genes0<-filterGenes(t0,t0_sub,fc_t0,cutoff)
	genes0<-addFC(genes0,genes0$M,genes0$WT,fc_t0)

	fc_t10<-foldChange(t10_sub,t10_sub$M,t10_sub$WT)
	genes10<-filterGenes(t10,t10_sub,fc_t10,cutoff)
	genes10<-addFC(genes10,genes10$M,genes10$WT,fc_t10)

	fc_t60<-foldChange(t60_sub,t60_sub$M,t60_sub$WT)
	genes60<-filterGenes(t60,t60_sub,fc_t60,cutoff)
	genes60<-addFC(genes60,genes60$M,genes60$WT,fc_t60)

	fc_t120<-foldChange(t120_sub,t120_sub$M,t120_sub$WT)
	genes120<-filterGenes(t120,t120_sub,fc_t120,cutoff)
	genes120<-addFC(genes120,genes120$M,genes120$WT,fc_t120)

	genelist<-list()
	genelist$fc_t0<-genes0
	genelist$fc_t10<-genes10
	genelist$fc_t60<-genes60
	genelist$fc_t120<-genes120

	genelist<-as.data.frame(genelist)

	ratio0_to_0<-genelist$fc_t0.fc/genelist$fc_t0.fc
	ratio10_to_0<-genelist$fc_t10.fc/genelist$fc_t0.fc
	ratio60_to_0<-genelist$fc_t60.fc/genelist$fc_t0.fc
	ratio120_to_0<-genelist$fc_t120.fc/genelist$fc_t0.fc

	data_ratios<-
data.frame(genes0$Gene_Name,ratio0_to_0,fc_t0,ratio10_to_0,fc_t10,rati
o60_to_0,fc_t60,ratio120_to_0,fc_t120)

	return(data_ratios)
}

filterRatios<-function(data_ratios,hcutoff,lcutoff){
	#Returns only genes with AT LEAST one differential expression
ratio (out of all of the ratios calculated in the function above)
above hcutoff or below   lcutoff
	#Use output from readGSfiles() as input for data_ratios
	data_filtered<-data_ratios[data_ratios$ratio10_to_0>hcutoff |
data_ratios$ratio10_to_0<lcutoff |data_ratios$ratio60_to_0>hcutoff |
data_ratios$ratio60_to_0<lcutoff | data_ratios$ratio120_to_0>hcutoff |
data_ratios$ratio120_to_0<lcutoff,]

	return(data_filtered)
}

cluster<-function(data, hcutoff,lcutoff) {
	#Function that creates clusters. hcutoff and lcutoff represent
```

the upper and lower bounds for determining what qualifies as "Up" and "Dn" (i.e. hcutoff = 1.2 and lcutoff = 0.8 would mean to be "Up" the differential expression ratio would have to go up by 1.2 times or more, while to be "Dn" the differential expression ratio would have to go down by 0.8 times or more).

```
        data_final<-list()
        data$ratio1<-data$fc_t10/data$fc_t0
        data$ratio2<-data$fc_t60/data$fc_t10
        data$ratio3<-data$fc_t120/data$fc_t60

        data_final$UpUpUp<-data[data$ratio1 > hcutoff & data
$ratio2>hcutoff&data$ratio3>hcutoff,]
        data_final$UpUpNA<-data[data$ratio1>hcutoff&data
$ratio2>hcutoff & data$ratio3<hcutoff & data$ratio3>lcutoff,]
        data_final$UpUpDown<-data[data$ratio1>hcutoff & data
$ratio2>hcutoff & data$ratio3<lcutoff,]
        #
        data_final$UpNAUp<-data[data$ratio1>hcutoff & data
$ratio2<hcutoff & data$ratio2>lcutoff & data$ratio3>hcutoff,]
        data_final$UpNANA<-data[data$ratio1>hcutoff & data
$ratio2<hcutoff & data$ratio2>lcutoff & data$ratio3<hcutoff&data
$ratio3>lcutoff,]
        data_final$UpNADown<-data[data$ratio1>hcutoff & data
$ratio2<hcutoff & data$ratio2>lcutoff & data$ratio3<lcutoff,]
        #
        data_final$UpDownUp<-data[data$ratio1>hcutoff&data
$ratio2<lcutoff&data$ratio3>hcutoff,]
        data_final$UpDownNA<-data[data$ratio1>hcutoff&data
$ratio2<lcutoff&data$ratio3<hcutoff&data$ratio3>lcutoff,]
        data_final$UpDownDown<-data[data$ratio1>hcutoff & data
$ratio2<lcutoff & data$ratio3 < lcutoff,]
        #
        data_final$NAUpUp<-data[data$ratio1<hcutoff & data
$ratio1>lcutoff & data$ratio2>hcutoff & data$ratio3>hcutoff,]
        data_final$NAUpNA<-data[data$ratio1<hcutoff & data
$ratio1>lcutoff & data$ratio2>hcutoff & data$ratio3<hcutoff &data
$ratio3>lcutoff,]
        data_final$NAUpDown<-data[data$ratio1<hcutoff & data
$ratio1>lcutoff & data$ratio2>hcutoff & data$ratio3<lcutoff,]
        #
        data_final$NANAUp<-data[data$ratio1<hcutoff & data
$ratio1>lcutoff & data$ratio2<hcutoff & data$ratio2>lcutoff & data
$ratio3>hcutoff,]
        data_final$NANANA<-data[data$ratio1<hcutoff & data
$ratio1>lcutoff & data$ratio2<hcutoff & data$ratio2>lcutoff & data
$ratio3<hcutoff & data$ratio3>lcutoff,]
        data_final$NANADown<-data[data$ratio1<hcutoff & data
$ratio1>lcutoff & data$ratio2<hcutoff & data$ratio2>lcutoff & data
$ratio3<lcutoff,]
        #
```

```
        data_final$NADownUp<-data[data$ratio1<hcutoff & data
$ratio1>lcutoff & data$ratio2<lcutoff & data$ratio3>hcutoff,]
        data_final$NADownNA<-data[data$ratio1<hcutoff & data
$ratio1>lcutoff & data$ratio2<lcutoff & data$ratio3<hcutoff & data
$ratio3 > lcutoff,]
        data_final$NADownDown<-data[data$ratio1<hcutoff & data
$ratio1>lcutoff & data$ratio2<lcutoff & data$ratio3 < lcutoff,]
        #
        data_final$DownUpUp<-data[data$ratio1<lcutoff & data
$ratio2>hcutoff & data$ratio3>hcutoff,]
        data_final$DownUpNA<-data[data$ratio1<lcutoff & data
$ratio2>hcutoff & data$ratio3<hcutoff & data$ratio3>lcutoff,]
        data_final$DownUpDown<-data[data$ratio1<lcutoff & data
$ratio2>hcutoff & data$ratio3<lcutoff,]
        #
        data_final$DownNAUp<-data[data$ratio1<lcutoff & data
$ratio2<hcutoff & data$ratio2>lcutoff & data$ratio3>hcutoff,]
        data_final$DownNANA<-data[data$ratio1<lcutoff & data
$ratio2<hcutoff & data$ratio2>lcutoff & data$ratio3<hcutoff & data
$ratio3>lcutoff,]
        data_final$DownNADown<-data[data$ratio1<lcutoff & data
$ratio2<hcutoff & data$ratio2>lcutoff & data$ratio3<lcutoff,]
        #
        data_final$DownDownUp<-data[data$ratio1<lcutoff & data
$ratio2<lcutoff & data$ratio3>hcutoff,]
        data_final$DownDownNA<-data[data$ratio1<lcutoff & data
$ratio2<lcutoff & data$ratio3<hcutoff &data$ratio3>lcutoff,]
        data_final$DownDownDown<-data[data$ratio1<lcutoff & data
$ratio2<lcutoff & data$ratio3<lcutoff,]

        return(data_final)
}

getGeneNames<-function(data){
        #Input cluster data from above.
        #Writes all clusters to txt files.
        for (i in 1:length(data)) {
                write.table(data[i], paste(i,"_cluster",".txt", sep =
""),sep="\t",quote = FALSE, row.names = F, col.names = T)
        }
}

##Below is a function that was not used for the final version of this
study##
geneTally<-function(filename){
        data<-read.csv(filename,header=TRUE)
        data<-unique(data)
        combined<-as.character(data$Description)
        data.strings<-strsplit(combined,"Mol")
        desc<-lapply(data.strings,'[[',1)
```

```
        df <- data.frame(matrix(unlist(desc), nrow=length(desc),
byrow=T))
        colnames(df) <- "descriptions"
        final<-count(df,"descriptions")

        return(final)
}
```
