# Supplementary material for: Microarray Analysis of Gene Expression in Saccharomyces cerevisiae kap108Δ Mutants upon Addition of Oxidative Stress
Source: G3 (Bethesda). 2016 Feb 17;6(4):1131–9. doi: 10.1534/g3.116.027011 (PMC4825647; doi:10.1534/g3.116.027011)
Supplement: Supporting Information [file supp_g3.116.027011_TableS1.docx]

Table S1. List of genes with 1.5 fold or greater difference in expression between *kap108∆* (M) and wild-type (WT) cells (p<0.05 by unpaired t-test with Bonferonni correction). Values shown are log_2_ corrected. Log2 fold change was calculated by subtracting WT from M. Bioprocesses are derived from *Saccharomyces* Genome Database (available at www.yeastgenome.org).

| **Gene Name** | **M** | **WT** | **Fold Change** | **Bioprocess** |
| --- | --- | --- | --- | --- |
| PRM2 | 1.620 | -2.196 | 3.816 | Karyogamy involved in conjugation with cellular fusion |
| FIG1 | 1.465 | -1.984 | 3.449 | Cytogamy |
| YGR109W-A | 1.107 | -1.516 | 2.622 | RNA-mediated transposition |
| STE2 | 1.052 | -1.527 | 2.580 | Pheromone-dependent signal transduction involved in conjugation with cellular fusion |
| DAL1 | 1.000 | -1.571 | 2.571 | Hydrolase activity |
| ANB1 | 0.969 | -1.191 | 2.160 | Translation factor activity |
| YIL082W-A | 1.005 | -0.967 | 1.972 | RNA mediated transposition |
| DSF1 | 0.730 | -0.866 | 1.596 | Unknown |
| RCK1 | 0.568 | -0.979 | 1.546 | Regulation of meiotic nuclear division; kinase activity |
| PRM8 | 0.800 | -0.714 | 1.514 | Pheromone response |
| YMR279C | 0.540 | -0.958 | 1.498 | Borate transmembrane transport |
| YNR073C | 0.741 | -0.660 | 1.401 | Unknown |
| SIP4 | 0.551 | -0.817 | 1.368 | Positive regulation of gluconeogenesis |
| FIG2 | 0.666 | -0.655 | 1.321 | Cell morphogenesis involved in conjugation with cellular fusion |
| FUS2 | 0.729 | -0.492 | 1.221 | Karyogamy involved in conjugation with cellular fusion |
| YGR050C | 0.518 | -0.652 | 1.171 | Unknown |
| YKR015C | 0.672 | -0.485 | 1.157 | Unknown |
| YKR041W | 0.514 | -0.604 | 1.118 | Unknown |
| VBA3 | 0.563 | -0.503 | 1.066 | Amino Acid Transport |
| AAC3 | 0.542 | -0.511 | 1.053 | ATP/ADP exchange; anaerobic respiration |
| RPP0 | 0.654 | -0.375 | 1.029 | Ribosomal large subunit assembly |
| YOR387C | 0.359 | -0.645 | 1.004 | Unknown |
| YJR146W | 0.285 | -0.637 | 0.922 | Unknown |
| YDR374C | 0.262 | -0.659 | 0.922 | Regulation of mRNA stability |
| GMC2 | 0.377 | -0.521 | 0.899 | Meiotic nuclear division |
| RPS26A | 0.586 | -0.312 | 0.897 | rRNA export from nucleus; cytoplasmic translation |
| ARG5,6 | 0.306 | -0.577 | 0.883 | Transcription regulation; arginine and ornithine biosynthetic process |
| PGI1 | 0.438 | -0.442 | 0.880 | Gluconeogenesis |
| NHP6B | 0.367 | -0.452 | 0.819 | Chromatin remodeling; transcription from RNA Pol II promoter, RNA Pol III transcriptional preinitiation complex assembly |
| SSB1 | 0.439 | -0.377 | 0.816 | Protein folding, export of ribosomal subunit from nucleus; regulation of translational fidelity; rRNA processing |
| YLR179C | 0.401 | -0.379 | 0.780 | Unknown |
| CUP1-2 | 0.435 | -0.288 | 0.722 | Detoxification of copper ion; removal of superoxide radicals; detoxification of cadmium ion |
| CUP1-1 | 0.435 | -0.286 | 0.721 | Detoxification of copper ion; removal of superoxide radicals; detoxification of cadmium ion |
| TIF2 | 0.356 | -0.322 | 0.678 | Regulation of translational initiation |
| MEK1 | 0.354 | -0.315 | 0.669 | Protein phosphorylation; meiotic nuclear division |
| CPA2 | 0.352 | -0.299 | 0.651 | Arginine biosynthesis |
| SIP18 | 0.353 | -0.276 | 0.629 | Cellular response to water deprivation |
| PUG1 | 0.305 | -0.307 | 0.612 | Heme transport |
| LAC1 | 0.210 | -0.394 | 0.604 | Ceramide biosynthesis; replicative cell aging |
| YGR273C | -0.302 | 0.289 | -0.591 | Unknown |
| SAM3 | -0.277 | 0.329 | -0.605 | Amino acid transport |
| YCL001W-B | -0.344 | 0.278 | -0.622 | Unknown |
| ECM11 | -0.331 | 0.307 | -0.638 | Reciprocal meiotic recombination; synaptonemal complex organization |
| ZEO1 | -0.391 | 0.252 | -0.643 | Cell wall organization |
| ATG34 | -0.301 | 0.348 | -0.649 | Protein localization to vacuole involved in autophagy |
| ADR1 | -0.337 | 0.336 | -0.673 | Activation of genes involved in peroxisome organization and ethanol, glycerol, and fatty acid utilization |
| ZIP2 | -0.308 | 0.373 | -0.680 | Synapsis |
| RRT8 | -0.365 | 0.339 | -0.704 | Ascospore wall assembly |
| TRX2 | -0.382 | 0.322 | -0.704 | Golgi vesicle transport; vacuole organization |
| HXT5 | -0.300 | 0.425 | -0.724 | Hexose transport; glucose transport |
| YOR365C | -0.316 | 0.417 | -0.733 | Unknown |
| GPX2 | -0.376 | 0.358 | -0.735 | Cellular response to oxidative stress |
| GSC2 | -0.184 | 0.558 | -0.742 | Ascospore wall assembly |
| YHR214W | -0.522 | 0.265 | -0.787 | Unknown |
| ROX1 | -0.412 | 0.416 | -0.828 | Negative regulation of transcription from RNA Pol II promoter; represses expression of hypoxia-induced genes in presence of oxygen; response to osmotic stress |
| YHB1 | -0.454 | 0.388 | -0.842 | Cellular response to oxidative stress |
| CRC1 | -0.558 | 0.287 | -0.845 | Fatty acid metabolic process |
| XYL2 | -0.431 | 0.414 | -0.846 | Xylulose biosynthetic process |
| BAT2 | -0.413 | 0.461 | -0.874 | Branched-chain amino acid biosynthetic and catabolic processes |
| YGL204C | -0.476 | 0.399 | -0.875 | Unknown |
| PRY1 | -0.364 | 0.544 | -0.908 | Sterol transport |
| DDR48 | -0.348 | 0.564 | -0.912 | DNA repair |
| CYB2 | -0.627 | 0.310 | -0.937 | Lactate metabolic process |
| HXT1 | -0.487 | 0.468 | -0.954 | Glucose transport; mannose transport |
| YBR219C | -0.669 | 0.310 | -0.979 | Unknown |
| TSA1 | -0.437 | 0.552 | -0.989 | Cellular response to DNA damage stimulus; protein folding; response to oxidative stress |
| ANS1 | -0.375 | 0.637 | -1.012 | Unknown |
| CUP9 | -0.654 | 0.440 | -1.094 | Negative regulation of oligopeptide transport by negative regulation of transcription from RNA polymerase II promoter |
| VPS73 | -0.473 | 0.653 | -1.126 | Transmembrane transport |
| INH1 | -0.734 | 0.616 | -1.350 | Negative regulation of ATPase activity |
| HXT10 | -0.771 | 0.842 | -1.613 | Hexose transport |
| YMR317W | -1.022 | 0.652 | -1.674 | Unknown |
| NCA3 | -1.293 | 0.746 | -2.039 | Mitochondrion organization |
| SXM1 | -3.987 | 2.252 | -6.239 | Nuclear transport |

Table S2 List of 511 genes filtered based on having at least a 40% change to differential gene expression in at least one of three post-oxidation time points as compared to normal growth conditions. The column headers fc_t0, fc_t10, fc_t60, and fc_t120 are representative of fold change between mutant and wild-type cells before the addition of oxidative stress, after 10 min oxidative stress, after 60 min oxidative stress, and after 60 min oxidative stress followed by 60 min without stress, respectively. Clustering was performed by categorizing genes based on how their differential expression changed between time points: a 20% increase was considered “Up,” a 20% decrease was considered “Dn,” and everything else was considered “Nc.” Bioprocesses are derived from *Saccharomyces* Genome Database (available at www.yeastgenome.org).

| **Gene_Name** | **fc_t0** | **fc_t10** | **fc_t60** | **fc_t120** | **Cluster** | **Bioprocess** |
| --- | --- | --- | --- | --- | --- | --- |
| YBL029W | 1.356 | 1.900 | 2.489 | 2.402 | UpUpNc | Unknown |
| YGL230C | 1.385 | 1.680 | 2.289 | 2.394 | UpUpNc | Unknown |
| YPL014W | 0.780 | 0.971 | 1.261 | 1.255 | UpUpNc | Unknown |
| CWC25 | 1.193 | 1.801 | 2.226 | 0.907 | UpUpDn | mRNA splicing via spliceosome |
| FLO9 | 1.135 | 1.387 | 1.831 | 1.070 | UpUpDn | Flocculation |
| IRC23 | 1.176 | 1.415 | 2.399 | 1.140 | UpUpDn | Unknown |
| MIP6 | 1.578 | 2.328 | 2.938 | 0.753 | UpUpDn | mRNA export from nucleus |
| MPC54 | 1.128 | 1.491 | 2.334 | 1.176 | UpUpDn | Ascospore wall assembly; vesicle docking |
| MTH1 | 1.416 | 2.206 | 2.857 | 1.270 | UpUpDn | Signal transduction; glucose transport |
| MUM3 | 1.404 | 2.023 | 2.558 | 1.630 | UpUpDn | Ascospore wall assembly; phospholipid biosynthetic process |
| OCA5 | 1.032 | 1.258 | 1.683 | 0.884 | UpUpDn | Unknown |
| PET117 | 0.998 | 1.205 | 1.677 | 0.862 | UpUpDn | Assembly of cytochrome c oxidase |
| RGS2 | 0.690 | 0.949 | 1.357 | 0.558 | UpUpDn | Adenylate cyclase-modulating G-protein coupled receptor signaling pathway |
| YDL183C | 0.901 | 1.140 | 1.555 | 1.218 | UpUpDn | Potassium ion transport; proton transport |
| YIR042C | 0.724 | 1.080 | 1.361 | 0.828 | UpUpDn | Unknown |
| YJR012C | 1.028 | 1.244 | 1.656 | 1.216 | UpUpDn | Unknown |
| YNR071C | 1.315 | 1.678 | 2.269 | 1.701 | UpUpDn | Unknown |
| YOR152C | 0.812 | 1.028 | 1.603 | 0.949 | UpUpDn | Reticulophagy |
| DIP5 | 0.732 | 0.895 | 0.809 | 1.125 | UpNcUp | Amino acid transport |
| FMP48 | 0.601 | 0.729 | 0.658 | 1.004 | UpNcUp | Protein phosphorylation |
| GAS4 | 0.749 | 0.932 | 0.895 | 1.782 | UpNcUp | Ascospore wall assembly |
| GSY1 | 1.024 | 1.464 | 1.428 | 1.752 | UpNcUp | Glycogen biosynthetic process |
| HMS1 | 0.648 | 0.824 | 0.699 | 1.008 | UpNcUp | Pseudohyphal growth |
| HXT14 | 0.677 | 0.886 | 0.735 | 1.669 | UpNcUp | Hexose transport |
| HXT5 | 0.605 | 0.900 | 0.732 | 0.925 | UpNcUp | Hexose transport; glucose transport |
| INH1 | 0.392 | 0.516 | 0.506 | 0.895 | UpNcUp | Negative regulation of ATPase activity |
| LEE1 | 0.682 | 0.899 | 0.730 | 1.541 | UpNcUp | Unknown |
| MFA2 | 0.761 | 0.973 | 0.819 | 1.559 | UpNcUp | Pheromone-dependent signal transduction involved in conjugation with cellular fusion |
| MGA1 | 0.674 | 0.879 | 0.806 | 1.453 | UpNcUp | Filamentous growth |
| NDE2 | 0.625 | 0.757 | 0.735 | 1.009 | UpNcUp | NADH oxidation; glycolytic fermentation to ethanol |
| PCH2 | 0.701 | 0.902 | 0.732 | 1.105 | UpNcUp | Reciprocal meiotic recombination; meiotic DNA double-strand break formation; meiotic recombination checkpoint |
| SPS1 | 0.994 | 1.218 | 1.028 | 1.577 | UpNcUp | Protein phosphorylation; ascospore wall assembly; ascospore wall formation |
| ULI1 | 0.938 | 1.329 | 1.178 | 1.464 | UpNcUp | ER unfolded protein response |
| YDL199C | 0.600 | 0.853 | 0.839 | 1.119 | UpNcUp | Transmembrane transport |
| YGL015C | 0.807 | 0.995 | 0.849 | 1.535 | UpNcUp | Unknown |
| YHR210C | 0.595 | 0.807 | 0.768 | 1.676 | UpNcUp | Unknown |
| YIL054W | 0.686 | 0.873 | 0.732 | 1.379 | UpNcUp | Unknown |
| YMR206W | 0.682 | 0.904 | 0.733 | 1.489 | UpNcUp | Unknown |
| YNL146W | 0.690 | 0.848 | 0.735 | 2.532 | UpNcUp | Unknown |
| ECM27 | 0.725 | 0.889 | 0.964 | 1.038 | UpNcNc | Unknown |
| GAL3 | 0.721 | 0.875 | 1.011 | 0.832 | UpNcNc | Positive regulation of transcription from RNA Pol II promoter by galactose; maintenance of protein location |
| MRPL25 | 0.665 | 0.948 | 1.050 | 0.843 | UpNcNc | Mitochondrial translation |
| POX1 | 0.615 | 0.760 | 0.736 | 0.867 | UpNcNc | Fatty acid beta-oxidation using acyl-CoA oxidase |
| SPO16 | 0.571 | 0.710 | 0.811 | 0.826 | UpNcNc | Ascospore formation; protein sumoylation; meiotic recombination; synaptonemal complex assembly |
| SPS4 | 0.708 | 0.868 | 0.939 | 1.078 | UpNcNc | Ascospore Formation |
| YNL018C | 0.389 | 0.627 | 0.666 | 0.616 | UpNcNc | Unknown |
| ZIP2 | 0.624 | 0.896 | 0.731 | 0.673 | UpNcNc | Synapsis |
| ARG3 | 1.689 | 2.081 | 2.173 | 0.976 | UpNcDn | Arginine biosynthetic process |
| CMR3 | 1.346 | 2.111 | 2.428 | 1.279 | UpNcDn | Unknown |
| FIG1 | 10.918 | 13.152 | 12.760 | 3.629 | UpNcDn | Cytogamy |
| FIG2 | 2.498 | 3.159 | 2.692 | 1.074 | UpNcDn | Cell morphogenesis involved in conjugation with cellular fusion |
| FMP33 | 1.128 | 1.619 | 1.904 | 1.091 | UpNcDn | Unknown |
| HUG1 | 2.184 | 2.665 | 2.664 | 1.286 | UpNcDn | Cellular response to DNA damage stimulus |
| HXT4 | 1.230 | 1.657 | 1.816 | 1.336 | UpNcDn | Hexose transport |
| IRC4 | 0.818 | 1.107 | 1.251 | 0.913 | UpNcDn | Mitotic recombination |
| MRPL33 | 0.853 | 1.074 | 1.239 | 0.898 | UpNcDn | Mitochondrial translation |
| MSH4 | 1.255 | 2.328 | 2.579 | 1.615 | UpNcDn | Reciprocal meiotic recombination |
| SMD2 | 1.054 | 1.371 | 1.604 | 0.938 | UpNcDn | mRNA splicing via spliceosome |
| STE2 | 5.978 | 11.771 | 10.234 | 1.833 | UpNcDn | Pheromone-dependent signal transduction involved in conjugation with cellular fusion |
| STR3 | 1.534 | 2.762 | 2.878 | 0.539 | UpNcDn | Methionine biosynthetic process; transsulfuration |
| YDL009C | 0.991 | 1.192 | 1.427 | 1.015 | UpNcDn | Unknown |
| YFR012W-A | 1.933 | 3.015 | 2.689 | 1.831 | UpNcDn | Unknown |
| YGL010W | 0.921 | 1.288 | 1.503 | 1.016 | UpNcDn | Sphingoid catabolic process |
| YOR012W | 1.540 | 2.192 | 2.051 | 1.305 | UpNcDn | Unknown |
| ADH7 | 0.751 | 1.199 | 0.781 | 1.488 | UpDnUp | Alcohol metabolic process |
| GLO4 | 0.600 | 0.851 | 0.668 | 1.108 | UpDnUp | Methylglyoxal catabolic process to D-lactate via S-lactoyl-glutathione |
| GPX1 | 0.695 | 0.895 | 0.648 | 1.192 | UpDnUp | Peroxisome organization; cellular response to oxidative stress |
| HSP12 | 0.170 | 0.229 | 0.146 | 0.719 | UpDnUp | Cellular response to oxidative stress, osmotic stress, and heat; cell adhesion; plasma membrane organization |
| MOH1 | 0.843 | 1.102 | 0.725 | 1.221 | UpDnUp | Unknown |
| MRK1 | 1.289 | 1.937 | 1.311 | 2.086 | UpDnUp | Protein phosphorylation; cellular response to hear and salt stress; regulation of protein catabolic process |
| MUP3 | 1.017 | 1.300 | 0.459 | 1.211 | UpDnUp | Methionine import |
| RGI1 | 1.004 | 1.207 | 0.627 | 2.299 | UpDnUp | Energy reserve metabolic process |
| RTC3 | 0.754 | 0.998 | 0.555 | 1.369 | UpDnUp | RNA metabolic process |
| SOL4 | 0.735 | 0.984 | 0.367 | 1.042 | UpDnUp | Pentose-phosphate shunt |
| TMA10 | 0.714 | 1.069 | 0.303 | 1.786 | UpDnUp | Unknown |
| TPP1 | 0.868 | 1.478 | 1.033 | 1.254 | UpDnUp | Double-strand break repair |
| XBP1 | 1.336 | 1.642 | 0.786 | 1.302 | UpDnUp | Cellular response to oxidative stress; negative regulation of transcription from RNA Pol II promoter; positive regulation of histone deacetylation |
| YCL001W-A | 0.861 | 1.119 | 0.800 | 2.119 | UpDnUp | Unknown |
| YDR018C | 0.741 | 1.015 | 0.727 | 2.139 | UpDnUp | Phospholipid biosynthetic process |
| YEL057C | 0.678 | 1.056 | 0.730 | 2.011 | UpDnUp | Unknown |
| YGL258W-A | 0.735 | 0.916 | 0.732 | 1.936 | UpDnUp | Unknown |
| YMR085W | 1.122 | 1.435 | 0.802 | 1.581 | UpDnUp | Unknown |
| YOL047C | 1.558 | 2.459 | 0.756 | 1.538 | UpDnUp | Ascospore wall assembly |
| ATP8 | 0.650 | 1.119 | 0.608 | 0.686 | UpDnNc | ATP synthesis coupled proton transport |
| RRT5 | 1.012 | 1.439 | 1.053 | 0.944 | UpDnNc | Unknown |
| YCL001W-B | 0.650 | 0.922 | 0.732 | 0.860 | UpDnNc | Unknown |
| YNR064C | 0.802 | 1.147 | 0.740 | 0.621 | UpDnNc | Response to toxic substance |
| ASG7 | 3.914 | 5.994 | 4.536 | 3.539 | UpDnDn | Conjugation with cellular fusion |
| BAR1 | 1.940 | 2.693 | 1.649 | 0.844 | UpDnDn | Adaptation of signaling pathway by response to pheromone involved in conjugation with cellular fusion; peptide catabolic process |
| MDJ2 | 1.028 | 1.423 | 0.882 | 0.557 | UpDnDn | Protein import into mitochondrial matrix |
| SRD1 | 1.745 | 2.466 | 1.513 | 0.858 | UpDnDn | rRNA processing |
| YNL162W-A | 0.776 | 1.544 | 0.938 | 0.465 | UpDnDn | Unknown |
| CSR2 | 0.896 | 0.869 | 1.092 | 1.425 | NcUpUp | Regulation of transcription from RNA Pol II promoter; cell wall organization; ubiquitin-dependent endocytosis |
| FRE7 | 0.536 | 0.517 | 0.712 | 1.095 | NcUpUp | Iron ion transport; copper ion import |
| GAL4 | 0.830 | 0.962 | 1.443 | 1.827 | NcUpUp | Positive regulation f transcription from RNA Pol II promoter by galactose; galactose metabolic process |
| MLH2 | 0.798 | 0.762 | 1.174 | 1.560 | NcUpUp | Mismatch repair; reciprocal meiotic recombination |
| THI2 | 0.831 | 0.846 | 1.088 | 1.652 | NcUpUp | Positive regulation of thiamine biosynthetic process; positive regulation of transcription from RNA Pol II promoter |
| YAK1 | 0.981 | 1.143 | 1.429 | 1.762 | NcUpUp | Protein phosphorylation |
| YCL068C | 0.713 | 0.753 | 0.909 | 1.154 | NcUpUp | Unknown |
| YHR022C | 0.635 | 0.551 | 0.733 | 1.070 | NcUpUp | Unknown |
| DAN1 | 0.774 | 0.860 | 1.170 | 1.393 | NcUpNc | Sterol transport |
| MAL11 | 1.134 | 1.113 | 1.651 | 1.541 | NcUpNc | Maltose transport; trehalose transport; disaccharide catabolic process |
| MSA2 | 0.951 | 1.034 | 1.426 | 1.425 | NcUpNc | Regulation of transcription involved in G1/S transition of mitotic cell cycle |
| RRI2 | 1.240 | 1.387 | 1.843 | 1.550 | NcUpNc | Cullin deneddylation; adaption of signaling pathway by response to pheromone involved in conjugation with cellular fusion |
| RTA1 | 1.418 | 1.658 | 2.231 | 2.200 | NcUpNc | Unknown |
| TEP1 | 1.009 | 1.110 | 1.616 | 1.339 | NcUpNc | Ascospore wall assembly; phosphatidylinositol dephosphorylation |
| WSC4 | 0.707 | 0.810 | 1.049 | 1.236 | NcUpNc | Protein targeting to ER; response to heat |
| YBR184W | 1.504 | 1.669 | 2.296 | 1.910 | NcUpNc | Unknown |
| YGR016W | 1.089 | 1.191 | 1.641 | 1.373 | NcUpNc | Unknown |
| YGR018C | 0.889 | 0.798 | 1.271 | 1.325 | NcUpNc | Unknown |
| YLR446W | 1.123 | 1.159 | 1.610 | 1.299 | NcUpNc | Unknown |
| YNL092W | 0.636 | 0.676 | 0.887 | 1.015 | NcUpNc | Protein methylation |
| AEP3 | 0.991 | 1.171 | 1.564 | 1.112 | NcUpDn | mRNA metabolic process; mitochondrial translation initiation |
| BNA2 | 1.428 | 1.674 | 2.611 | 1.463 | NcUpDn | Tryptophan catabolic process to kynurenine; 'de novo' NAD biosynthetic process from tryptophan |
| CDC6 | 1.166 | 1.187 | 2.020 | 0.768 | NcUpDn | G1/S transition of mitotic cell cycle; pre-replicative complex assembly involved in nuclear cell cycle DNA replication; regulation of chromatin silencing at telomere |
| CLD1 | 0.960 | 0.991 | 1.368 | 0.902 | NcUpDn | Cardiolipin metabolic process; cardiolipin acyl chain remodeling |
| COS111 | 1.499 | 1.620 | 2.494 | 1.255 | NcUpDn | Signal transduction |
| MSA1 | 1.144 | 1.066 | 1.655 | 1.071 | NcUpDn | G1/S transition of mitotic cell cycle; regulation of cell size |
| OAR1 | 1.057 | 0.899 | 1.182 | 0.574 | NcUpDn | Fatty acid metabolic process; aerobic respiration |
| POG1 | 1.103 | 1.240 | 1.589 | 0.827 | NcUpDn | Re-entry into mitotic cell cycle after pheromone arrest; regulation of transcription from RNA Pol II promoter |
| PRM2 | 14.087 | 16.255 | 30.056 | 5.850 | NcUpDn | Karyogamy involved in conjugation with cellular fusion |
| RGM1 | 1.236 | 1.370 | 1.879 | 1.021 | NcUpDn | Positive regulation of transcription from RNA Pol II promoter |
| SNA2 | 1.102 | 1.194 | 1.578 | 0.936 | NcUpDn | Unknown |
| SSK1 | 1.202 | 1.192 | 1.743 | 1.351 | NcUpDn | Activation of MAPKKK activity involved in osmosensory signaling pathway; positive regulation of actin cytoskeleton reorganization |
| THI12 | 1.340 | 1.371 | 2.257 | 1.215 | NcUpDn | Thiamine biosynthetic process |
| THI5 | 1.298 | 1.342 | 2.446 | 1.238 | NcUpDn | Thiamine biosynthetic process |
| YDR124W | 1.617 | 1.909 | 2.859 | 1.412 | NcUpDn | Unknown |
| YHR214C-D | 0.494 | 0.440 | 0.698 | 0.481 | NcUpDn | Unknown |
| AAD3 | 1.319 | 1.269 | 1.460 | 2.495 | NcNcUp | Cellular aldehyde metabolic process |
| AAP1 | 0.775 | 0.751 | 0.772 | 1.173 | NcNcUp | Glycogen metabolic process; proteolysis |
| ADE17 | 0.517 | 0.520 | 0.527 | 0.874 | NcNcUp | Purine nucleotide biosynthetic process; 'de novo' IMP biosynthetic process |
| ADH5 | 1.550 | 1.846 | 1.931 | 2.905 | NcNcUp | NADH oxidation; amino acid catabolic process to alcohol via Ehrlich pathway; ethanol biosynthetic process involved in glucose fermentation to ethanol |
| AHP1 | 0.635 | 0.604 | 0.579 | 1.479 | NcNcUp | Response to oxidative stress; response to metal ion; cell redox homeostasis |
| AIM17 | 0.929 | 0.921 | 0.763 | 1.311 | NcNcUp | Mitochondrion organization |
| AIM39 | 0.876 | 0.901 | 0.949 | 1.240 | NcNcUp | Unknown |
| AIM44 | 0.935 | 0.991 | 1.040 | 1.456 | NcNcUp | Barrier septum assembly; protein localization to bud neck |
| AIM46 | 1.095 | 1.166 | 1.075 | 1.590 | NcNcUp | Unknown |
| AME1 | 1.048 | 1.091 | 1.070 | 1.533 | NcNcUp | Attachment of spindle microtubules to kinetochore; protein localization to kinetochore |
| APE1 | 0.817 | 0.783 | 0.734 | 1.502 | NcNcUp | Protein catabolic process in the vacuole |
| AQY2 | 0.942 | 0.861 | 0.889 | 1.326 | NcNcUp | Water transport |
| ARA2 | 0.813 | 0.828 | 0.858 | 1.262 | NcNcUp | Dehydro-D-arabinono-1,4-lactone biosynthetic process |
| ARN1 | 0.840 | 0.775 | 0.814 | 1.220 | NcNcUp | Siderophore transport |
| ARN2 | 0.668 | 0.661 | 0.641 | 1.113 | NcNcUp | Siderophore transport; cellular iron ion homeostasis |
| ARO10 | 0.558 | 0.572 | 0.525 | 1.100 | NcNcUp | Aromatic amino acid family catabolic process to alcohol via Ehrlich pathway |
| ARR2 | 1.083 | 1.214 | 1.280 | 1.905 | NcNcUp | Response to arsenic-containing substance |
| ASE1 | 1.064 | 0.976 | 0.977 | 2.029 | NcNcUp | Mitotic spindle elongation; spindle pole body separation; cell separation after cytokinesis; microtubule bundle formation; mitotic spindle stabilization; spindle midzone assembly |
| ASH1 | 1.130 | 1.198 | 1.183 | 1.740 | NcNcUp | Chromatin organization; positive regulation of pseudohyphal growth; negative regulation of mating type switching; negative regulation of G1/S transition of mitotic cell cycle |
| ASI3 | 1.049 | 1.011 | 1.040 | 1.551 | NcNcUp | Ubiquitin-dependent protein catabolic process; cellular response to amino acid stimulus |
| ATG20 | 0.869 | 0.888 | 0.923 | 1.328 | NcNcUp | Mitophagy; macroautophagy; CVT pathway; early endosome to Golgi transport |
| ATG34 | 0.638 | 0.731 | 0.639 | 1.025 | NcNcUp | Protein localization to vacuole involved in autophagy |
| ATO2 | 0.634 | 0.708 | 0.806 | 1.072 | NcNcUp | Ammonium transport; nitrogen utilization |
| ATR1 | 0.970 | 0.976 | 0.924 | 2.174 | NcNcUp | Drug transmembrane transport; borate transport |
| BAG7 | 0.808 | 0.902 | 0.736 | 2.495 | NcNcUp | Negative regulation of Rho protein signal transduction; positive regulation of GTPase activity |
| BAT2 | 0.546 | 0.544 | 0.522 | 0.908 | NcNcUp | Branched-chain amino acid biosynthetic and catabolic processes |
| BNS1 | 0.797 | 0.815 | 0.905 | 1.122 | NcNcUp | Meiotic nuclear division |
| BXI1 | 0.810 | 0.830 | 0.800 | 1.180 | NcNcUp | Apoptotic process; calcium-mediated signaling; ER unfolded protein response |
| CHA1 | 0.974 | 1.019 | 1.047 | 1.442 | NcNcUp | L-serine catabolic process; threonine catabolic process |
| CMK1 | 0.944 | 0.946 | 1.004 | 1.330 | NcNcUp | Protein phosphorylation; signal transduction |
| CPR6 | 0.899 | 0.933 | 0.889 | 1.520 | NcNcUp | Protein folding |
| CUP9 | 0.468 | 0.481 | 0.509 | 0.917 | NcNcUp | Negative regulation of oligopeptide transport by negative regulation of transcription from RNA polymerase II promoter |
| CYB2 | 0.522 | 0.433 | 0.416 | 0.824 | NcNcUp | Lactate metabolic process |
| DAK2 | 0.268 | 0.254 | 0.236 | 1.109 | NcNcUp | Glycerol catabolic process; cellular response to toxic substance |
| DAN4 | 0.820 | 0.842 | 0.752 | 1.178 | NcNcUp | Unknown |
| DDR48 | 0.531 | 0.555 | 0.492 | 1.115 | NcNcUp | DNA repair |
| DIG2 | 0.981 | 1.004 | 0.940 | 1.385 | NcNcUp | Negative regulation of transcription from RNA Pol II promoter by pheromones; negative regulation of invasive growth in response to glucose limitation; negative regulation of pseudohyphal growth |
| DOA4 | 0.974 | 0.855 | 0.845 | 1.434 | NcNcUp | Endocytosis; ubiquitin-dependent protein catabolic process; intralumenal vesicle formation; regulation of DNA replication; free ubiquitin chain depolymerization |
| DOT6 | 0.732 | 0.773 | 0.764 | 1.067 | NcNcUp | Chromatin silencing at rDNA and telomere; tRNA gene-mediated silencing; regulation of transcription from RNA Pol II promoter |
| DRE2 | 1.087 | 1.087 | 1.028 | 1.589 | NcNcUp | Iron-sulfur cluster assembly |
| ECM12 | 0.632 | 0.573 | 0.574 | 0.953 | NcNcUp | Unknown |
| ECM18 | 1.085 | 0.915 | 0.955 | 2.026 | NcNcUp | Unknown |
| ECM38 | 0.830 | 0.788 | 0.757 | 1.163 | NcNcUp | Glutathione catabolic xenobiotic metabolic process |
| EEB1 | 0.724 | 0.850 | 0.820 | 1.324 | NcNcUp | Medium-chain fatty acid biosynthetic process |
| EGT2 | 1.032 | 0.980 | 1.038 | 1.467 | NcNcUp | Cell separation after cytokinesis |
| EHT1 | 0.639 | 0.584 | 0.600 | 1.005 | NcNcUp | Cellular lipid metabolic process; medium-chain fatty acid biosynthetic process |
| FAT3 | 0.921 | 0.913 | 0.953 | 1.505 | NcNcUp | Long-chain fatty acid transport |
| FES1 | 1.083 | 1.166 | 0.943 | 1.760 | NcNcUp | Cytoplasmic translation; cytoplasm-associated proteasomal ubiquitin-dependent protein catabolic process |
| FET4 | 1.229 | 1.188 | 1.326 | 1.808 | NcNcUp | Zinc II ion transport; copper ion transport |
| FIT1 | 1.262 | 1.230 | 1.241 | 2.092 | NcNcUp | Siderophore transport |
| FMP23 | 0.832 | 0.967 | 0.857 | 2.604 | NcNcUp | Unknown |
| FMP43 | 0.668 | 0.782 | 0.808 | 1.142 | NcNcUp | Mitochondrial pyruvate transport |
| FRE1 | 0.691 | 0.719 | 0.787 | 1.092 | NcNcUp | Iron ion transport; copper ion import |
| FRE3 | 0.856 | 0.839 | 0.853 | 1.239 | NcNcUp | Cellular iron ion homeostasis |
| FRE4 | 1.129 | 1.118 | 1.071 | 7.195 | NcNcUp | Siderophore transport |
| FUN19 | 1.068 | 1.051 | 1.026 | 1.600 | NcNcUp | Unknown |
| GEX1 | 0.597 | 0.511 | 0.496 | 0.938 | NcNcUp | Glutathione transmembrane transport |
| GGA1 | 0.787 | 0.805 | 0.807 | 1.396 | NcNcUp | Golgi to endosome transport |
| GIC2 | 0.835 | 0.863 | 0.933 | 1.170 | NcNcUp | Regulation of exit from mitosis; establishment of cell polarity; septin ring organization; positive regulation of formin-nucleated actin cable assembly |
| GIP1 | 1.004 | 1.037 | 0.901 | 2.362 | NcNcUp | Ascospore wall assembly; regulation of phosphoprotein phosphatase activity |
| GIS1 | 0.841 | 0.791 | 0.784 | 1.215 | NcNcUp | Histone modification; maintenance of stationary phase in response to starvation; regulation of phospholipid biosynthetic process; chronological cell aging |
| GLC3 | 0.803 | 0.891 | 0.724 | 1.277 | NcNcUp | Glycogen biosynthetic process |
| GPX2 | 0.601 | 0.616 | 0.517 | 1.565 | NcNcUp | Cellular response to oxidative stress |
| GRE2 | 0.768 | 0.767 | 0.682 | 1.659 | NcNcUp | Ergosterol metabolic process; filamentous growth |
| GRX2 | 0.792 | 0.880 | 0.766 | 1.219 | NcNcUp | Glutathione metabolic process; cellular response to oxidative stress |
| GSH1 | 0.828 | 0.794 | 0.754 | 1.513 | NcNcUp | Glutathione biosynthetic process; response to hydrogen peroxide; response to cadmium ion |
| GSM1 | 0.763 | 0.716 | 0.805 | 1.112 | NcNcUp | Oxidative phosphorylation |
| GTO3 | 0.915 | 0.738 | 0.839 | 1.779 | NcNcUp | Glutathione metabolic process |
| GTT1 | 0.731 | 0.722 | 0.705 | 1.076 | NcNcUp | Glutathione metabolic process |
| HHT2 | 0.790 | 0.853 | 0.901 | 1.177 | NcNcUp | Chromatin organization; mitotic spindle assembly checkpoint; rRNA transcription; sexual sporulation resulting in formation of a cellular spore; global genome nucleotide-excision repair |
| HMX1 | 1.081 | 1.155 | 1.082 | 1.654 | NcNcUp | Cellular iron ion homeostasis; response to oxidative stress; heme catabolic process |
| HOF1 | 1.032 | 1.047 | 1.013 | 1.548 | NcNcUp | Mitotic cytokinesis; barrier septum assembly; negative regulation of formin-nucleated actin cable assembly |
| HRD1 | 0.832 | 0.871 | 0.983 | 1.191 | NcNcUp | ER-associated ubiquitin-dependent protein catabolic process; ER unfolded protein response; cell wall organization; retrograde protein transport |
| HRK1 | 0.916 | 0.898 | 0.889 | 1.310 | NcNcUp | Cellular ion homeostasis |
| HTA2 | 0.853 | 0.888 | 0.921 | 1.302 | NcNcUp | DNA repair; chromatin organization |
| HTB2 | 0.716 | 0.841 | 0.833 | 1.254 | NcNcUp | Chromatin organization |
| HXK1 | 0.714 | 0.722 | 0.664 | 1.090 | NcNcUp | Fructose, glucose, and mannose metabolic processes; fructose and glucose import |
| HXT10 | 0.327 | 0.327 | 0.345 | 1.226 | NcNcUp | Hexose transport |
| IBA57 | 0.696 | 0.813 | 0.784 | 1.284 | NcNcUp | Iron-sulfur cluster assembly |
| ICY1 | 0.598 | 0.694 | 0.649 | 0.910 | NcNcUp | Unknown |
| IDH2 | 0.736 | 0.769 | 0.794 | 1.062 | NcNcUp | TCA cycle; glutamate biosynthetic process |
| IGD1 | 1.028 | 1.064 | 0.990 | 1.672 | NcNcUp | Negative regulation of glycogen catabolic process |
| IKS1 | 0.932 | 0.885 | 0.831 | 1.315 | NcNcUp | Kinase activity |
| IRC18 | 0.720 | 0.835 | 0.747 | 1.323 | NcNcUp | Ascospore wall assembly |
| IRC8 | 0.898 | 0.885 | 0.978 | 1.452 | NcNcUp | Mitotic recombination |
| ISF1 | 0.654 | 0.742 | 0.727 | 1.903 | NcNcUp | Aerobic respiration |
| ISU2 | 1.052 | 0.931 | 0.911 | 2.268 | NcNcUp | tRNA wobble uridine modification; cellular iron ion homeostasis; iron-sulfur cluster assembly |
| KEL2 | 0.711 | 0.658 | 0.545 | 1.124 | NcNcUp | Conjugation with cellular fusion; negative regulation of exit from mitosis; regulation of cytokinesis; regulation of formin-nucleated actin cable assembly |
| KIP2 | 1.112 | 0.994 | 1.062 | 1.636 | NcNcUp | Negative regulation of microtubule depolymerization; nuclear migration along microtubule |
| LSB6 | 0.848 | 0.798 | 0.854 | 1.277 | NcNcUp | Phosphatidylinositol phosphorylation |
| LST8 | 0.843 | 0.913 | 0.860 | 1.381 | NcNcUp | Regulation of cell growth; transport; signal transduction; establishment or maintenance of actin cytoskeleton polarity |
| LYS20 | 0.566 | 0.548 | 0.545 | 0.799 | NcNcUp | Histone displacement; DNA repair; lysine biosynthetic process via aminoadipic acid |
| MAL31 | 1.149 | 1.167 | 1.098 | 1.678 | NcNcUp | Alpha-glucoside transport |
| MCT1 | 0.805 | 0.813 | 0.807 | 1.147 | NcNcUp | Fatty acid metabolic process |
| MDG1 | 0.928 | 1.019 | 0.862 | 1.412 | NcNcUp | Pheromone-dependent signal transduction involved in conjugation with cellular fusion |
| MEK1 | 1.590 | 1.542 | 1.330 | 2.516 | NcNcUp | Protein phosphorylation; meiotic nuclear division |
| MF(ALPHA)2 | 1.151 | 1.308 | 1.212 | 1.652 | NcNcUp | Pheromone-dependent signal transduction involved in conjugation with cellular fusion |
| MFM1 | 0.737 | 0.797 | 0.737 | 1.084 | NcNcUp | Mitochondrial magnesium ion transport |
| MHO1 | 0.866 | 0.797 | 0.696 | 1.345 | NcNcUp | Unknown |
| MLS1 | 0.758 | 0.799 | 0.776 | 1.666 | NcNcUp | Glyoxylate cycle |
| MMT1 | 1.117 | 1.119 | 1.059 | 1.714 | NcNcUp | Cellular iron ion homeostasis |
| MMT2 | 0.947 | 0.923 | 0.932 | 1.366 | NcNcUp | Cellular iron ion homeostasis |
| MRL1 | 0.943 | 0.961 | 0.999 | 1.329 | NcNcUp | Vacuolar transport |
| MRS4 | 0.758 | 0.721 | 0.646 | 1.715 | NcNcUp | Mitochondrial iron ion transport |
| NCE102 | 0.580 | 0.682 | 0.699 | 0.944 | NcNcUp | Negative regulation of protein phosphorylation; plasma membrane organization; protein secretion; eisosome assembly; establishment of protein localization to plasma membrane |
| NDT80 | 0.574 | 0.628 | 0.551 | 0.917 | NcNcUp | Meiotic nuclear division; positive regulation of transcription from RNA Pol II promoter |
| NMA2 | 0.828 | 0.755 | 0.760 | 1.349 | NcNcUp | NAD biosynthetic process |
| NRG2 | 0.808 | 0.881 | 0.879 | 1.258 | NcNcUp | Negative regulation of transcription from RNA Pol II promoter by glucose; pseudohyphal growth; negative regulation of invasive growth in response to glucose limitation; negative regulation of cellular hyperosmotic salinity response |
| OCH1 | 0.693 | 0.651 | 0.640 | 1.052 | NcNcUp | Protein N-linked glycosylation |
| OLE1 | 0.881 | 0.940 | 0.936 | 1.521 | NcNcUp | Mitochondrion inheritance; unsaturated fatty acid biosynthetic process |
| OPI10 | 0.941 | 0.959 | 0.786 | 1.470 | NcNcUp | Inositol metabolic process |
| ORM2 | 0.844 | 0.910 | 0.856 | 1.318 | NcNcUp | Response to unfolded protein; negative regulation of sphingolipid biosynthetic process |
| PAU15 | 1.054 | 0.995 | 0.991 | 1.574 | NcNcUp | Unknown |
| PAU17 | 0.861 | 0.835 | 0.795 | 1.236 | NcNcUp | Unknown |
| PAU19 | 1.079 | 1.030 | 1.038 | 1.633 | NcNcUp | Unknown |
| PAU21 | 1.083 | 1.010 | 1.038 | 1.566 | NcNcUp | Unknown |
| PAU22 | 1.077 | 1.003 | 1.012 | 1.543 | NcNcUp | Unknown |
| PAU3 | 1.090 | 0.984 | 0.987 | 1.683 | NcNcUp | Unknown |
| PCL7 | 0.739 | 0.779 | 0.750 | 1.137 | NcNcUp | Regulation of cyclin-dependent protein serine/threonine kinase activity; regulation of glycogen biosynthetic and catabolic processes; regulation of protein stability |
| PCL9 | 0.979 | 1.061 | 1.061 | 1.640 | NcNcUp | Regulation of transcription involved in G1/S transition of mitotic cell cycle; regulation of establishment or maintenance of cell polarity |
| PDE1 | 0.812 | 0.789 | 0.795 | 1.274 | NcNcUp | cAMP-mediated signaling |
| PDR16 | 1.004 | 1.037 | 1.023 | 1.544 | NcNcUp | Phospholipid biosynthetic process and transport; sterol biosynthetic process; response to drug; negative regulation of sexual sporulation resulting in formation of a cellular spore |
| PEP12 | 0.830 | 0.929 | 0.766 | 1.308 | NcNcUp | Vacuole inheritance; Golgi to vacuole transport |
| PHD1 | 0.767 | 0.822 | 0.784 | 1.472 | NcNcUp | Positive regulation of pseudohyphal growth |
| PHO2 | 0.867 | 0.874 | 0.859 | 1.352 | NcNcUp | Histidine biosynthetic process; chromatin remodeling; purine nucleobase biosynthetic process |
| PHO5 | 0.598 | 0.586 | 0.602 | 0.954 | NcNcUp | Phosphate-containing compound metabolic process; cellular response to phosphate starvation |
| PIC2 | 0.893 | 0.883 | 0.843 | 1.404 | NcNcUp | Cellular copper ion homeostasis; copper ion transmembrane transport; phosphate ion transmembrane transport |
| PIG2 | 1.002 | 1.011 | 0.961 | 1.831 | NcNcUp | Regulation of glycogen biosynthetic process |
| PIR1 | 0.747 | 0.741 | 0.821 | 1.357 | NcNcUp | Intracellular protein transport; cell wall organization |
| PIR3 | 1.416 | 1.384 | 1.327 | 2.064 | NcNcUp | Cell wall organization |
| PNC1 | 0.716 | 0.853 | 0.696 | 1.251 | NcNcUp | Chromatin silencing at rDNA and telomere; replicative cell aging; nicotinate nucleotide salvage |
| POR1 | 0.667 | 0.693 | 0.684 | 1.024 | NcNcUp | Ion transport; Apoptotic process; mitochondrion organization; cell redox homeostasis; DNA transport |
| PPM1 | 0.657 | 0.700 | 0.661 | 1.085 | NcNcUp | C-terminal protein methylation; regulation of autophagy; cellular protein complex assembly |
| PRR2 | 1.175 | 1.049 | 1.239 | 2.064 | NcNcUp | Negative regulation of conjugation with cellular fusion |
| PST1 | 0.828 | 0.855 | 0.850 | 1.475 | NcNcUp | Cell wall organization |
| PTR2 | 0.269 | 0.243 | 0.218 | 0.423 | NcNcUp | Peptide transport |
| RDS1 | 0.981 | 0.923 | 0.910 | 1.457 | NcNcUp | Response to xenobiotic stimulus |
| REC104 | 1.107 | 0.968 | 1.023 | 1.590 | NcNcUp | Reciprocal meiotic recombination; meiotic DNA double-strand break formation |
| REE1 | 0.663 | 0.692 | 0.677 | 1.098 | NcNcUp | Regulation of Enolase |
| REG2 | 0.648 | 0.694 | 0.665 | 0.926 | NcNcUp | Negative regulation of transcription from RNA Pol II promoter |
| RGI2 | 1.439 | 1.315 | 1.333 | 2.233 | NcNcUp | Energy reserve metabolic process |
| RHO4 | 0.640 | 0.719 | 0.753 | 0.971 | NcNcUp | Maintenance of cell polarity; positive regulation of formin-nucleated actin cable assembly |
| RIB3 | 1.135 | 1.139 | 1.136 | 1.652 | NcNcUp | Aerobic respiration; riboflavin biosynthetic process |
| RIM11 | 0.895 | 0.891 | 0.902 | 1.355 | NcNcUp | Protein phosphorylation; proteolysis; ascospore formation; cellular response to heat and salt stress |
| RIM8 | 1.109 | 0.943 | 0.901 | 2.150 | NcNcUp | Invasive growth in response to glucose limitation; meiotic nuclear division; protein processing; ubiquitin-dependent endocytosis |
| ROX1 | 0.563 | 0.455 | 0.434 | 1.342 | NcNcUp | Negative regulation of transcription from RNA Pol II promoter; represses expression of hypoxia-induced genes in presence of oxygen; response to osmotic stress |
| RPN4 | 1.100 | 1.172 | 1.039 | 1.716 | NcNcUp | Regulation of DNA repair; positive regulation of transcription from RNA Pol II promoter in response to stress; positive regulation of proteasomal ubiquitin-dependent protein catabolic process |
| RRT12 | 0.844 | 0.813 | 0.803 | 1.630 | NcNcUp | Ascospore wall assembly |
| RRT6 | 0.635 | 0.629 | 0.652 | 1.120 | NcNcUp | Unknown |
| RRT8 | 0.614 | 0.670 | 0.630 | 1.121 | NcNcUp | Ascospore wall assembly |
| RSF1 | 0.900 | 1.017 | 1.078 | 1.317 | NcNcUp | aerobic respiration; calcium mediated signaling; mitochondrion organization |
| RTC2 | 0.802 | 0.648 | 0.730 | 1.494 | NcNcUp | Basic amino acid transmembrane export from vacuole |
| RTK1 | 0.838 | 0.815 | 0.752 | 1.451 | NcNcUp | Kinase activity; transferase activity |
| SAE2 | 1.118 | 1.264 | 1.038 | 1.625 | NcNcUp | Cellular response to DNA damage stimulus; nuclease activity; telomere organization |
| SDS22 | 0.818 | 0.818 | 0.814 | 1.167 | NcNcUp | Chromosome segregation; maintenance of protein location in nucleus |
| SDS24 | 0.781 | 0.762 | 0.634 | 1.251 | NcNcUp | Cell separation after cytokinesis; endocytosis |
| SER3 | 1.012 | 1.054 | 1.074 | 1.643 | NcNcUp | Serine family amino acid biosynthetic process |
| SET4 | 0.504 | 0.437 | 0.485 | 0.878 | NcNcUp | Chromatin organization |
| SFA1 | 1.131 | 1.088 | 1.043 | 1.606 | NcNcUp | Amino acid catabolic process to alcohol via Ehrlich pathway; furaldehyde metabolic process; formaldehyde catabolic process |
| SHH4 | 0.826 | 0.867 | 0.755 | 1.349 | NcNcUp | Mitochondrial electron transport |
| SIS1 | 0.882 | 0.947 | 0.791 | 1.650 | NcNcUp | Translational initiation; protein folding; tRNA import into nucleus; misfolded protein transport |
| SLM1 | 0.893 | 0.903 | 0.947 | 1.344 | NcNcUp | Cytoskeleton organization; lipid binding; regulation of cell growth |
| SNG1 | 1.042 | 0.957 | 0.844 | 1.465 | NcNcUp | Nucleobase-containing compound transport |
| SOD1 | 0.684 | 0.698 | 0.617 | 1.792 | NcNcUp | Copper and zinc ion homeostasis; superoxide metabolism; cell aging; cell wall organization; regulation of cellular respiration; regulation of transcription in response to oxidative stress |
| SPO21 | 0.768 | 0.909 | 0.912 | 1.325 | NcNcUp | Meiotic nuclear division; ascospore wall assembly |
| SPR28 | 1.135 | 1.239 | 1.367 | 2.011 | NcNcUp | Sexual sporulation resulting in formation of a cellular spore |
| SPS100 | 0.681 | 0.642 | 0.752 | 1.116 | NcNcUp | Ascospore wall assembly |
| SPS22 | 1.198 | 0.980 | 1.057 | 1.746 | NcNcUp | Ascospore wall assembly |
| SRL1 | 0.703 | 0.764 | 0.713 | 0.996 | NcNcUp | Nucleobase-containing compound metabolic process; cell wall organization |
| STB2 | 1.029 | 0.985 | 1.043 | 1.472 | NcNcUp | Unknown |
| STE18 | 1.015 | 1.190 | 1.126 | 1.424 | NcNcUp | Pheromone-dependent signal transduction involved in conjugation with cellular fusion; heterotrimeric G-protein complex cycle |
| SUR1 | 0.794 | 0.883 | 0.957 | 1.168 | NcNcUp | Sphingolipid biosynthetic process; mannosyl-inositol phosphorylceramide metabolic process |
| TDA10 | 0.790 | 0.781 | 0.786 | 1.148 | NcNcUp | Unknown |
| TDA7 | 0.990 | 1.017 | 1.004 | 1.742 | NcNcUp | Unknown |
| TFB6 | 0.841 | 0.838 | 0.827 | 1.226 | NcNcUp | Unknown |
| THI4 | 0.912 | 0.915 | 0.841 | 1.313 | NcNcUp | Mitochondrion organization; thiamine and thiazole biosynthetic processes |
| TIR1 | 0.558 | 0.591 | 0.560 | 0.807 | NcNcUp | Structural molecule activity |
| TIS11 | 0.750 | 0.836 | 0.843 | 1.469 | NcNcUp | Nuclear-transcribed mRNA catabolic process; cellular iron ion homeostasis |
| TPK2 | 0.819 | 0.838 | 0.717 | 1.531 | NcNcUp | Invasive growth in response to glucose limitation; protein phosphorylation; Ras protein signal transduction; PKA signaling |
| TPO2 | 0.513 | 0.414 | 0.393 | 0.752 | NcNcUp | Spermine transport |
| TPO4 | 0.506 | 0.584 | 0.526 | 1.831 | NcNcUp | Spermine and spermidine transport |
| TPS2 | 0.884 | 0.913 | 0.895 | 1.314 | NcNcUp | Trehalose biosynthetic process; cellular response to heat |
| TPS3 | 0.759 | 0.784 | 0.730 | 1.073 | NcNcUp | Trehalose biosynthetic process |
| TRR1 | 0.934 | 0.899 | 0.858 | 1.395 | NcNcUp | Cellular response to oxidative stress; cell redox homeostasis |
| TRX2 | 0.614 | 0.657 | 0.587 | 1.223 | NcNcUp | Golgi vesicle transport; vacuole organization |
| TSA1 | 0.504 | 0.509 | 0.514 | 1.033 | NcNcUp | Cellular response to DNA damage stimulus; protein folding; response to oxidative stress |
| VHR1 | 0.823 | 0.863 | 0.953 | 1.157 | NcNcUp | Response to oxidative stress; chronological cell aging |
| VHS1 | 0.855 | 0.720 | 0.848 | 1.499 | NcNcUp | G1/S transition of mitotic cell cycle; protein phosphorylation |
| VID27 | 1.082 | 1.020 | 1.003 | 1.683 | NcNcUp | Unknown |
| YBR071W | 0.938 | 1.002 | 0.912 | 1.771 | NcNcUp | Unknown |
| YBR137W | 0.878 | 0.965 | 0.905 | 1.313 | NcNcUp | Post-translational protein targeting to membrane |
| YCF1 | 1.080 | 1.067 | 1.000 | 1.621 | NcNcUp | Glutathione metabolic process; response to metal ion; billrubin transport; non-autophagic vacuole fusion; cell redox homeostasis |
| YCR100C | 1.246 | 1.378 | 1.241 | 1.813 | NcNcUp | Unknown |
| YDC1 | 0.800 | 0.901 | 0.853 | 1.162 | NcNcUp | Ceramide biosynthetic process |
| YDL180W | 1.051 | 1.048 | 1.054 | 1.556 | NcNcUp | Unknown |
| YDR061W | 0.940 | 0.897 | 0.905 | 1.357 | NcNcUp | Unknown |
| YDR132C | 0.967 | 0.941 | 0.774 | 2.291 | NcNcUp | Unknown |
| YER079W | 0.696 | 0.723 | 0.583 | 1.364 | NcNcUp | Unknown |
| YER085C | 0.917 | 1.040 | 1.018 | 2.545 | NcNcUp | Unknown |
| YER121W | 0.760 | 0.770 | 0.815 | 1.938 | NcNcUp | Unknown |
| YET3 | 0.791 | 0.869 | 0.869 | 1.223 | NcNcUp | Unknown |
| YFL054C | 1.094 | 1.126 | 1.055 | 1.777 | NcNcUp | Water transport |
| YGL114W | 1.229 | 1.171 | 1.131 | 2.130 | NcNcUp | Oligopeptide transport |
| YGR066C | 1.056 | 1.093 | 0.915 | 1.570 | NcNcUp | Unknown |
| YGR122W | 1.050 | 1.048 | 1.071 | 1.533 | NcNcUp | Negative regulation of transcription from RNA Pol II promoter |
| YGR250C | 0.985 | 0.962 | 0.884 | 1.618 | NcNcUp | Unknown |
| YHK8 | 1.631 | 1.325 | 1.141 | 4.195 | NcNcUp | Drug transport |
| YIL102C | 0.703 | 0.831 | 0.759 | 1.090 | NcNcUp | Unknown |
| YJR115W | 1.230 | 1.165 | 1.369 | 1.917 | NcNcUp | Unknown |
| YJR149W | 0.694 | 0.655 | 0.563 | 1.071 | NcNcUp | Unknown |
| YKL091C | 0.744 | 0.761 | 0.757 | 1.044 | NcNcUp | Unknown |
| YKR011C | 0.689 | 0.779 | 0.716 | 1.081 | NcNcUp | Unknown |
| YLL053C | 0.937 | 0.870 | 0.912 | 1.322 | NcNcUp | Unknown |
| YLR042C | 0.906 | 0.905 | 0.915 | 1.486 | NcNcUp | Unknown |
| YLR049C | 1.061 | 1.079 | 1.127 | 1.676 | NcNcUp | Unknown |
| YLR177W | 0.972 | 1.021 | 1.071 | 1.569 | NcNcUp | Unknown |
| YLR194C | 0.852 | 0.839 | 0.868 | 1.238 | NcNcUp | Cell wall organization |
| YLR257W | 0.833 | 0.814 | 0.817 | 1.415 | NcNcUp | Unknown |
| YLR312C | 0.869 | 0.740 | 0.837 | 1.443 | NcNcUp | Nucleophagy; reticulophagy |
| YLR345W | 0.822 | 0.851 | 0.812 | 1.239 | NcNcUp | Fructose 2,6-bisphosphate metabolic process |
| YML131W | 1.153 | 1.114 | 0.924 | 1.804 | NcNcUp | Unknown |
| YMR210W | 0.736 | 0.766 | 0.819 | 1.212 | NcNcUp | Medium-chain fatty acid biosynthetic process |
| YMR262W | 0.765 | 0.768 | 0.799 | 1.280 | NcNcUp | Unknown |
| YMR317W | 0.313 | 0.296 | 0.282 | 0.722 | NcNcUp | Unknown |
| YNL046W | 0.976 | 1.099 | 1.017 | 1.454 | NcNcUp | Unknown |
| YNL134C | 0.685 | 0.682 | 0.586 | 1.428 | NcNcUp | Response to furfural |
| YNL144C | 1.372 | 1.265 | 1.073 | 2.572 | NcNcUp | Unknown |
| YNL195C | 0.812 | 0.810 | 0.874 | 1.215 | NcNcUp | Unknown |
| YNL200C | 0.759 | 0.746 | 0.780 | 1.187 | NcNcUp | Nicotinamide nucleotide metabolic process |
| YNR068C | 0.940 | 0.977 | 0.859 | 1.790 | NcNcUp | Unknown |
| YOR214C | 0.776 | 0.900 | 0.732 | 2.838 | NcNcUp | Unknown |
| YOR268C | 0.745 | 0.776 | 0.728 | 1.360 | NcNcUp | Unknown |
| YOR289W | 0.769 | 0.769 | 0.724 | 1.141 | NcNcUp | Unknown |
| YPL247C | 0.920 | 0.905 | 0.825 | 1.456 | NcNcUp | Unknown |
| YPL272C | 0.918 | 1.016 | 0.969 | 1.757 | NcNcUp | Unknown |
| YPS5 | 0.797 | 0.759 | 0.735 | 1.429 | NcNcUp | Unknown |
| YRR1 | 1.035 | 1.020 | 1.011 | 1.657 | NcNcUp | Response to oxidative stress |
| YSW1 | 0.782 | 0.836 | 0.837 | 1.160 | NcNcUp | Ascospore-type prospore membrane assembly |
| ZEO1 | 0.641 | 0.739 | 0.772 | 1.080 | NcNcUp | Cell wall organization |
| ZPR1 | 0.999 | 0.927 | 0.963 | 1.534 | NcNcUp | Regulation of mitotic cell cycle; cellular response to starvation |
| ZTA1 | 1.017 | 1.011 | 0.948 | 1.429 | NcNcUp | Cellular response to oxidative stress |
| ICS3 | 0.659 | 0.716 | 0.787 | 0.940 | NcNcNc | Copper ion homeostasis |
| IDP3 | 0.956 | 1.137 | 1.348 | 1.118 | NcNcNc | Fatty acid beta-oxidation; NADPH regeneration |
| SUR7 | 0.714 | 0.834 | 0.896 | 1.072 | NcNcNc | Endocytosis; ascospore formation |
| TOS3 | 1.095 | 1.216 | 1.351 | 1.597 | NcNcNc | Glucose metabolic process; protein phosphorylation |
| YGL193C | 0.806 | 0.943 | 0.989 | 1.167 | NcNcNc | Unknown |
| ACO2 | 1.103 | 1.053 | 1.052 | 0.594 | NcNcDn | Cellular amino acid metabolic processes |
| AQR1 | 0.997 | 1.066 | 1.082 | 0.480 | NcNcDn | Drug transmembrane transport; monocarboxylic acid transport; amino acid export |
| ARG5,6 | 1.844 | 1.883 | 1.871 | 1.103 | NcNcDn | Transcription regulation; arginine and ornithine biosynthetic process |
| ARX1 | 1.175 | 1.057 | 1.156 | 0.670 | NcNcDn | Ribosomal large subunit export from nucleus |
| BIO2 | 1.027 | 1.013 | 1.060 | 0.590 | NcNcDn | Biotin biosynthetic process |
| COS12 | 0.612 | 0.651 | 0.701 | 0.363 | NcNcDn | Unknown |
| DAL1 | 5.941 | 5.338 | 4.587 | 2.958 | NcNcDn | Hydrolase activity |
| DAT1 | 1.061 | 1.016 | 1.062 | 0.614 | NcNcDn | Negative regulation of transcription from RNA Pol II promoter |
| FAR1 | 1.453 | 1.335 | 1.520 | 0.808 | NcNcDn | Pheromone-dependent signal transduction involved in conjugation with cellular fusion |
| FUS2 | 2.332 | 2.400 | 2.357 | 1.237 | NcNcDn | Karyogamy involved in conjugation with cellular fusion; mitotic cell cycle arrest in response to pheromone; maintenance of protein location in nucleus |
| GFD2 | 1.372 | 1.349 | 1.362 | 0.731 | NcNcDn | Unknown |
| GLT1 | 1.310 | 1.333 | 1.160 | 0.648 | NcNcDn | Glutamate biosynthetic process; ammonia assimilation cycle |
| GUP2 | 1.227 | 1.380 | 1.383 | 0.712 | NcNcDn | Glycerol transport |
| IMP3 | 1.134 | 1.123 | 1.171 | 0.665 | NcNcDn | rRNA processing; ribosomal small subunit biogenesis |
| KAR4 | 1.498 | 1.504 | 1.585 | 0.879 | NcNcDn | Karyogamy involved in conjugation with cellular fusion; meiotic nuclear division; positive and negative regulation of transcription by pheromones |
| KRE33 | 1.106 | 1.119 | 0.994 | 0.663 | NcNcDn | Ribosomal small subunit biogenesis |
| KTI11 | 1.211 | 1.369 | 1.331 | 0.581 | NcNcDn | tRNA wobble uridine modification; peptidyl-diphthamide biosynthetic process from peptidyl histidine |
| MET2 | 1.050 | 1.058 | 1.172 | 0.615 | NcNcDn | Sulfur amino acid metabolic process; methionine biosynthetic process; homoserine metabolic process |
| MMP1 | 1.241 | 1.187 | 1.263 | 0.645 | NcNcDn | S-methylmethionine transport |
| NCS2 | 1.086 | 1.089 | 1.139 | 0.644 | NcNcDn | Invasive growth in response to glucose limitation; tRNA wobble uridine thiolation; pseudohyphal growth; protein urmylation |
| NOG2 | 1.411 | 1.341 | 1.392 | 0.714 | NcNcDn | Ribosomal large subunit export from nucleus |
| NSR1 | 1.732 | 1.487 | 1.550 | 0.678 | NcNcDn | Ribosomal small subunit assembly; rRNA processing |
| PAI3 | 2.344 | 2.191 | 2.048 | 1.153 | NcNcDn | Protein catabolic process in the vacuole |
| PRM1 | 2.599 | 2.633 | 2.566 | 1.244 | NcNcDn | Plasma membrane fusion involved in cytogamy |
| PRM3 | 2.058 | 2.061 | 2.218 | 1.156 | NcNcDn | Karyogamy involved in conjugation with cellular fusion |
| PRM6 | 4.729 | 5.155 | 4.170 | 0.721 | NcNcDn | Potassium ion transmembrane transport |
| PRM8 | 2.857 | 2.599 | 2.314 | 1.073 | NcNcDn | Pheromone response |
| RCL1 | 1.043 | 1.102 | 1.161 | 0.620 | NcNcDn | Endonucleolytic cleavage to generate mature 5'-end of SSUrRNA from (SSU-rRNA, 5.8S rRNA, LSU-rRNA) |
| RIX7 | 1.127 | 1.045 | 1.061 | 0.662 | NcNcDn | Ribosomal large subunit export from nucleus |
| RPA12 | 0.916 | 1.082 | 1.096 | 0.434 | NcNcDn | Transcription from RNA Pol I promoter; termination of RNA Pol I transcription; transcription of nuclear large rRNA transcript |
| RPL22B | 1.013 | 1.158 | 1.151 | 0.493 | NcNcDn | Cytoplasmic translation |
| RPL7B | 1.102 | 1.201 | 1.278 | 0.538 | NcNcDn | Ribosomal large subunit biogenesis; cytoplasmic translation |
| RRN11 | 1.177 | 1.125 | 1.259 | 0.597 | NcNcDn | Transcription of nuclear large rRNA transcript |
| RRS1 | 1.057 | 1.059 | 1.102 | 0.622 | NcNcDn | Ribosomal large subunit export from nucleus; endonucleolytic cleavage in ITS1 to separate SSU-rRNA from 5.8S rRNA and LSU-rRNA from tricistronic rRNA transcript |
| RSA4 | 1.023 | 1.047 | 1.028 | 0.514 | NcNcDn | Ribosomal large subunit assembly |
| SAM1 | 1.080 | 0.943 | 0.944 | 0.564 | NcNcDn | Methionine metabolic process |
| SIP4 | 2.582 | 2.517 | 2.399 | 1.405 | NcNcDn | Positive regulation of gluconeogenesis |
| SSB1 | 1.761 | 1.443 | 1.532 | 0.837 | NcNcDn | Protein folding, export of ribosomal subunit from nucleus; regulation of translational fidelity; rRNA processing |
| UBC11 | 1.071 | 1.019 | 1.011 | 0.506 | NcNcDn | Protein ubiquitination |
| URA7 | 1.055 | 1.000 | 0.960 | 0.609 | NcNcDn | CTP biosynthetic process; phospholipid biosynthetic process |
| UTP10 | 1.141 | 1.070 | 1.006 | 0.683 | NcNcDn | Maturation of SSU-rRNA from tricistronic rRNA transcript; positive regulation of transcription from RNA Pol I promoter |
| UTP13 | 1.122 | 1.048 | 1.064 | 0.632 | NcNcDn | Maturation of SSU-rRNA from tricistronic rRNA transcript |
| UTP20 | 1.076 | 1.118 | 0.989 | 0.627 | NcNcDn | Endonucleolytic cleavage to generate mature 5'-end of SSU-rRNA from (SSU-rRNA, 5.8S rRNA, LSU-rRNA) |
| YAL065C | 0.893 | 0.862 | 0.731 | 0.516 | NcNcDn | Unknown |
| YDR374C | 1.894 | 2.005 | 1.813 | 1.093 | NcNcDn | Regulation of mRNA stability |
| YHL012W | 1.245 | 1.317 | 1.253 | 0.697 | NcNcDn | Unknown |
| YIL082W-A | 3.923 | 4.274 | 4.131 | 2.312 | NcNcDn | RNA mediated transposition |
| YKR041W | 2.170 | 1.874 | 2.000 | 0.845 | NcNcDn | Unknown |
| YLR363W-A | 1.223 | 1.308 | 1.332 | 0.628 | NcNcDn | Unknown |
| YMR147W | 0.905 | 0.830 | 0.794 | 0.392 | NcNcDn | Unknown |
| AAD16 | 1.173 | 1.040 | 0.720 | 2.919 | NcDnUp | Unknown |
| AAD4 | 1.234 | 1.083 | 0.545 | 4.422 | NcDnUp | Cellular aldehyde metabolic process |
| AGX1 | 0.847 | 0.801 | 0.619 | 1.643 | NcDnUp | Glycine biosynthetic process by transamination of glyoxylate |
| ALD3 | 0.928 | 0.957 | 0.384 | 0.927 | NcDnUp | Polyamine catabolic process; beta-alanine biosynthetic process |
| APJ1 | 0.854 | 0.927 | 0.594 | 1.670 | NcDnUp | Protein sumoylation |
| ARR3 | 1.915 | 2.013 | 1.206 | 4.185 | NcDnUp | Antimonite and arsenite transport |
| BSC5 | 1.134 | 1.024 | 0.755 | 3.045 | NcDnUp | Unknown |
| BTN2 | 0.821 | 0.883 | 0.260 | 1.737 | NcDnUp | Protein folding; amino acid transport; pH regulation; intracellular protein transport; protein localization to nucleus; retrograde transport (endosome to Golgi) |
| CIN5 | 1.420 | 1.362 | 0.713 | 1.858 | NcDnUp | Hyperosmotic salinity response; response to drug |
| CRC1 | 0.557 | 0.518 | 0.403 | 1.006 | NcDnUp | Fatty acid metabolic process |
| CUR1 | 0.731 | 0.762 | 0.547 | 1.387 | NcDnUp | Protein folding; intracellular protein transport; protein localization to nucleus; cellular response to heat |
| DDR2 | 0.746 | 0.667 | 0.152 | 0.920 | NcDnUp | Cellular response to DNA damage stimulus, starvation, heat, and hydrogen peroxide |
| DTR1 | 0.985 | 1.100 | 0.825 | 2.301 | NcDnUp | Amine transport; ascospore wall assembly |
| ECM4 | 0.769 | 0.733 | 0.510 | 1.875 | NcDnUp | Glutathione metabolic process |
| FLR1 | 1.238 | 1.095 | 0.739 | 2.167 | NcDnUp | Drug transmembrane transport |
| GTT2 | 1.161 | 0.947 | 0.673 | 2.612 | NcDnUp | Glutathione metabolic process |
| HBN1 | 0.847 | 0.800 | 0.444 | 0.624 | NcDnUp | Unknown |
| HSP26 | 0.495 | 0.517 | 0.309 | 0.708 | NcDnUp | Cellular response to heat; protein folding |
| HSP42 | 0.736 | 0.827 | 0.243 | 1.475 | NcDnUp | Cytoskeleton organization |
| HSP78 | 0.779 | 0.770 | 0.585 | 1.387 | NcDnUp | Mitochondrial genome maintenance; cellular response to heat; protein refolding, unfolding, and stabilization |
| ICY2 | 0.800 | 0.820 | 0.638 | 1.453 | NcDnUp | Mitophagy |
| MCH2 | 1.381 | 1.179 | 0.741 | 1.062 | NcDnUp | Transport |
| MTL1 | 0.814 | 0.969 | 0.732 | 1.265 | NcDnUp | Response to oxidative stress; cell wall organization; response to glucose starvation |
| OYE3 | 0.528 | 0.479 | 0.323 | 3.516 | NcDnUp | Apoptotic process |
| PES4 | 0.927 | 1.013 | 0.796 | 1.684 | NcDnUp | Unknown |
| RCR2 | 0.985 | 0.998 | 0.772 | 1.437 | NcDnUp | Vesicle-mediated transport |
| RPI1 | 0.367 | 0.374 | 0.183 | 0.488 | NcDnUp | Ras protein signal transduction; cell wall biogenesis |
| RPM2 | 0.813 | 0.871 | 0.596 | 1.534 | NcDnUp | tRNA 5'-leader removal; cytoplasmic translation; mRNA processing; mitochondrion organization |
| SLZ1 | 0.908 | 0.947 | 0.734 | 1.452 | NcDnUp | Meiotic nuclear division; mRNA methylation; negative regulation of pseudohyphal growth |
| SSA4 | 1.001 | 0.958 | 0.422 | 1.336 | NcDnUp | Protein folding; response to heat; SRP-dependent cotranslational protein targeting to membrane |
| SXM1 | 0.013 | 0.012 | 0.007 | 0.010 | NcDnUp | Nuclear transport |
| TSA2 | 0.827 | 0.800 | 0.392 | 0.750 | NcDnUp | cellular response to oxidative stress; cell redox homeostasis |
| YCR102C | 1.145 | 1.173 | 0.498 | 2.161 | NcDnUp | Response to copper ion |
| YDL218W | 0.910 | 0.785 | 0.321 | 1.170 | NcDnUp | Unknown |
| YDR034W-B | 1.387 | 1.370 | 0.757 | 2.079 | NcDnUp | Unknown |
| YER158C | 0.804 | 0.772 | 0.556 | 1.220 | NcDnUp | Unknown |
| YJL144W | 0.895 | 0.816 | 0.205 | 1.759 | NcDnUp | Cellular response to water deprivation |
| YKL070W | 1.097 | 0.965 | 0.734 | 6.759 | NcDnUp | Unknown |
| YKL071W | 2.152 | 1.767 | 0.605 | 3.120 | NcDnUp | Unknown |
| YLR108C | 0.874 | 0.834 | 0.577 | 2.293 | NcDnUp | Unknown |
| YLR149C | 0.720 | 0.670 | 0.498 | 1.461 | NcDnUp | Unknown |
| YLR297W | 1.182 | 1.383 | 0.413 | 2.108 | NcDnUp | Unknown |
| YMR090W | 1.143 | 1.182 | 0.829 | 1.697 | NcDnUp | Unknown |
| YMR279C | 2.825 | 2.946 | 2.004 | 5.161 | NcDnUp | Borate transmembrane transport |
| FKS3 | 1.075 | 1.153 | 0.730 | 0.624 | NcDnNc | Ascospore wall assembly |
| IME4 | 1.044 | 0.904 | 0.577 | 0.443 | NcDnDn | Meiotic nuclear division; mRNA methylation; negative regulation of pseudohyphal growth |
| SPO74 | 1.023 | 0.921 | 0.732 | 0.549 | NcDnDn | Ascospore formation |
| YOR338W | 0.899 | 0.903 | 0.719 | 0.536 | NcDnDn | Unknown |
| YLR464W | 0.850 | 0.465 | 0.572 | 0.694 | DnUpUp | Unknown |
| YNR077C | 0.782 | 0.515 | 0.772 | 1.114 | DnUpUp | Unknown |
| YPR202W | 1.916 | 1.058 | 1.270 | 1.673 | DnUpUp | Unknown |
| HSP32 | 0.985 | 0.395 | 0.767 | 0.724 | DnUpNc | Cellular response to nutrient levels |
| HSP33 | 0.945 | 0.409 | 0.734 | 0.640 | DnUpNc | Cellular response to nutrient levels |
| HXT11 | 1.301 | 0.633 | 0.838 | 0.946 | DnUpNc | Hexose transport |
| SNO4 | 0.634 | 0.243 | 0.540 | 0.604 | DnUpNc | Cellular response to nutrient levels |
| YEL076C-A | 0.848 | 0.470 | 0.593 | 0.707 | DnUpNc | Unknown |
| YGL260W | 0.881 | 0.444 | 0.661 | 0.665 | DnUpNc | Unknown |
| BSC1 | 0.470 | 0.359 | 0.601 | 0.202 | DnUpDn | Unknown |
| BSC4 | 1.104 | 0.613 | 1.166 | 0.931 | DnUpDn | Unknown |
| ILV5 | 1.039 | 0.807 | 1.090 | 0.615 | DnUpDn | Mitochondrial genome maintenance; branched-chain amino acid biosynthetic process |
| MAM1 | 0.968 | 0.351 | 1.544 | 1.124 | DnUpDn | Meiotic sister chromatid cohesion involved in meiosis I; meiotic chromosome segregation |
| PPM2 | 1.013 | 0.652 | 0.830 | 0.315 | DnUpDn | tRNA methylation; wybutosine biosynthetic process |
| YHR214C-E | 0.881 | 0.618 | 0.745 | 0.417 | DnUpDn | Unknown |
| FRE2 | 0.504 | 0.354 | 0.304 | 1.012 | DnNcUp | Iron ion transport; copper ion import |
| IME2 | 1.826 | 1.050 | 0.967 | 2.051 | DnNcUp | Protein phosphorylation; regulation of meiotic nuclear division |
| POT1 | 0.616 | 0.489 | 0.416 | 0.947 | DnNcUp | Fatty acid beta-oxidation |
| SOR1 | 1.015 | 0.759 | 0.726 | 1.572 | DnNcUp | Hexose metabolic process |
| SOR2 | 0.941 | 0.721 | 0.751 | 1.606 | DnNcUp | Hexose metabolic process |
| YLR156W | 0.810 | 0.464 | 0.430 | 0.891 | DnNcUp | Unknown |
| YLR159W | 0.811 | 0.463 | 0.429 | 0.896 | DnNcUp | Unknown |
| YLR161W | 0.800 | 0.460 | 0.434 | 0.882 | DnNcUp | Unknown |
| BFA1 | 1.282 | 0.830 | 0.833 | 0.719 | DnNcNc | Negative regulation of exit from mitosis; mitotic spindle orientation checkpoint |
| PFF1 | 1.170 | 0.833 | 0.906 | 0.685 | DnNcDn | Unknown |
| SSB2 | 1.517 | 1.141 | 1.170 | 0.816 | DnNcDn | Ribosomal subunit export from nucleus; cytoplasmic translation; rRNA processing; regulation of translational fidelity; translation frameshifting; cellular response to glucose starvation; 'de novo' cotranslational protein folding |
| YAR064W | 2.414 | 1.668 | 1.905 | 1.134 | DnNcDn | Unknown |
| AAD15 | 2.007 | 1.194 | 0.736 | 1.566 | DnDnUp | Cellular aldehyde metabolic process |
| AAD6 | 1.130 | 0.804 | 0.553 | 2.657 | DnDnUp | Cellular aldehyde metabolic process |
| ATP10 | 0.980 | 0.735 | 0.577 | 0.960 | DnDnUp | Mitochondrial proton-transporting ATP synthase complex assembly |
| FRM2 | 1.226 | 0.815 | 0.590 | 1.017 | DnDnUp | Cellular response to oxidative stress; negative regulation of fatty acid metabolic process |
| HSP30 | 0.793 | 0.585 | 0.115 | 2.549 | DnDnUp | Cellular response to DNA damage stimulus, heat, hydrogen peroxide, osmotic stress, and ethanol; negative regulation of ATPase activity |
| HXT17 | 1.219 | 0.959 | 0.613 | 1.246 | DnDnUp | Hexose transport |
| JIP4 | 0.940 | 0.643 | 0.378 | 0.970 | DnDnUp | Unknown |
| NCA3 | 0.243 | 0.156 | 0.111 | 0.265 | DnDnUp | Mitochondrion organization |
| SRX1 | 0.849 | 0.594 | 0.213 | 2.193 | DnDnUp | Cellular response to oxidative stress |
| YMR084W | 0.568 | 0.453 | 0.289 | 1.021 | DnDnUp | Unknown |
| DCV1 | 1.573 | 1.028 | 0.742 | 0.739 | DnDnNc | Unknown |
| YJL160C | 1.572 | 1.187 | 0.732 | 0.833 | DnDnNc | Unknown |
