# Supplementary material for: Microarray Analysis of Gene Expression in Saccharomyces cerevisiae kap108Δ Mutants upon Addition of Oxidative Stress
Source: G3 (Bethesda). 2016 Feb 17;6(4):1131–9. doi: 10.1534/g3.116.027011 (PMC4825647; doi:10.1534/g3.116.027011)
Supplement: Supporting Information [file supp_g3.116.027011_FigureS3.zip › Supplementary Figure S3.pdf]

GOID	GO term	Level	% in user set	% in Yeast ract	p-value	Genes/ORF
GO:0051792	medium-chain fatty acid biosynthetic process	7	0.59%	100.00%	0.000000000000000	EHT1 YMR210W EEB1
GO:0	medium-chain fa		0.	10	0.000000	

051793	tty acid catabolic process	7	39%	0.00%	000000000	EHT1 EEB1
GO:0015837	amine transport	4	0.20%	100.00%	0.000000000000000	DTR1
GO:0045922	negative regulation of fatty acid meta[illegible]	7	0.20%	100.00%	0.000000000000[illegible]	FRM2

	abolic process				000	
[GO:0006565]	L-serine catabolic process	7	0.20%	100.00%	0.000000000000000	CHA1
GO:00	positive regulation of proteasomal ubiquitin	8	0.2	100.0	0.0000000	[illegible]

32436	-dependent protein catabolic process		0 %	0 0 %	000000000	RPN4
GO:009	negative regulation of transcription from RNA	7	0.39	100.0	0.000000000	RPN4 ROX1

[illegible]7201	[illegible] polymerase II promoter in response to stress		[illegible]%	[illegible]0%	[illegible]0000000	[illegible]
GO:0006069	ethanol oxidation	5	0.20%	100.00%	0.000000000000000	SFA1
GO:0[illegible]	L-glutamate [illegible]	[illegible]	0[illegible]	10[illegible]	0.000000[illegible]	[illegible]

…0097054	…biosynthetic process	8	…0.20%	…0.00%	…0000000000	GLT1
GO:0045185	maintenance of protein location	4	0.20%	100.00%	0.000000000000000	GAL3
GO:0010995	free ubiquitin chain depolymerizati…	6	0.20%	100.00%	0.000000000000000	DOA4

	ation					
[GO:0060275]()	maintenance of stationary phase in response to starvation	5	0.20%	100.00%	0.000000000000000	[GIS1]()
G	positive regulation of mito				0.000	

O:0010892	chondrial translation in response to stress	7	0.20%	100.00%	000000000000	HSP78
GO:0000950	branched-chain amino acid catabolic process to al	7	0.20%	100.00%	0.0000000000000	ARO10

	cohol via Ehrlich pathway				00	
[GO:0000951]	methionine catabolic process to 3-methylthiopropanol	6	0.20%	100.00%	0.000000000000000	[ARO10]
	ener				0.0	

GO:0006112	gy reserve metabolic process	6	0.39%	100.00%	00000000000000	RGI1 RGI2
GO:0010789	meiotic sister chromatid cohesion involved in meiosis I	5	0.20%	100.00%	0.000000000000000	MAM1
	cellular				0.00	

[GO:0071470]	, response to osmotic stress	5	0.20%	100.00%	0000000000000	[HSP12]
[GO:0030031]	cell projection assembly	5	0.20%	100.00%	0.000000000000000	[STE2]
[GO:0019]	glycine biosynthetic process, by tra	8	0.20%	100.00	0.000000000	[AGX1]

265	nsamination of glyoxylate			%	000000	
GO:0045818	negative regulation of glycogen catabolic process	8	0.20%	100.00%	0.000000000000000	IGD1
GO:0	cellular re		0	10	0.000000	

[0071467](#)	response to pH	5	0.20%	00.00%	0000000000	[RIM8](#)
[GO:0006636](#)	unsaturated fatty acid biosynthetic process	7	0.20%	100.00%	0.000000000000000	[OLE1](#)
[GO:0000737](#)	DNA catabolic process, endo	7	0.20%	100.00%	0.000000000000	[SAE2](#)

	nucleolytic				000	
GO:0080009	mRNA methylation	6	0.20%	100.00%	0.000000000000000	IME4
GO:0033540	fatty acid beta-oxidation using acyl-CoA oxidase	8	0.20%	100.00%	0.000000000000000	POX1

GO:0006198	cAMP catabolic process	8	0.20%	100.00%	0.000000000000000	PDE1
GO:0034504	protein localization to nucleus	7	0.39%	100.00%	0.000000000000000	BTN2 CUR1
GO:0052837	thiazole biosynthetic proces[illegible]	6	0.20%	100.00%	0.0000000000000[illegible]	THI4

	s				00	
[GO:0015931]()	nucleobase-containing compound transport	5	0.20%	100.00%	0.000000000000000	[SNG1]()
[GO:0051186]()	cofactor metabolic process	4	0.20%	100.00%	0.000000000000000	[BIO2]()
					0.0	

[GO:0015771]()	trehalose transport	7	0.20%	100.00%	00000000000000	[MAL11]()
[GO:0009237]()	siderophore metabolic process	5	0.20%	100.00%	0.000000000000000	[ARN2]()
	regulation of transcription fr					

GO:0061420	[illegible]omRNA polymerase II promoter in response to biotin starvation	6	0.20%	100.00%	0.000000000000000	VHR1
GO:0009442	allantoin assimilation path[illegible]	7	0.20%	100.00%	0.000000000000[illegible]	DAL1

	[illegible]way				000	
[GO:1900474]	negative regulation of mating type switching by negative regulation of transcription	7	0.20%	100.00%	0.000000000000000	ASH1

	n from RNA polymerase II promoter					
	negative regulation of G1/S transition of mitotic cell				0.	

GO:1900477	cycle by negative regulation of transcription from RNA polymerase II promoter	10	0.20%	100.00%	[illegible]000000000000000	ASH1
	regulatio					

GO:0090337	n of formin-nucleated actin cable assembly	7	0.20%	100.00%	0.000000000000000	RHO4
GO:0042939	tripeptide transport	7	0.20%	100.00%	0.000000000000000	PTR2
GO:00[illegible]	S-methylmethio[illegible]	5	0.2[illegible]	100.[illegible]	0.0000000[illegible]	

15806	ne transport	5	0%	00%	00000000	MMP1
GO:0000167	activation of MAPKKK activity involved in osmosensory signaling pathw	6	0.20%	100.00%	0.000000000000000	SSK1

	ay					
GO:0006788	heme oxidation	5	0.20%	100.00%	0.000000000000000	HMX1
GO:0042167	heme catabolic process	5	0.20%	100.00%	0.000000000000000	HMX1
GO:0006805	xenobiotic metabolic pr	4	0.20%	100.00%	0.000000000000	ECM38

	ocess				000	
[GO:0033194]()	response to hydroperoxide	5	0.20%	100.00%	0.000000000000000	[TSA1]()
[GO:0042262]()	DNA protection	5	0.20%	100.00%	0.000000000000000	[TSA1]()
[GO:003]()	borate transmembr	6	0.20	100.0	0.00000000	[YMR279C]()

5445	ane transport		%	0%	0000000	
GO:0000040	low-affinity iron ion transport	7	0.20%	100.00%	0.000000000000000	FET4
GO:0032973	amino acid export	6	0.20%	100.00%	0.000000000000000	AQR1
	plasma mam					

GO:0032220	membrane fusion involved in cytogamy	4	0.20%	100.00%	0.0000000000000000	PRM1
GO:0001578	microtubule bundle formation	6	0.20%	100.00%	0.0000000000000000	ASE1
GO:0	spindle midz		0.	10	0.0000000	

[illegible]0051255	[illegible]dzone assembly	5	[illegible]20%	[illegible]0.00%	[illegible]000000000	ASE1
GO:0035952[illegible]	negative regulation of oligopeptide transport by negative regulation[illegible]	7	0.20%	100.00%	0.000000000000[illegible]	CUP9

	n of transcription from RNA polymerase II promoter				000	
[GO:0048288](GO:0048288)	nuclear membrane fusion involved d	7	0.200%	100.00%	0.000000000000	[PRM3](PRM3)

	d in karyogamy				000	
[GO:0015699]	antimonite transport	7	0.20%	100.00%	0.000000000000000	ARR3
[GO:0008150]	biological_process	1	30.00%	12.97%	0.000000000000002	FUN19 YAL065C YAR064W YBL029W MOH1 FMP23 YBR071W YBR137W YBR184W YCL001W-AYCL001W-B HBN1 GFD2 YCL068C YCR100C PAU3 YDL009C RTK1 BSC1 YET3 YDL180W YDL218WYDR034W-B YDR061W YDR124W ECM18 YDR132C YDR374C JIP4 SNA2 YEL057C YEL076C-A YER079WYER085C YER121W YER158C DCV1 YFR012W-A PES4 RRT5 YGL010W YGL015C PRM8 RRT6 YGL193CYGL230C YGL258W-A YPS5 YGL260W COS12 YGR016W YGR018C YGR066C TDA10 RTA1 YGR250CYHL012W AIM17 OCA5 ECM12 YHR022C AIM46 YHR210C YHR214C-D YHR214C-E YIL054W YIL102CPAU15 YIR042C IRC18 ICS3 SET4 YJL160C FMP33 ACO2 REE1 MHO1 YJR012C ECM27 YJR115WYJR149W DAN4 YKL070W YKL071W YKL091C YKR011C YKR041W PAU17 YLL053C YLR042C YLR049CYLR108C YLR149C YLR156W YLR159W YLR161W SHH4 YLR177W YLR257W YLR297W YLR312C TMA10YLR363W-A YLR446W YLR464W YML131W STB2 YMR084W YMR085W YMR090W YMR147W ICY1YMR206W YMR262W YMR317W SNO4 PAU19 YNL018C YNL046W YNL092W YNL134C YNL144CYNL146W YNL162W-A TDA7 YNL195C SLZ1 VID27 BSC4 YNR068C BSC5 YNR071C YNR077C YOL047CRRT8 AIM39 YOR012W IRC23 YOR152C

						YOR214C YOR268C YOR289W TFB6 HSP33 PAU21 YPL014WLEE1 YPL247C YPL272C HSP32 PAU22 CMR3 YPR202W
GO:0006950	response to stress	3	7.45%	25.17%	0.000000000000687	HSP26 HSP30 PAU3 MRK1 SSB1 AAD4 TPS2 HSP42 HSP78 TIR1 SSA4 HSP12 DAK2 TOS3 MTL1 NSR1RTA1 FMP43 WSC4 XBP1 POG1 PAU15 DAN1 DAN4 PAU17 RIM11 DDR48 TPS3 PAU19 SSB2 MDJ2DDR2 ZEO1 YOR338W HSP33 PAU21 HSP32 PAU22
GO:0055114	oxidation-reduction process	4	10.00%	17.47%	0.000000000472267	ZTA1 ADH5 GPX2 FRM2 HBN1 YCR102C ADH7 AAD3 NDE2 SFA1 GLT1 AAD4 SOR2 TRR1 TSA2 ARG5,6SER3 AAD6 AAD16 OLE1 POX1 AIM17 BNA2 SOD1 YJR149W SOR1 GPX1 OAR1 YKL070W YKL071WSRX1 FRE2 ECM4 AHP1 HMX1 FRE1 ILV5 TSA1 CYB2 YML131W ARA2 ALD3 IDP3 YNL134C FRE4 GRE2FRE7 AAD15 IDH2 FRE3 OYE3
GO:00550	iron ion home	8	2.16%	42.31%	0.0000000632	FIT1 ARN1 ARN2 FRE2 FRE1 MMT1 FET4 FRE4 FRE7 FRE3 MMT2

72	ostasis				62595	
GO:0019318	hexose metabolic process	7	0.98%	71.43%	0.000000878578715	SOR2 HXK1 YHR210C SOR1 YNR071C
GO:0015891	siderophore transport	6	0.98%	62.50%	0.000003300568271	FIT1 ARN1 ARN2 FRE4 FRE3
GO:003	ascospore wall	4	2.35	27.94	0.00000574	GIP1 DTR1 SPS22 RRT12 SPS1 SPS100 FKS3 TEP1 SPO21 [illegible]

[illegible]0476	[illegible]assembly		[illegible]%	[illegible]1%	[illegible]2949922	GAS4 MPC54 MUM3
GO:0055085	transmembrane transport	4	6.27%	14.55%	0.000036527183818	FLR1 DTR1 MAL31 GEX1 YDL199C YCF1 PIC2 HXT10 YFL054C YGL114W TPO2 MAL11 ARN2 YHK8HXT4 HXT5 ECM27 MCH2 MRS4 MMP1 ATR1 MMT1 YMR279C FET4 POR1 AQR1 HXT14 HXT17 HXT11TPO4 MMT2 DIP5
GO:0006081	cellular aldehyde metabolic process	4	0.98%	45.45%	0.000045136638862	AAD3 AAD4 AAD6 AAD16 AAD15
	glutat[illegible]				0.0[illegible]	

GO:0006749	[illegible]hione metabolic process	5	0.98%	45.45%	[illegible]00045136638862	GTT1 ECM4 GTT2 ECM38 GTO3
GO:0008645	hexose transport	6	1.18%	37.50%	0.0000061169845925	HXT10 HXT4 HXT5 HXT14 HXT17 HXT11
GO:0000755	cytogamy	5	0.78%	50.00%	0.000087557028723	FIG1 FIG2 STE2 FUS2
					0.	

GO:0015677	copper ion import	7	0.78%	50.00%	[illegible]000087557028723	FRE2 FRE1 FET4 FRE7
GO:0006811	ion transport	4	3.53%	16.36%	0.000272586792918	ATP8 GEX1 HSP30 FIT1 ARN1 ARN2 YHK8 FRE2 FRE1 MMT1 FET4 POR1 ATO2 FRE4 FRE7 FRE3MFM1 MMT2
GO:0034599	cellular response to oxidative st	5	2.55%	18.84%	0.00031743082185	ZTA1 GPX2 FRM2 TRR1 TSA2 GRX2 HSP12 MTL1 TRX2 GPX1 SRX1 AHP1 TSA1

	ress				1	
[GO:0006826]	iron ion transport	8	0.59%	50.00%	0.000351259887107	FRE2 FRE1 FRE7
[GO:0032780]	negative regulation of ATPase activity	7	0.39%	66.67%	0.000368604090856	HSP30 INH1
	negative regul					

GO:2000218	ation of invasive growth in response to glucose limitation	9	0.39%	66.67%	0.000368604090856	DIG2 SIP4
GO:0070009	plasma membrane organization	5	0.39%	66.67%	0.000368604090856	HSP12 NCE102
					0	

GO:0006833	water transport	5	0.39%	66.67%	0.000368604090856	YFL054C AQY2
GO:0016051	carbohydrate biosynthetic process	5	0.39%	66.67%	0.000368604090856	YMR084W YMR085W
GO:0008643	carbohydrate transport	4	1.57%	24.24%	0.0003842556166	MAL31 HXT10 MAL11 HXT4 HXT5 HXT14 HXT17 HXT11

	t				29	
[GO:0022900]	electron transport chain	5	1.96%	20.00%	0.000672145904630	GRX2 OLE1 TRX2 FRE2 SHH4 FRE1 CYB2 FRE4 FRE7 FRE3
[GO:0042149]	cellular response to glucose starvation	6	0.59%	42.86%	0.000773077415330	SSB1 MTL1 SSB2
G[illegible]	sporulation resulti[illegible]				0.000[illegible]	

[GO:0030435](#)	[illegible]ng in formation of a cellular spore	4	3.14%	15.69%	[illegible]0862130737643	[DTR1](#) [SPS22](#) [RRT12](#) [SPS1](#) [SPO74](#) [IME4](#) [SPS100](#) [SPO16](#) [GSM1](#) [IME2](#) [SUR7](#) [FKS3](#) [SPO21](#) [MPC54](#) [MUM3SPS4](#)
[GO:0045454](#)	cell redox homeostasis	5	1.37%	23.33%	0.000954923636504	[TRR1](#) [TSA2](#) [GRX2](#) [TRX2](#) [AHP1](#) [TSA1](#) [POR1](#)
[GO:000712[illegible]](#)	meiosis	4	3.73%	14.29%	0.00120236736[illegible]	[PCH2](#) [AME1](#) [KAR4](#) [SPS1](#) [MAM1](#) [MSH4](#) [RIM8](#) [SAE2](#) [IME4](#) [ZIP2](#) [BNS1](#) [NDT80](#) [SPO16](#) [REC104](#) [IME2](#) [SPO21MPC54](#) [SPS4](#) [MEK1](#)

6					3702	
GO:0006879	cellular iron ion homeostasis	9	1.37%	22.58%	0.001207552345489	ARN2 TIS11 HMX1 MMT1 ISU2 FRE3 MMT2
GO:000043[illegible]	positive regulation of transcription from RNA pol	9	0.39%	50.00%	0.00139541864[illegible]	GAL3 GAL4

5	ymerase II promoter by galactose				2953	
GO:0000296	spermine transport	5	0.39%	50.00%	0.0013954186429 53	TPO2 TPO4
GO:0005	regulation of glycogen	8	0.39%	50.00	0.001395418	PIG2 PCL7

979	biosynthetic process			%	642953	
GO:0006750	glutathione biosynthetic process	6	0.39%	50.00%	0.001395418642953	GSH1 GLO4
GO:0051457	maintenance of protein location in nuc	7	0.39%	50.00%	0.001395418642953	FAR1 SDS22

	leus					
[GO:0000750]()	pheromone-dependent signal transduction involved in conjugation with cellula	5	1.18%	23.08%	0.001870348775913	STE2 MF(ALPHA)2 FAR1 STE18 MFA2 MDG1

	lar fusion					
GO:0006855	drug transmembrane transport	5	0.59%	33.33%	0.002477173609311	FLR1 ATR1 AQR1
GO:0[illegible]	negative regulation of transcription from m[illegible]		0[illegible]	3[illegible]	0.00247[illegible]	

[illegible]0046020	[illegible] RNA polymerase II promoter by pheromones	9	[illegible]0.59%	[illegible]3.33%	[illegible]7173609311	KAR4 PRR2 DIG2
GO:0000742	karyogamy involved in conjugation with[illegible]	4	0.78%	26.67%	0.00307455856885[illegible]	KAR4 PRM2 FUS2 PRM3

	[illegible]cellular fusion				[illegible]5	
GO:0015893	drug transport	4	0.39%	40.00%	0.003302318610192	FLR1 YHK8
GO:0000746	conjugation	4	0.39%	40.00%	0.003302318610192	FIG2 PRM1
[illegible]	'de novo' co[illegible]				0.00[illegible]	

GO:0051083	[illegible]translational protein folding	8	0.39%	40.00%	[illegible]3302318610192	SSB1 SSB2
GO:0043935	sexual sporulation resulting in formation of a cellular spore	5	0.39%	40.00%	0.003302318610192	SPR28 HHT2
					0.	

GO:0015833	peptide transport	5	0.39%	40.00%	003302318610192	YGL114W PTR2
GO:0006102	isocitrate metabolic process	6	0.39%	40.00%	0.003302318610192	IDP3 IDH2
GO:0000092	mitotic anaphase B	7	0.39%	40.00%	0.003302318610192	ASE1 KIP2

					2	
GO:0006631	fatty acid metabolic process	5	1.37%	19.44%	0.003370638265605	FRM2 OLE1 POX1 POT1 OAR1 CRC1 MCT1
GO:0006979	response to oxidative stress	4	0.59%	30.00%	0.003896011930753	GPX2 GPX1 HMX1
GO:0006635	fatty acid beta-oxidation	7	0.59%	30.00%	0.003896011930753	POX1 POT1 IDP3

GO:0009228	thiamine biosynthetic process	7	0.78%	25.00%	0.004211577710048	THI2 THI5 THI4 THI12
GO:0031589	cell-substrate adhesion	4	0.20%	50.00%	0.005150298891532	FLO9
GO:0044210	'de novo' CTP biosynthetic pr	10	0.20%	50.00%	0.0051502988915	URA7

	process				32	
GO:1900464	negative regulation of cellular hyperosmotic salinity response by negative regulation of	7	0.20%	50.00%	0.005150298891532	NRG2

	of transcription from RNA polymerase II promoter					
GO:004	ethanol biosynthetic process involved	9	0.20	5 0.0	0.0051502 9	ADH5

[illegible]34558	[illegible]in glucose fermentation to ethanol		[illegible]%	[illegible]0%	[illegible]88915322	[illegible]
GO:0000017	alpha-glucoside transport	6	0.20%	50.00%	0.005150298891532	MAL31
GO:00[illegible]	glutathione transme[illegible]	6	0.2[illegible]	50.0[illegible]	0.00515 02[illegible]	[illegible]

34775	mbrane transport	[illegible]	0%	0%	98891532	GEX1
GO:0044182	filamentous growth of a population of unicellular organisms	4	0.20%	50.00%	0.005150298891532	FIG2
					0.	

GO:0006282	regulation of DNA repair	7	0.20%	50.00%	005150298891532	RPN4
GO:0043388	positive regulation of DNA binding	6	0.20%	50.00%	0.005150298891532	PHO2
GO:0[illegible]	positive regulation of ph[illegible]	[illegible]	0[illegible]	5[illegible]	0.00515[illegible]	

0045937	hosphate metabolic process	8	0.20%	0.00%	0298891532	PHO2
GO:0033859	furaldehyde metabolic process	5	0.20%	50.00%	0.0051502988891532	SFA1
GO:0046294	formaldehyde catabolic	6	0.20%	50.00%	0.0051502988891	SFA1

	process				532	
GO:0045980	negative regulation of nucleotide metabolic process	8	0.20%	50.00%	0.005150298891532	INH1
GO:00	negative regulation of conj	7	0.2	50.0	0.0051502	

311138	ugation with cellular fusion	7	0%	00%	98891532	PRR2
GO:0046835	carbohydrate phosphorylation	5	0.20%	50.00%	0.005150298891532	GAL3
GO:0010992	ubiquitin homeostasis	5	0.20%	50.00%	0.00515029889153	DOA4

					2	
[GO:0071071](#)	regulation of phospholipid biosynthetic process	7	0.20%	50.00%	0.005150298891532	[GIS1](#)
[GO:0015723](#)	bilirubin transport	5	0.20%	50.00%	0.005150298891532	[YCF1](#)
[G](#)	leuc				0.005	

GO:0006552	ine catabolic process	7	0.20%	50.00%	[illegible]150298891532	ARO10
GO:0006572	tyrosine catabolic process	7	0.20%	50.00%	0.005150298891532	ARO10
GO:0006481	C-terminal protein methylation	6	0.20%	50.00%	0.005150298891532	PPM1
	cellu[illegible]				0.[illegible]	

GO:0043623	[illegible]lar protein complex assembly	6	0.20%	50.00%	[illegible]005150298891532	PPM1
GO:0009826	unidimensional cell growth	4	0.20%	50.00%	0.005150298891532	DOT6
GO:0051026	chiasma assembly	4	0.20%	50.00%	0.00515029889153[illegible]	MSH4

					2	
GO:0019588	anaerobic glycerol catabolic process	8	0.20%	50.00%	0.005150298891532	DAK2
GO:0032445	fructose import	8	0.20%	50.00%	0.005150298891532	HXK1
GO:000	matin	3	0.20	50.00	0.0051502 9	MF(ALPHA)2

7618	g		%	%	8891532	
GO:0019953	sexual reproduction	3	0.20%	50.00%	0.0051502988891532	MF(ALPHA)2
GO:0071266	'de novo' L-methionine biosynthetic process	9	0.20%	50.00%	0.0051502988891532	STR3

GO:0035965	cardiolipin acyl-chain remodeling	9	0.20%	50.00%	0.005150298891532	CLD1
GO:0080058	protein deglutathionylation	7	0.20%	50.00%	0.005150298891532	TRX2
GO:0015768	maltose transport	7	0.20%	50.00%	0.0051502988915[illegible]	MAL11

					[illegible]32	
GO:0005981	regulation of glycogen catabolic process	7	0.20%	50.00%	0.005150298891532	PCL7
GO:0006591	ornithine metabolic process	6	0.20%	50.00%	0.005150298891532	ARG3
GO[illegible]	response to [illegible]				0.0051[illegible]	

:0042542	to hydrogen peroxide	5	0.20%	50.00%	50298891532	GSH1
GO:0001320	age-dependent response to reactive oxygen species involved in ch	7	0.20%	50.00%	0.005150298891532	SOD1

	chronological cell aging					
[GO:0051091](GO:0051091)	positive regulation of sequence-specific DNA binding transcri	5	0.20%	50.00%	0.005150298891532	[SOD1](SOD1)

	ption factor activity					
[GO:0009081]()	branched-chain amino acid metabolic process	5	0.20%	50.00%	0.005150298891532	[BAT2]()
[GO:004825]()	mitochondrial iron ion tr	5	0.20%	50.00%	0.005150298889	[MRS4]()

[illegible]0	[illegible]ansport				[illegible]1532	
GO:0042938	dipeptide transport	7	0.20%	50.00%	0.005150298891532	PTR2
GO:0031954	positive regulation of protein autophosphoryl	10	0.20%	50.00%	0.005150298891532	SSK1

	ation					
GO:0006110	regulation of glycolysis	7	0.20%	50.00%	0.00515029889153 2	YLR345W
GO:0090155	negative regulation of sphingolipid biosynthetic process	7	0.20%	50.00%	0.00515029889153 2	ORM2

	ess					
GO:0090156	cellular sphingolipid homeostasis	6	0.20%	50.00%	0.005150298891532	ORM2
GO:0046713	borate transport	6	0.20%	50.00%	0.005150298891532	ATR1
GO:0	transcription from		0	5	0.00515	

[illegible]0006390	[illegible]m mitochondrial promoter	6	0.20%	0.00%	[illegible]0298891532	RSF1
GO:0070485	dehydro-D-arabinono-1,4-lactone biosynthetic process	7	0.20%	50.00%	0.005150298891532	ARA2
	beta-al[illegible]				0.00[illegible]	

GO:0019483	anine biosynthetic process	6	0.20%	50.00%	5150298891532	ALD3
GO:0044070	regulation of anion transport	7	0.20%	50.00%	0.005150298891532	POR1
GO:0051027	DNA transport	7	0.20%	50.00%	0.00515029889153	POR1

					2	
GO:0015718	monocarboxylic acid transport	6	0.20%	50.00%	0.005150298891532	AQR1
GO:0009092	homoserine metabolic process	6	0.20%	50.00%	0.005150298891532	MET2
GO:00060[illegible]	inositol metabolic [illegible]	5	0.20%	50.00%	0.00515029888[illegible]	OPI10

20	process				91532	
GO:0031503	protein complex localization	5	0.20%	50.00%	0.005150298891532	ATG34
GO:0008204	ergosterol metabolic process	5	0.20%	50.00%	0.005150298891532	GRE2
GO:004	hyperosmotic salin	6	0.20	50.00	0.00515029	CIN5

2538	ity response		%	%	88915332	
GO:0043148	mitotic spindle stabilization	5	0.20%	50.00%	0.005150298891532	ASE1
GO:007	adenylate cyclase-modulating G-protein co	8	0.20%	50.00%	0.00515029 8	RGS2

[188](#)	coupled receptor signaling pathway			%	891532	
[GO:0006675](#)	mannosyl-inositol phosphorylceramide metabolic	7	0.20%	50.00%	0.005150298891532	[SUR1](#)

	c process					
[GO:0045016]	mitochondrial magnesium ion transport	5	0.20%	50.00%	0.005150298891532	[MFM1]
[GO:0006672]	ceramide metabolic process	6	0.20%	50.00%	0.005150298891532	[YDC1]
G	cerami				0.005	

GO:0046514	[illegible]mide catabolic process	7	0.20%	50.00%	[illegible]150298891532	YDC1
GO:0001933	negative regulation of protein phosphorylation	9	0.20%	50.00%	0.005150298891532	NCE102
GO:00[illegible]	cellular resp[illegible]	4[illegible]	0.2[illegible]	50.0[illegible]	0.005150 2[illegible]	

33554	onse to stress	4	0%	00%	98891532	CUR1
GO:0015700	arsenite transport	7	0.20%	50.00%	0.005150298891532	ARR3
GO:0000947	amino acid catabolic process to alcohol via E	7	0.39%	33.33%	0.00625337733327	ADH5 SFA1

	Ehrlich pathway				7	
GO:0051598	meiotic recombination checkpoint	5	0.39%	33.33%	0.00625337733327	PCH2 MEK1
GO:0051260	protein homooligomerization	7	0.39%	33.33%	0.00625337733327	YDR132C STE2
G	re				0.00	

GO:0010038	sponse to metal ion	5	0.39%	33.33%	6253377333277	YCF1 AHP1
GO:0019430	removal of superoxide radicals	6	0.39%	33.33%	0.006253377333277	TRR1 SOD1
GO:0006000	fructose metabolic process	8	0.39%	33.33%	0.006253377333277	HXK1 YLR345W
					0.	

GO:0009099	valine biosynthetic process	7	0.39%	33.33%	006253377333277	BAT2 ILV5
GO:0042138	meiotic DNA double-strand break formation	4	0.59%	25.00%	0.008181633587576	PCH2 SAE2 REC104
GO:0	copper io		0.	2	0.00818181	

0068825	n transport	8	59%	5.00%	6335875776	FRE2 FRE1 FRE7
GO:0019236	response to pheromone	5	1.18%	17.65%	0.009280291221183	FIG2 PRR2 STE2 BAR1 STE18 PRM1
GO:0005992	trehalose biosynthetic process	7	0.39%	28.57%	0.010363602271737	TPS2 TPS3
	G-protein				0.	

GO:0007186	coupled receptor signaling pathway	6	0.39%	28.57%	0.010363602271737	STE2 STE18
GO:0070941	eisosome assembly	5	0.39%	28.57%	0.010363602271737	SLM1 NCE102
GO:0019[illegible]	calcium-mediate[illegible]	7	0.39%	28.57[illegible]	0.01036360[illegible]	RSF1 BXI1

[illegible]722	[illegible]d signaling		[illegible]%	[illegible]%	[illegible]2717737	
GO:0005978	glycogen biosynthetic process	8	0.59%	23.08%	0.011157359586505	GLC3 GSY1 IGD1
GO:0030447	filamentous growth	3	0.59%	23.08%	0.011157359586505	DOT6 MGA1 GRE2
GO:00[illegible]	fungal-type ce[illegible]	[illegible]	2.3[illegible]	13.[illegible]	0.0126146[illegible]	[illegible]

[illegible]31505	[illegible]ll wall organization	5	[illegible]5%	[illegible]19%	[illegible]84870249	SPS22 PST1 MTL1 SOD1 PIR3 PIR1 YLR194C LST8 HRD1 ZEO1 SRL1 CSR2
GO:0006241	CTP biosynthetic process	9	0.20%	33.33%	0.014713688492883	URA7
GO:0019856	pyrimidine nucleobase biosynthetic proc[illegible]	7	0.20%	33.33%	0.014713688492883	URA7

	...es s					
GO:1900460	negative regulation of invasive growth in response to glucose limitation by negative regul...	1 0	0. 2 0 %	3 3. 3 3 %	0. 0 1 4 7 1 3 6 8 8 4 9 2 8 8...	NRG2

	ulation of transcription from RNA polymerase II promoter				3	
[GO:003](#)	basic amino acid transmembr	7	0.20%	33.33	0.01471368	[RTC2](#)

[GO:...4488]()	...ane export from vacuole		...%	...%	...8492883	
[GO:0090180]()	positive regulation of thiamine biosynthetic process	7	0.20%	33.33%	0.0147136884928 83	[THI2]()
[GO:0...]()	threonine ...		0....	3...	0.013...	

[illegible]0065667	[illegible]catabolic process	7	[illegible]20%	[illegible]3.33%	[illegible]688492883	CHA1
GO:0019676	ammonia assimilation cycle	7	0.20%	33.33%	0.014713688492883	GLT1
GO:0043335	protein unfolding	6	0.20%	33.33%	0.014713688492883	HSP78
GO[illegible]	glycine[illegible]	[illegible]	[illegible]	[illegible]	0.0147[illegible]	[illegible]

[illegible]:0006545	[illegible] biosynthetic process	7	0.20%	33.33%	[illegible]13688492883	AGX1
GO:0046323	glucose import	8	0.20%	33.33%	0.014713688492883	HXK1
GO:[illegible]	gene conversion at mating-type lo[illegible]			3[illegible]	0.01471[illegible]	

0031292	cus, DNA double-strand break processing	4	0.20%	3.33%	3688492883	SAE2
GO:0043171	peptide catabolic process	5	0.20%	33.33%	0.014713688492883	BAR1
GO:00	regulation of		0.2	33.	0.0147136	

031647	protein stability	4	20%	33%	0884928883	PCL7
GO:0016197	endosomal transport	4	0.20%	33.33%	0.0147136884928883	SLM1
	positive regulation of gluconeogenesis					

GO:0035948	by positive regulation of transcription from RNA polymerase II promoter	9	0.20%	33.33%	0.014713688492883	SIP4
GO:	positive regulatio				0.01471	

-0045722	n of gluconeogenesis	8	0.20%	33.33%	3688492883	SIP4
GO:0061414	positive regulation of transcription from RNA polymerase II pro	9	0.20%	33.33%	0.01471368849288	SIP4

	[illegible]moter by a nonfermentable carbon source				[illegible]83	
GO:0006119	oxidative phosphorylation	5	0.20%	33.33%	0.0147136884928883	GSM1
GO:[illegible]	cellular respons[illegible]			3[illegible]	0.01471[illegible]	

0042631	e to water deprivation	5	0.20%	3.33%	368849288 3	YJL144W
GO:0000751	cell cycle arrest in response to pheromone	4	0.20%	33.33%	0.014713688492883	FAR1
GO:003	heterotrimeric G-pr	7	0.20	33.3	0.01471368	STE18

1684	otein complex cycle		%	3%	8492883	
GO:0006556	S-adenosylmethionine biosynthetic process	6	0.20%	33.33%	0.014713688492883	SAM1
GO:	transcription initiation fro			[illegible]	0.01471	

0006361	m RNA polymerase I promoter	8	0.20%	33.33%	3688492883	[RRN11]()
[GO:0006089]()	lactate metabolic process	5	0.20%	33.33%	0.014713688492883	[CYB2]()
[GO:0007050]()	cell cycle arrest	5	0.20%	33.33%	0.014713688492	[HUG1]()

					883	
GO:0001682	tRNA 5'-leader removal	9	0.20%	33.33%	0.014713688492883	RPM2
GO:0000281	cytokinesis after mitosis	6	0.20%	33.33%	0.014713688492883	HOF1
GO:00065[illegible]	polyamine catabolic [illegible]	6	0.20%	33.33%	0.0147136884[illegible]	ALD3

98	process				92883	
GO:0006820	anion transport	5	0.20%	33.33%	0.0147136884928 83	POR1
GO:0046496	nicotinamide nucleotide metabolic process	7	0.20%	33.33%	0.0147136884928 83	YNL200C
	met[illegible]					

GO:0019243	nylglyoxal catabolic process to D-lactate	6	0.20%	33.33%	0.01471368849288 3	GLO4
GO:0038032	termination of G-protein coupled receptor signali	8	0.20%	33.33%	0.01471368849288 3	RGS2

	ng pathway					
[GO:0006688]	glycosphingolipid biosynthetic process	7	0.20%	33.33%	0.0147136884928883	[SUR1]
[GO:0007026]	negative regulation of microtubule d	7	0.20%	33.33%	0.014713688492	[KIP2]

[illegible]	depolymerization				2883	
GO:0035955	negative regulation of dipeptide transport by negative regulation of	8	0.20%	33.33%	0.014713688492	CUP9

	[illegible] transcription from RNA polymerase II promoter				883	
GO:0008360	regulation of cell shape	4	0.59%	21.43%	0.014749602938930	FIG2 GIC2 FKS3
G	tri				0.015	

O:0006099	carboxylic acid cycle	4	0.98%	17.24%	301403604094	ACO2 SHH4 IDP3 MLS1 IDH2
GO:0000753	cell morphogenesis involved in conjugation with cellular fusion	4	0.39%	25.00%	0.015706604953618	FIG1 FIG2

	..					
GO:0007155	cell adhesion	3	0.39%	25.00%	0.0157066049536618	FIG2 HSP12
GO:0006012	galactose metabolic process	8	0.39%	25.00%	0.0157066049536618	GAL3 GAL4
GO:0015758	glucose transport	7	0.39%	25.00%	0.0157066049536[illegible]	MTH1 HXT5

					18	
GO:0007130	synaptonemal complex assembly	5	0.39%	25.00%	0.0157066049536 18	ZIP2 SPO16
GO:0000114	regulation of transcription involved in G1 phase	5	0.39%	25.00%	0.0157066049536 18	MSA2 MSA1

	of mitotic cell cycle					
GO:0006097	glyoxylate cycle	5	0.39%	25.00%	0.01570660495361 8	IDP3 MLS1
GO:0016310	phosphorylation	6	4.31%	10.73%	0.02120959713318 1	RTK1 MRK1 PRR2 VHS1 SPS1 ARG5,6 DAK2 CMK1 HXK1 TOS3 FMP48 TDA10 IKS1 LSB6 IME2 YAK1YLR345W YLR446W RIM11 HRK1 MEK1 TPK2
GO	alcohol				0.0223	

[:0006066]()	metabolic process	5	0.39%	22.22%	21320625772	[ADH7]() [YPL272C]()
[GO:0034605]()	cellular response to heat	5	0.39%	22.22%	0.022321320625772	[HSP78]() [HSP12]()
[GO:0009083]()	branched-chain amino acid catabolic	6	0.39%	22.22%	0.022321320625772	[ARO10]() [BAT2]()

	c process				2	
GO:0019363	pyridine nucleotide biosynthetic process	7	0.39%	22.22%	0.022321320625772	PNC1 NMA2
GO:0046685	response to arsenic-containing substance	4	0.39%	22.22%	0.022321320625772	ARR2 ARR3

GO:0006915	apoptotic process	6	0.78%	16.67%	0.025469924348326	DRE2 POR1 BXI1 OYE3
GO:0000501	flocculation via cell wall protein-carbohydrate interaction	5	0.20%	25.00%	0.028031958342814	FLO9
	reg[illegible]					

GO:0043666	ulation of phosphoprotein phosphatase activity	7	0.20%	25.00%	0.028031958342814	GIP1
GO:0016036	cellular response to phosphate starvatio	6	0.20%	25.00%	0.028031958342814	PHO5

	n					
GO:0009410	response to xenobiotic stimulus	4	0.20%	25.00%	0.028031958342814	RDS1
GO:0042176	regulation of protein catabolic process	6	0.20%	25.00%	0.028031958342814	MRK1
GO:0	glucose catabolic		0.	25.	0.02803 1	

[GO:...019655]()	...e process to ethanol	8	...20%	...00%	...958342814	[NDE2]()
[GO:0032878]()	regulation of establishment or maintenance of cell polarity	5	0.20%	25.00%	0.028031958342814	[PCL9]()
[GO:...]()	regulation...			2...	0.02803...	

[illegible]0006275	[illegible] of DNA replication	7	0.20%	25.00%	[illegible]1958342814	DOA4
GO:0016577	histone demethylation	6	0.20%	25.00%	0.028031958342814	GIS1
GO:000[illegible]	aromatic amino acid family catabolic proc[illegible]	7	0.20[illegible]	25.0[illegible]	0.0280319[illegible]	ARO10

0949	ocess to alcohol via Ehrlich pathway		%	0%	8342814	
GO:2000221	negative regulation of pseudohyphal growth	7	0.20%	25.00%	0.028031958342814	DIG2
	ornithi				0.02	

GO:0006592	[illegible]ne biosynthetic process	7	0.20%	25.00%	[illegible]8031958342814	ARG5,6
GO:0009070	serine family amino acid biosynthetic process	6	0.20%	25.00%	0.028031958342814	SER3
GO:0006[illegible]	mannose metaboli[illegible]	8	0.20%	25.00%	0.02803195[illegible]	HXK1

013	olic process				342814	
[GO:0019358]()	nicotinate nucleotide salvage	8	0.20%	25.00%	0.028031958342814	[PNC1]()
[GO:0000729]()	DNA double-strand break processing	8	0.20%	25.00%	0.028031958342814	[SAE2]()
G					0.02	

GO:0019346	transsulfuration	7	0.20%	25.00%	80319583428814	STR3
GO:0032048	cardiolipin metabolic process	8	0.20%	25.00%	0.0280319583428814	CLD1
GO:0006850	mitochondrial pyruvate transport	5	0.20%	25.00%	0.0280319583428814	FMP43

	GO:1900461	positive regulation of pseudohyphal growth by positive regulation of transcription from RN	8	0.20%	25.00%	0.028031958342814	ASH1

	NA polymerase II promoter					
GO:0090338	positive regulation of formin-nucleated actin cable assembly	8	0.20%	25.00%	0.028031958342814	RHO4
	os					

GO:0007234	osmosensory signaling via phosphorelay pathway	6	0.20%	25.00%	0.0280319583428142814	SSK1
GO:0006003	fructose 2,6-bisphosphate metabolic	5	0.20%	25.00%	0.0280319583428142814	YLR345W

	process					
GO:0010466	negative regulation of peptidase activity	7	0.20%	25.00%	0.02803195834281 4	PAI3
GO:0031385	regulation of termination of mating proj[illegible]	6	0.20%	25.00%	0.02803195834281[illegible]	FUS2

	ection growth				4	
GO:0006075	(1->3)-beta-D-glucan biosynthetic process	9	0.20%	25.00%	0.0280319583428814	FKS3
GO:0015680	intracellular copper ion transpor	5	0.20%	25.00%	0.0280319583428814	FET4

	t					
GO:0031591	wybutosine biosynthetic process	8	0.20%	25.00%	0.028031958342814	PPM2
GO:0000073	spindle pole body separation	5	0.20%	25.00%	0.028031958342814	ASE1
GO:0015848	spermidine transpor	5	0.20%	25.00%	0.028031958342	TPO4

[illegible]	[illegible]t				[illegible]814	
GO:0016071	mRNA metabolic process	6	0.20%	25.00%	0.028031958342814	AEP3
GO:0015693	magnesium ion transport	8	0.20%	25.00%	0.028031958342814	MFM1
GO:00157[illegible]	glycerol transp[illegible]	5	0.20%	25.00%	0.0280319583[illegible]	GUP2

[illegible]93	[illegible]ort				[illegible]42814	
GO:0006468	protein phosphorylation	7	2.94%	11.28%	0.028279265533584	RTK1 MRK1 PRR2 VHS1 SPS1 CMK1 TOS3 FMP48 IKS1 IME2 YAK1 RIM11 HRK1 MEK1 TPK2
GO:0006334	nucleosome assembly	6	0.59%	17.65%	0.029554713553893	HTB2 HTA2 HHT2
GO[illegible]	ribosomal large[illegible]				0.0300[illegible]	

[illegible]:0000055	[illegible]e subunit export from nucleus	6	0.78%	16.00%	[illegible]173686653 89	ARX1 RIX7 NOG2 RRS1
GO:0006116	NADH oxidation	5	0.39%	20.00%	0.030217881495066	ADH5 NDE2
GO:0046686	response to cadmium ion	6	0.39%	20.00%	0.03021788149506	YCF1 GSH1

					6	
GO:0006616	SRP-dependent cotranslational protein targeting to membrane, translocation	7	0.39%	20.00%	0.0302178814950 66	SSA4 WSC4
	resp[illegible]				0.03[illegible]	

GO:0006986	onse to unfolded protein	5	0.39%	20.00%	30217881495066	ULI1 ORM2
GO:0070086	ubiquitin-dependent endocytosis	4	0.39%	20.00%	0.030217881495066	RIM8 CSR2
GO:009099	isoleucine biosynthetic	7	0.39%	20.00%	0.0302178149	BAT2 ILV5

7	process				5066	
GO:0007039	vacuolar protein catabolic process	7	0.39%	20.00%	0.030217881495066	APE1 PAI3
GO:0030437	ascospore formation	5	1.37%	13.21%	0.033948690006572	SPS1 SPO74 SPO16 SUR7 RIM11 SPS4 YOR338W
GO:0	cellular amino ac		0.	1	0.035044	

006520	idmetabolic process	4	78%	[illegible]5.38%	578687785	CHA1 ARG5,6 SPS100 ARG3
GO:0006333	chromatin assembly or disassembly	7	0.59%	16.67%	0.035904795407749	HTB2 HTA2 HHT2
GO:0006526	arginine biosynthetic process	7	0.39%	18.18%	0.039382736960962	ARG5,6 ARG3
					0.	

GO:0009435	NAD biosynthetic process	9	0.39%	18.18%	039382736960962	NMA2 BNA2
GO:0001558	regulation of cell growth	5	0.39%	18.18%	0.039382736960962	SLM1 LST8
GO:0008[illegible]	attachment of spindle microtub[illegible]	5	0.20%	20.00%	0.044518384[illegible]	AME1

[608](#)	bules to kinetochore				831228	
[GO:0043086](#)	negative regulation of catalytic activity	5	0.20%	20.00%	0.0445183848831228	[RRT12](#)
[GO:0046688](#)	response to copper ion	6	0.20%	20.00%	0.0445183848831228	[YCR102C](#)

GO:0061395	positive regulation of transcription from RNA polymerase II promoter in response to arsenic-	7	0.20%	20.00%	0.04451838483122 8	RPN4

	containing substance					
GO:0000955	amino acid catabolic process via Ehrlich pathway	6	0.20%	20.00%	0.044518384831228	ARO10
GO:00	L-phenylalanin		0.	20.	0.044518	

…0065559	…e catabolic process	7	…20%	…00%	…848312 28	ARO10
GO:0006564	L-serine biosynthetic process	7	0.20%	20.00%	0.04451838384831228	SER3
GO:0019563	glycerol catabolic process	7	0.20%	20.00%	0.04451838384831228	DAK2
G…	cAMP-…				0.044…	

O:0019933	mediated signaling	8	0.20%	20.00%	518384831228	PDE1
GO:0006885	regulation of pH	9	0.20%	20.00%	0.044518384831228	BTN2
GO:0006662	glycerol ether metabolic process	5	0.20%	20.00%	0.044518384831228	TRX2
	p					

GO:0009051	entose-phosphate shunt, oxidative branch	7	0.20%	20.00%	0.0445183848312 28	SOL4
GO:0010520	regulation of reciprocal meiotic recombination	8	0.20%	20.00%	0.0445183848312 28	SPO16

[GO:0034354]()	de novo NAD biosynthetic process from tryptophan	11	0.20%	20.00%	0.044518384831228	[BNA2]()
[GO:0006801]()	superoxide metabolic process	5	0.20%	20.00%	0.044518384831228	[SOD1]()
	lon				0.0	

GO:0015909	g-chain fatty acid transport	6	0.20%	20.00%	44518384831228	FAT3
GO:0006857	oligopeptide transport	6	0.20%	20.00%	0.044518384831228	PTR2
GO:0000160	phosphorelay signal transd	5	0.20%	20.00%	0.044518384831	SSK1

[illegible]	[illegible]uction system				[illegible]228	
GO:0006751	glutathione catabolic process	6	0.20%	20.00%	0.044518384831228	ECM38
GO:0006048	UDP-N-acetylglucosamine biosynthetic process	7	0.20%	20.00%	0.044518384831228	YMR084W

GO:0006740	NADPH regeneration	5	0.20%	20.00%	0.044518384831228	IDP3
GO:0009303	rRNA transcription	7	0.20%	20.00%	0.044518384831228	HHT2
GO:00021	tRNA wobble position ur	9	0.20%	20.00%	0.0445183848	NCS2

[illegible]43	[illegible]idine thiolation				[illegible]31228	
GO:0009636	response to toxic substance	4	0.20%	20.00%	0.044518384831228	YNR064C
GO:0[illegible]	regulation of transcription from RNA pol[illegible]		0[illegible]	2[illegible]	0.044518[illegible]	

[illegible]0043619	[illegible]ymerase II promoter in response to oxidative stress	6	[illegible]20%	0.00%	[illegible]3848312 28	YRR1
GO:0070124	mitochondrial translational initiation	5	0.20%	20.00%	0.044518384831228	AEP3
	protein [illegible]				0.04[illegible]	

GO:0097271	n localization to bud neck	6	0.20%	20.00%	4518384831228	AIM44
GO:0090002	establishment of protein localization to plasma membrane	5	0.20%	20.00%	0.044518384831228	NCE102
	carbo				0.04	

GO:0005975	…hydrate metabolic process	4	2.16%	11.22%	…47240681452502	GAL3 GLC3 HXK1 SOL4 YHR210C PCL7 YLR446W YMR085W YNR071C GAS4 GAL4
GO:0006537	glutamate biosynthetic process	7	0.39%	16.67%	0.049783104752091	GLT1 IDH2
GO:0000054	ribosomal subunit export from…	5	0.39%	16.67%	0.0497831047520…	SSB1 SSB2

	m nucleus				91	
[GO:0030950](GO:0030950)	establishment or maintenance of actin cytoskeleton polarity	6	0.39%	16.67%	0.049783104752091	[SLM1](SLM1) [LST8](LST8)
[GO:000091](GO:000091)	barrier septum asse	6	0.39%	16.67%	0.04978310475	[HOF1](HOF1) [AIM44](AIM44)

[illegible]7	[illegible]mbly				[illegible]2091	
GO:0006541	glutamine metabolic process	6	0.59%	15.00%	0.050782362518053	URA7 GLT1 YMR084W
GO:0008654	phospholipid biosynthetic process	5	0.98%	12.82%	0.057564305793387	URA7 YDR018C PDR16 OPI10 MUM3
GO:00[illegible]	fatty acid biosyn[illegible]	[illegible]	0.5[illegible]	14.[illegible]	0.05930500[illegible]	

[illegible]066333	[illegible]thetic process	6	[illegible]9%	[illegible]29%	[illegible]92887544	OLE1 OAR1 MCT1
GO:0000023	maltose metabolic process	6	0.39%	15.38%	0.061370828301767	MAL31 MAL11
GO:0006312	mitotic recombination	7	0.39%	15.38%	0.061370828301767	IRC4 IRC8
	branched-[illegible]				0.[illegible]	

GO:0009082	[illegible] chain amino acid biosynthetic process	6	0.39%	15.38%	[illegible]061370828301767	BAT2 ILV5
GO:0042710	biofilm formation	4	0.20%	16.67%	0.063650888580204	NRG2
GO:0[illegible]	ascospore-type prosp[illegible]		0[illegible]	1[illegible]	0.06365[illegible]	

032120	ore membrane assembly	4	0.20%	6.67%	888580204	YSW1
GO:0000920	cytokinetic cell separation	5	0.20%	16.67%	0.063650888580204	SDS24
	positive regulation of transcriptio				0.0	

GO:0007329	on from RNA polymerase II promoter by pheromones	9	0.20%	16.67%	063650888580204	KAR4
	positive regulation of transcription				0.0	

GO:0036003	n from RNA polymerase II promoter in response to stress	7	0.20%	16.67%	63650888580204	RPN4
GO:0009113	purine nucleobase biosynthetic	5	0.20%	16.67%	0.06365088858020	PHO2

	process				4	
[GO:0009263]	deoxyribonucleotide biosynthetic process	6	0.20%	16.67%	0.0636508885802004	[TRX2]
[GO:0001100]	negative regulation of exit from mitosis	7	0.20%	16.67%	0.0636508885802004	[KEL2]

GO:0006363	termination of RNA polymerase I transcription	8	0.20%	16.67%	0.063650888580204	RPA12
GO:0071805	potassium ion transmembrane tra	5	0.20%	16.67%	0.063650888580204	PRM6

	nsport					
GO:0000075	cell cycle checkpoint	8	0.20%	16.67%	0.0636508885802 04	HUG1
GO:0031930	mitochondria-nucleus signaling pathway	5	0.20%	16.67%	0.0636508885802 04	LST8
	am				0.06	

GO:0015696	[illegible]monium transport	5	0.20%	16.67%	[illegible]3650888580204	ATO2
GO:0010388	cullin deneddylation	10	0.20%	16.67%	0.06365088858020 4	RRI2
GO:0046513	ceramide biosynthetic process	7	0.20%	16.67%	0.06365088858020 4	YDC1
	e[illegible]					

GO:0000480	ndonucleolytic cleavage in 5'-ETS of tricistronic rRNA transcript (SSU-rRNA, 5.8S rRNA,	8	0.78%	12.90%	0.067629140066866	UTP20 UTP10 UTP13 RCL1

	LSU-rRNA)					
GO:0006139	nucleobase-containing compound metabolic process	4	0.59%	13.64%	0.068537549143858	KAR4 IME4 SRL1
GO:00162	iron-sulfur cluster	4	0.59%	13.64%	0.0685375491	IBA57 DRE2 ISU2

26	assembly				43858	
GO:0030968	endoplasmic reticulum unfolded protein response	6	0.39%	14.29%	0.074085707946177	ULI1 BXI1
	adaptation of signalin					

GO:0000754	g pathway by response to pheromone involved in conjugation with cellular fusion	5	0.39%	14.29%	0.0740857079461777	BAR1 RRI2
	Raspr				0.07	

GO:0007265	otein signal transduction	7	0.39%	14.29%	74085707946177	RPI1 TPK2
GO:0000128	flocculation	4	0.20%	14.29%	0.084965803103591	FLO9
GO:0	peptidyl-diphthamide biosynth		0	1	0.084965	

0017183	thetic process from peptidyl-histidine	5	0.20%	4.29%	5803103591	KTI11
GO:0009987	cellular process	2	0.20%	14.29%	0.084965803103591	ARX1
GO:00508	protein stabilizati	5	0.20%	14.29%	0.0849658031	HSP78

21	ation				03591	
GO:0043328	protein targeting to vacuole involved in ubiquitin-dependent protein catabolic	6	0.20%	14.29%	0.084965803103590	GGA1

	[illegible]c process via the multivesicular body sorting pathway				[illegible]1	
[GO:0006569]()	tryptophan catabolic process	7	0.20%	14.29%	0.084965803103591	[ARO10]()
	rib[illegible]				0.0[illegible]	

GO:0009231	oflavin biosynthetic process	7	0.20%	14.29%	84965803103591	RIB3
GO:0000706	meiotic DNA double-strand break processing	4	0.20%	14.29%	0.084965803103591	SAE2
GO:00	regulation of		0.	14.	0.0849658	

[illegible]07346	mitotic cell cycle	6	[illegible]20%	[illegible]29%	[illegible]03103591	ZPR1
GO:0009267	cellular response to starvation	5	0.20%	14.29%	0.0849658031035 91	ZPR1
GO:0046352	disaccharide catabolic process	6	0.20%	14.29%	0.0849658031035 91	MAL11
GO:[illegible]	allantoin[illegible]	[illegible]	[illegible]	[illegible]	0.08496[illegible]	[illegible]

:0000256	n catabolic process	6	0.20%	14.29%	05803103591	DAL1
GO:0040020	regulation of meiosis	6	0.20%	14.29%	0.084965803103591	IME2
GO:0006469	negative regulation of protein kinase	8	0.20%	14.29%	0.084965803103591	FAR1

	activity					
GO:0009098	leucine biosynthetic process	7	0.20%	14.29%	0.084965803103591	BAT2
GO:0006555	methionine metabolic process	6	0.20%	14.29%	0.084965803103591	SAM1
GO:	regulation of Rho				0.08496	

:0035023	protein signal transduction	8	0.20%	14.29%	65803103591	FUS2
GO:0070911	global genome nucleotide-excision repair	8	0.20%	14.29%	0.084965803103591	HHT2
GO:0	protein		0	1	0.0849665	

[illegible]0032447	[illegible]n urmylation	9	0.20%	[illegible]4.29%	[illegible]5803103591	NCS2
GO:0000096	sulfur amino acid metabolic process	5	0.20%	14.29%	0.084965803103591	MET2
GO:0048278	vesicle docking	5	0.20%	14.29%	0.084965803103591	MPC54
	mi[illegible]				0.[illegible]	

GO:0007018	crotubule-based movement	5	0.20%	14.29%	08496580310359 1	KIP2
GO:0003333	amino acid transmembrane transport	6	0.59%	12.50%	0.08906224843436 5	MUP3 MMP1 DIP5
	endonucleolyti					

GO:0000472	c cleavage to generate mature 5'-end of SSU-rRNA from (SSU-rRNA, 5.8S rRNA, LSU-	8	0.78%	11.76%	0.093142332434696	UTP20 UTP10 UTP13 RCL1

	rRNA)					
GO:000	endonucleolytic cleavage in ITS1 to separate SSU-rRNA from 5.8S rRNA and	8	0.98%	11.11	0.10071526	[UTP20](#) [UTP10](#) [UTP13](#) [RCL1](#) [RRS1](#)

0447	LSU-rRNA from tricistronic rRNA transcript (SSU-rRNA, 5.8S rRNA, LSU-rRNA)		%	%	0648224	
GO:	prot				0.10261	

[illegible]0042026	[illegible]ein refolding	7	0.39%	12.50%	[illegible]2703667840	HSP78 CPR6
GO:0000747	conjugation with cellular fusion	4	0.39%	12.50%	0.102612703667840	KEL2 ASG7
GO:0005977	glycogen metabolic process	7	0.39%	12.50%	0.102612703667840	AAP1 PCL7
	phos[illegible]					

GO:0006796	phate-containing compound metabolic process	5	0.20%	12.50%	0.108052202486915	PHO5
GO:0034501	protein localization to kinetochore	8	0.20%	12.50%	0.108052202486915	AME1
					0.	

GO:0000741	karyogamy	6	0.20%	12.50%	1080522024869 15	KAR4
GO:0000083	regulation of transcription involved in G1/S phase of mitotic ce	5	0.20%	12.50%	0.108052202486915	PCL9

	ll cycle					
GO:0019752	carboxylic acid metabolic process	4	0.20%	12.50%	0.108052202486915	LYS20
GO:0045143	homologous chromosome segregation	5	0.20%	12.50%	0.108052202486915	MSH4
					0.10	

GO:0000103	sulfate assimilation	5	0.20%	12.50%	8052202486915	TRX2
GO:0051017	actin filament bundle assembly	5	0.20%	12.50%	0.108052202486915	SLM1
GO:0000	re-entry into mitotic cell cycle af	6	0.20%	12.50	0.1080522022	POG1

321	ter pheromone arrest			%	486915	
GO:0006144	purine nucleobase metabolic process	6	0.20%	12.50%	0.108052202486915	DAL1
GO:004[illegible]	phosphatidylinositol de[illegible]	7	0.20[illegible]	12.50[illegible]	0.10805220[illegible]	TEP1

6856	phosphorylation		%	%	2486915	
GO:0019740	nitrogen utilization	3	0.20%	12.50%	0.108052202486915	ATO2
GO:0000226	microtubule cytoskeleton organizatio	5	0.20%	12.50%	0.108052202486915	ASE1

	n					
GO:0043618	regulation of transcription from RNA polymerase II promoter in response to stress	6	0.20%	12.50%	0.10805220248691 5	ROX1
					0.10[illegible]	

GO:0009306	protein secretion	5	0.20%	12.50%	[illegible]8052202486915	NCE102
GO:0006450	regulation of translational fidelity	4	0.39%	11.76%	0.118267987263686	SSB1 SSB2
GO:0031929	TOR signaling cascade	6	0.39%	11.76%	0.118267987263686	SLM1 LST8
	tr[illegible]					

[GO:0042790]()	transcription of nuclear large rRNA transcript from RNA polymerase I promoter	8	0.39%	11.76%	0.118267987263686	[RPA12]() [RRN11]()
[GO:]()	sign				0.12738	

:0007165	al transduction	4	1.37%	10.00%	8995880094	COS111 MTH1 STE2 CMK1 STE18 LST8 BAG7
GO:0000080	G1 phase of mitotic cell cycle	7	0.20%	11.11%	0.132546740161527	KAR4
GO:0009085	lysine biosynthetic process	7	0.20%	11.11%	0.132546740161527	LYS20
	lysin					

[GO:0019878]	e biosynthetic process via aminoadipic acid	8	0.20%	11.11%	0.132546740161527	[LYS20]
[GO:0007010]	cytoskeleton organization	5	0.20%	11.11%	0.132546740161527	[HSP42]
[GO:0	meiotic chromos		0.	1 1	0.13254 6	

[illegible]045132	[illegible]ome segregation	4	[illegible]20%	[illegible]1.11%	[illegible]740161527	MAM1
GO:0006278	RNA-dependent DNA replication	7	0.20%	11.11%	0.132546740161527	YIL082W-A
GO:0031578	mitotic spindle orientation checkpoi[illegible]	8	0.20%	11.11%	0.132546740161527	BFA1

	nt					
GO:0019509	L-methionine salvage from methylthioadenosine	7	0.20%	11.11%	0.132546740161527	BAT2
GO:0006189	'de novo' IMP biosynthetic process	10	0.20%	11.11%	0.132546740161527	ADE17

GO:0006829	zinc ion transport	8	0.20%	11.11%	0.132546740161527	FET4
GO:0006972	hyperosmotic response	5	0.20%	11.11%	0.132546740161527	CIN5
GO:0007096	regulation of exit from mito[illegible]	7	0.39%	11.11%	0.1347406256348[illegible]	GIC2 BFA1

	[illegible]sis				[illegible]73	
[GO:0000011]	vacuole inheritance	6	0.39%	11.11%	0.134740625634873	[TRX2] [PEP12]
[GO:0007131]	reciprocal meiotic recombination	4	0.98%	10.20%	0.136778879131885	[PCH2] [MSH4] [ZIP2] [REC104] [MLH2]
[GO:00[illegible]	pyrimidine nucleotid[illegible]	[illegible]	0.2[illegible]	10.[illegible]	0.1581289[illegible]	

06221	e biosynthetic process	7	0%	00%	5482814 2	URA7
GO:0000433	negative regulation of transcription from RNA polymerase II prom	9	0.20%	10.00%	0.158128954828142	NRG2

	[illegible]oter by glucose					
GO:0000105	histidine biosynthetic process	6	0.20%	10.00%	0.158128954828142	PHO2
GO:0006817	phosphate ion transport	7	0.20%	10.00%	0.158128954828142	PIC2
GO:0[illegible]	glycerol met[illegible]		0[illegible]	1[illegible]	0.158128[illegible]	

[illegible]0006071	[illegible]etabolic process	6	[illegible]0.20%	[illegible]0.00%	[illegible]8954828142	DAK2
GO:0007129	synapsis	5	0.20%	10.00%	0.158128954828142	ZIP2
GO:0009102	biotin biosynthetic process	6	0.20%	10.00%	0.158128954828142	BIO2
GO[illegible]	cellular zi[illegible]				0.1581[illegible]	

[GO:0006882]	zinc ion homeostasis	9	0.20%	10.00%	[illegible]128954828142	[SOD1]
[GO:0033615]	mitochondrial proton-transporting ATP synthase complex assembly	6	0.20%	10.00%	0.158128954828142	[ATP10]

GO:0000022	mitotic spindle elongation	6	0.20%	10.00%	0.158128954828142	ASE1
GO:0006814	sodium ion transport	7	0.20%	10.00%	0.158128954828142	ARR3
GO:000865[illegible]	cellular amino acid biosynth[illegible]	5	1.76%	9.18%	0.16443786850[illegible]	GLT1 LYS20 ARG5,6 SER3 STR3 ARG3 BAT2 ILV5 MET2

[illegible]2	[illegible]etic process				[illegible]9204	
GO:0006865	amino acid transport	5	0.78%	9.76%	0.168373633880968	BTN2 MUP3 MMP1 DIP5
GO:0000082	G1/S transition of mitotic cell cycle	7	0.59%	9.68%	0.179626067897962	VHS1 CDC6 MSA1
G[illegible]	early end[illegible]	[illegible]	[illegible]	[illegible]	0.184[illegible]	

[illegible]O:0034498	[illegible]osome to Golgi transport	5	0.20%	9.09%	[illegible]517003243071	ATG20
GO:0006813	potassium ion transport	7	0.20%	9.09%	0.184517003243071	YDL183C
GO:0000710	meiotic mismatch repair	4	0.20%	9.09%	0.184517003243071	MSH4

GO:0032851	positive regulation of Rab GTPase activity	9	0.20%	9.09%	0.18451700324307 1	OCA5
GO:0031938	regulation of chromatin silencing at telomere	7	0.20%	9.09%	0.18451700324307 1	CDC6
	cyto[illegible]				[illegible]	

GO:0007109	[illegible]kinesis, completion of separation	5	0.20%	9.09%	0.184517003243071	ASE1
GO:0030150	protein import into mitochondrial matrix	5	0.39%	9.52%	0.188213490990987	HSP78 MDJ2
GO[illegible]	invasive growth in [illegible]				0.2061[illegible]	

:0001403	response to glucose limitation	7	0.78%	9.09%	80883610759	FIG2 RIM8 NCS2 TPK2
GO:0006006	glucose metabolic process	8	0.39%	9.09%	0.207109692335271	HXK1 TOS3
GO	positive regulation of transcripti				0.21146	

:0045943	ption from RNA polymerase I promoter	9	0.20%	8.33%	[illegible]3782941267	UTP10
GO:0006878	cellular copper ion homeostasis	8	0.20%	8.33%	0.211463782941267	SOD1
GO:00[illegible]	protein su[illegible]		0.2[illegible]	8.3[illegible]	0.2114637[illegible]	

[illegible]016925	[illegible]sumoylation	9	[illegible]20%	[illegible]33%	[illegible]82941267	APJ1
GO:0009060	aerobic respiration	7	0.98%	8.77%	0.223569184126678	RIB3 OAR1 RSF1 ISF1 MCT1
GO:0002098	tRNA wobble uridine modification	9	0.39%	8.70%	0.226406617022557	KTI11 ISU2
	chro[illegible]				0.22[illegible]	

GO:0000183	matin silencing at rDNA	6	0.39%	8.70%	6406617022557	DOT6 PNC1
GO:0006298	mismatch repair	7	0.39%	8.70%	0.226406617022557	MSH4 MLH2
GO:0042493	response to drug	4	0.39%	8.70%	0.226406617022557	PDR16 CIN5
					0.	

GO:0006457	protein folding	6	1.57%	8.51%	232097473508520	HSP26 SSA4 BTN2 CPR6 TSA1 SIS1 APJ1 CUR1
GO:0044255	cellular lipid metabolic process	4	0.20%	7.69%	0.238753411057540	EHT1
GO:00068[illegible]	nitrogen compound metab[illegible]	3	0.20%	7.69%	0.238753411057[illegible]	GLT1

7	bolic process				540	
GO:0006415	translational termination	7	0.20%	7.69%	0.23875341105754 0	SSB1
GO:0032313	regulation of Rab GTPase activity	8	0.20%	7.69%	0.23875341105754 0	OCA5
GO:0	sterol tr		0.	7.	0.23875	

015918	transport	5	20%	69%	341105754 0	DAN1
GO:0030011	maintenance of cell polarity	5	0.20%	7.69%	0.238753411057540	RHO4
GO:0006873	cellular ion homeostasis	6	0.20%	7.69%	0.238753411057540	HRK1
GO:0	mitochondrio		0.	8.	0.2410627	

0070005	n organization	5	59%	57%	758607481	NCA3 RPM2 POR1
GO:0006896	Golgi to vacuole transport	5	0.39%	8.33%	0.246027241106585	GGA1 PEP12
GO:0000002	mitochondrial genome maintenance	6	0.59%	8.33%	0.257130792895950	HSP78 THI4 ILV5
	ax				0.	

GO:0007120	lar cellular bud site selection	5	0.20%	7.14%	266198028242768	GIC2
GO:0007266	Rho protein signal transduction	8	0.20%	7.14%	0.266198028242768	GIC2
GO:0006895	Golgi to endosome trans	5	0.20%	7.14%	0.2661980282427	GGA1

	port				768	
GO:0000028	ribosomal small subunit assembly	6	0.20%	7.14%	0.266198028242768	NSR1
GO:0016070	RNA metabolic process	5	0.20%	7.14%	0.266198028242768	RTC3
GO:0	fungal-type ce		0.	7.	0.266198	

[illegible]0092272	[illegible]ll wall biogenesis	5	[illegible]20%	[illegible]14%	[illegible]028242768	RPI1
GO:0046854	phosphatidylinositol phosphorylation	7	0.20%	7.14%	0.266198028242768	LSB6
GO:0000413	protein peptidyl-prolyl isom[illegible]	9	0.20%	7.14%	0.266198028242[illegible]	CPR6

	erization				768	
[GO:0030473]	nuclear migration along microtubule	6	0.20%	7.14%	0.266198028242768	[KIP2]
[GO:0000910]	cytokinesis	5	0.59%	8.11%	0.273403958065894	[HOF1] [EGT2] [ASE1]
[GO					0.2736	

:0006508	proteolysis	5	2.55%	8.02%	97566461874	RRT12 DOA4 ARX1 YGL258W-A YPS5 AAP1 BAR1 YIL082W-A APE1 RIM11 SNO4 HSP33 HSP32
GO:0006096	glycolysis	5	0.39%	7.69%	0.285948724269469	HXK1 YLR446W
GO:0006396	RNA processing	6	0.39%	7.69%	0.285948724269469	RPM2 RCL1
	intrace				0.28	

[GO:0006886]()	llular protein transport	5	1.37%	8.05%	6109612192771	[YET3]() [GGA1]() [SXM1]() [BTN2]() [PIR1]() [PEP12]() [CUR1]()
[GO:0007154]()	cell communication	4	0.20%	6.67%	0.293634899275811	[ATG20]()
	ubiquitin-dependent protein ca				0.	

[GO:0043162]()	catabolic process via the multivesicular body sorting pathway	9	0.20%	6.67%	2936348992758811	[DOA4]()
[GO:0016485]()	protein processing	6	0.20%	6.67%	0.2936348992758811	[RIM8]()

GO:0006098	pentose-phosphate shunt	6	0.20%	6.67%	0.293634899275811	SOL4
GO:0030174	regulation of DNA-dependent DNA replication initiation	9	0.20%	6.67%	0.293634899275811	CDC6
	n[illegible]					

GO:0000956	uclear-transcribed mRNA catabolic process	8	0.20%	6.67%	0.293634899275811	TIS11
GO:0006730	one-carbon metabolic process	4	0.20%	6.67%	0.293634899275811	SAM1
GO:[illegible]	cellular m[illegible]	[illegible]	[illegible]	[illegible]	0.29363[illegible]	

0044237	etabolic process	3	0.20%	6.67%	4899275811	GRE2
GO:0000079	regulation of cyclin-dependent protein serine/threonine kinase activ	6	0.39%	7.41%	0.306113609533597	PCL9 PCL7

	...ity					
GO:0051301	cell division	4	2.94%	7.77%	0.311228290329162	AME1 PCL9 SPR28 NDT80 PCL7 POG1 FAR1 CDC6 BFA1 SDS22 HOF1 EGT2 SPO21 MPC54 KIP2
GO:0045047	protein targeting to ER	5	0.20%	6.25%	0.320923784362665	WSC4
GO:00353...	peptidyl-tyrosine deph...	8	0.20%	6.25%	0.3209237843...	TEP1

[illegible]35	[illegible]osphorylation	[illegible]	[illegible]	[illegible]	[illegible]62665	
GO:0015914	phospholipid transport	5	0.20%	6.25%	0.320923784362665	PDR16
GO:0016311	dephosphorylation	6	0.39%	6.90%	0.346541893915368	PHO5 TEP1
GO:[illegible]	mitochondr[illegible]	[illegible]	[illegible]	[illegible]	0.3465[illegible]	[illegible]

[illegible]:0000422	[illegible]ion degradation	6	0.39%	6.90%	[illegible]1893915368	ATG20 ICY2
GO:0006754	ATP biosynthetic process	9	0.20%	5.88%	0.347944557309195	ATP8
GO:0015986	ATP synthesis coupled proton trans	7	0.20%	5.88%	0.347944557309195	ATP8

	port					
GO:0001300	chronological cell aging	5	0.20%	5.88%	0.347944557309195	GIS1
GO:0009408	response to heat	4	0.20%	5.88%	0.347944557309195	WSC4
GO:00355[illegible]	intracellular signal tr[illegible]	5	0.20%	5.88%	0.3479445573[illegible]	SSK1

[illegible]56	[illegible]ansduction				[illegible]09195	
GO:0006513	protein monoubiquitination	10	0.20%	5.88%	0.347944557309195	UBC11
GO:0030148	sphingolipid biosynthetic process	6	0.20%	5.88%	0.347944557309195	SUR1
	negative reg[illegible]					

GO:0000122	ulation of transcription from RNA polymerase II promoter	9	1.18%	7.50%	0.351127320092291	REG2 RPN4 GIS1 YGR122W DAT1 ROX1
GO:0015992	proton transport	5	0.59%	7.14%	0.356556786575399	ATP8 YDL183C YHK8

GO:0007124	pseudohyphal growth	4	0.78%	7.27%	0.360979557477602	NRG2 PHD1 NCS2 HMS1
GO:0007264	small GTPase mediated signal transduction	6	0.78%	7.27%	0.360979557477602	YCL068C YHR022C RHO4 BAG7
GO:	reg[illegible]				0.3666[illegible]	

:0008361	ulation of cell size	5	0.39%	6.67%	93787527784	PHO5 MSA1
GO:0016579	protein deubiquitination	9	0.20%	5.56%	0.374595048768485	DOA4
GO:0006913	nucleocytoplasmic transport	6	0.20%	5.56%	0.374595048768485	SXM1
	mitoch					

GO:0033617	ondrial respiratory chain complex IV assembly	6	0.20%	5.56%	0.374595048768485	PET117
GO:0006164	purine nucleotide biosynthetic process	7	0.20%	5.56%	0.374595048768485	ADE17
	cellula				0.	

GO:0044262	r carbohydrate metabolic process	4	0.20%	5.56%	374595048768485	GLO4
GO:0007034	vacuolar transport	4	0.20%	5.56%	0.374595048768485	MRL1
GO:000908[illegible]	methionine biosynthetic c[illegible]	7	0.39%	6.45%	0.38673675748[illegible]	STR3 MET2

6	process				8122	
GO:0042147	retrograde transport, endosome to Golgi	5	0.20%	5.26%	0.400789094612637	BTN2
GO:0000077	DNA damage checkpoint	6	0.20%	5.26%	0.400789094612637	TSA1
					0.4	

GO:0030488	tRNA methylation	6	0.20%	5.26%	.00789094612637	PPM2
GO:0008152	metabolic process	2	4.51%	7.28%	0.418148766958007	GLT1 GAL3 YDR018C TPS2 ARG5,6 SER3 AGX1 GSY1 HXK1 PNC1 POX1 YHL012W YIL082W-A POT1ACO2 BAT2 OAR1 YLR345W ADE17 ALD3 IDH2 MCT1 MUM3
GO:0	nuclear-transcribed mRNA		0.	5.	0.426454	

0070481	catabolic process, non-stop decay	9	20%	00%	.771175415	YGR122W
GO:0006267	pre-replicative complex assembly	7	0.20%	5.00%	0.426454771175415	CDC6
	positive regulation of tra				0.4	

GO:0045944	anscription from RNA polymerase II promoter	9	2.16%	7.14%	428339112012127	THI2 RPN4 PHO2 GIS1 VHR1 RPI1 PHD1 RPM2 RGM1 YRR1 YOR338W
GO:0006406	mRNA export from nucleus	6	0.39%	5.88%	0.445777393261585	SXM1 MIP6
GO:	lipid				0.45153	

0006869	transport	4	0.20%	4.76%	2800668512	PDR16
GO:0006812	cation transport	5	0.39%	5.71%	0.464969390260408	MMT1 MMT2
GO:0006810	transport	3	11.18%	7.18%	0.466496084827782	ATP8 FLR1 DTR1 MAL31 GEX1 YET3 ATG20 YDL199C YCF1 GGA1 SXM1 FIT1 PIC2 HXT10 YFL054CPRM8 YGL114W RRT6 TPO2 FMP43 MAL11 MUP3 ARN1 ARN2 YHK8 HXT4 HXT5 ECM27 MCH2 MRS4PTR2 AQY2 YLL053C MMP1 ATR1 MMT1 YMR279C FET4 LST8 POR1 AQR1 PDR16 HXT14 MDJ2 ATO2HXT17 ATG34 HXT11 CRC1 TPO4 MFM1 GUP2 MMT2 DIP5 MRL1 NCE102 ARR3
GO	protein				0.475	

[illegible]:0000209	polyubiquitination	10	0.20%	4.55%	[illegible]975111501836	UBC11
GO:0006366	transcription from RNA polymerase II promoter	7	1.37%	6.60%	0.495941426792965	THI2 RDS1 SIP4 GSM1 RSF1 YRR1 GAL4
GO:004[illegible]	ribosomal large sub[illegible]	5	0.3[illegible]	5.4[illegible]	0.5024337[illegible]	RIX7 RRS1

[illegible]42273	[illegible]bunitbiogenesis		[illegible]9%	[illegible]1%	[illegible]94979402	RIX7 RRS1
GO:0001302	replicative cell aging	5	0.39%	5.13%	0.538509158206264	PNC1 TSA1
GO:0016236	macroautophagy	6	0.20%	4.00%	0.545148721286620	ATG20
G[illegible]	mitotic sp[illegible]				0.545[illegible]	

O:0007094	indle assembly checkpoint	11	0.20%	4.00%	148721286620	HHT2
GO:0006200	ATP catabolic process	8	0.39%	5.00%	0.555976394687648	YDR061W YCF1
GO:0006355	regulation of transcription, DNA-d	7	6.86%	6.89%	0.5616810661152	NRG2 THI2 SRD1 RDS1 RPN4 PHO2 GIS1 ARG5,6 DOT6 RRT5 RRT6 MGA1 NDT80 REC104 VHR1 XBP1POG1 SIP4 GSM1 PHD1 ASH1 MSA2 SSK1 RRN11 RSF1 RGM1 CIN5 HMS1 MSA1 YRR1 CUP9 GAL4CSR2 ROX1 CUR1

	ependent				30	
GO:0007031	peroxisome organization	5	0.20%	3.85%	0.566748721038050	GPX1
GO:0016126	sterol biosynthetic process	7	0.20%	3.70%	0.587599529958406	PDR16
GO:0	met		0.	5.	0.597524	

[illegible]032259	[illegible]hylation	3	[illegible]78%	[illegible]56%	[illegible]173900439	KAR4 PPM1 IME4 PPM2
GO:0051726	regulation of cell cycle	5	0.20%	3.57%	0.607697139150337	PCL9
GO:0006890	retrograde vesicle-mediated transp[illegible]	5	0.20%	3.57%	0.6076971391503[illegible]	TRX2

	ort, Golgi to ER				[illegible]7	
[GO:0006302]	double-strand break repair	7	0.20%	3.57%	0.607697139150337	[TPP1]
[GO:0006461]	protein complex assembly	5	0.20%	3.57%	0.607697139150337	[ATG34]
[G[illegible]]	ribosomal				0.627[illegible]	

O:0042274	small subunit biogenesis	5	0.20%	3.45%	041972130705	KRE33
GO:0042144	vacuole fusion, non-autophagic	6	0.20%	3.33%	0.645638271030914	TRX2
GO:0030010	establishment of cell polar	5	0.20%	3.23%	0.6634935478269	GIC2

	[illegible]ity				[illegible]69	
GO:0006487	protein N-linked glycosylation	8	0.20%	3.23%	0.663493547826969	OCH1
GO:0030036	actin cytoskeleton organization	5	0.20%	3.12%	0.680618094452264	SLM1
GO:00704[illegible]	cell cycle	4	3.92%	6.31%	0.68553812057[illegible]	AME1 PCL9 SPR28 VHS1 ZIP2 BNS1 NDT80 PCL7 POG1 FAR1 CDC6 BFA1 SDS22 HOF1 SIS1 EGT2SPO21 MSA1 MPC54 KIP2

9					72234	
GO:0000027	ribosomal large subunit assembly	6	0.20%	3.03%	0.697024546191050	RSA4
GO:0007067	mitosis	5	1.37%	5.34%	0.733794858568910	AME1 POG1 BFA1 SDS22 HOF1 EGT2 KIP2
GO:00	lipid metab		1.5	5.4	0.7386677	

[illegible]0066629	[illegible]bolic process	4	[illegible]7%	[illegible]4%	[illegible]771730714	FRM2 YDR018C OLE1 POX1 POT1 OAR1 OPI10 MCT1
GO:0009058	biosynthetic process	3	0.20%	2.78%	0.742088978657572	NMA2
GO:0043547	positive regulation of GTPase activity	7	0.20%	2.78%	0.742088978657572	BAG7
	chrom[illegible]				0.74[illegible]	

GO:0006348	[illegible]ation silencing at telomere	6	0.39%	3.77%	[illegible]4106093185516	DOT6 PNC1
GO:0042254	ribosome biogenesis	5	1.96%	5.59%	0.748590090550175	UTP20 RSA4 RTC3 IMP3 UTP10 RIX7 UTP13 NOG2 RCL1 RRS1
GO:0006470	protein dephosphorylation	7	0.20%	2.70%	0.755783560582038	TEP1

[GO:0032258]()	CVT pathway	6	0.20%	2.63%	0.768846216626254	[ATG20]()
[GO:0030433]()	ER-associated protein catabolic process	8	0.20%	2.38%	0.815181120279521	[HRD1]()
[GO:000]()	transcription, DNA-	6	6.27	6.08	0.82239874	[NRG2]() [THI2]() [RDS1]() [RPN4]() [PHO2]() [GIS1]() [DOT6]() [RRT5]() [RRT6]() [NDT80]() [REC104]() [VHR1]() [XBP1]() [POG1]() [SIP4]() [GSM1UTP10]() [RPA12]() [PHD1]() ASH1 MSA2 RPN11 RSF1 PGM1 CIN5 HMS1 MSA1 YRR1 GAL4

6351	dependent		%	%	6779803	ASH1 MSA2 RRN11 RSF1 RGM1 CIN5 HMS1 MSA1 YRR1 GAL4 CSR2 ROX1 CUR1
GO:0046797	viral procapsid maturation	4	0.20%	2.27%	0.835087128141993	YIL082W-A
GO:0[illegible]	maturation of SSU-rRNA from tricistronic rR[illegible]		[illegible]	[illegible]	0.840766[illegible]	

0000462	rRNA transcript (SSU-rRNA, 5.8S rRNA, LSU-rRNA)	7	0.39%	3.17%	6623967690	UTP10 UTP13
GO:0006357	regulation of transcription from RNA p	8	0.98%	4.35%	0.84360723339 1	RDS1 DOT6 GSM1 CIN5 CSR2

-	olymerase II promoter				866	
GO:0015074	DNA integration	6	0.20%	2.17%	0.853037115656471	YIL082W-A
GO:0006511	ubiquitin-dependent protein cata	8	0.39%	3.03%	0.86282995195 76	DOA4 ASI3

	...tabolic process				...17	
GO:0008033	tRNA processing	8	0.59%	3.57%	0.8630957808578895	RPM2 NCS2 PPM2
GO:0006413	translational initiation	4	0.20%	2.08%	0.8691898537816566	SIS1
GO:0...	regulatio...		0...	2...	0.876640...	

[illegible]006417	[illegible]n of translation	7	0.20%	2.04%	[illegible]166985537	FES1
GO:0007059	chromosome segregation	4	0.39%	2.90%	0.882164225008510	ZIP2 SDS22
GO:0006897	endocytosis	5	0.59%	3.41%	0.885936658441565	SDS24 DOA4 SUR7
	nucleic ac[illegible]					

GO:0090305	acid phosphodiester bond hydrolysis	6	0.59%	3.41%	0.885936658441565	YIL082W-A RPM2 YMR262W
GO:0006914	autophagy	5	0.20%	1.96%	0.890380427238390	ATG34
GO:0006[illegible]	chromatin remo[illegible]	8	0.20%	1.92%	0.89670553 2[illegible]	PHO2

338	deling				1908809	
GO:0006364	rRNA processing	6	1.76%	4.55%	0.912130862613076	UTP20 SRD1 SSB1 NSR1 RTC3 IMP3 UTP10 UTP13 SSB2
GO:0016567	protein ubiquitination	9	0.39%	2.60%	0.922377466895012	HRD1 UBC11
GO:00	vesicle-mediat	4	0.9	3.5	0.943453...	

16192	ed transport	4	8%	7%	561103716	YET3 RCR2 GGA1 PRM8 PEP12
GO:0051028	mRNA transport	5	0.20%	1.59%	0.947074799379927	SXM1
GO:0000398	mRNA splicing, via spliceosome	8	0.39%	2.35%	0.949664476436481	SMD2 CWC25
GO	cytopl				0.96l2	

:0002181	asmic translation	7	1.18%	3.59%	15989899509	FES1 SSB1 RPL22b RPM2 SSB2 RPL7b
GO:0006281	DNA repair	6	1.37%	3.74%	0.964062202534125	HTA2 SAE2 MLH2 HUG1 TPP1 DDR48 HHT2
GO:0006260	DNA replication	6	0.20%	1.32%	0.976664908105209	CDC6
	respo				0.9	

GO:0006974	[illegible]nse to DNA damage stimulus	5	1.37%	3.47%	[illegible]98102184445888 3	HTA2 SAE2 MLH2 HUG1 HOF1 TPP1 HHT2
GO:0006888	ER to Golgi vesicle-mediated transport	5	0.20%	1.25%	0.981953048172494	TRX2
GO:00[illegible]	mitochondrial [illegible]	6	0.3[illegible]	1.8[illegible]	0.98725494[illegible]	[illegible]

[illegible]32543	[illegible]translation	[illegible]	[illegible]9%	[illegible]3%	[illegible]44586297	MRPL25 MRPL33
GO:0008380	RNA splicing	7	0.39%	1.82%	0.987987998899714	SMD2 CWC25
GO:0016568	chromatin modification	7	0.39%	1.72%	0.991605182239137	SET4 ASH1
GO:0[illegible]	DNA re[illegible]	[illegible]	0[illegible]	1[illegible]	0.992274[illegible]	[illegible]

0006310	combination	6	0.20%	1.08%	4689809783	YIL082W-A
GO:0032197	transposition, RNA-mediated	5	0.20%	1.05%	0.993230945396243	YIL082W-A
GO:0006397	mRNA processing	7	0.78%	2.33%	0.995618700584673	MRS4 SMD2 RPM2 CWC25
G[illegible]	pr[illegible]				0.990[illegible]	

GO:0015031	protein transport	4	2.16%	2.82%	[illegible]9890973786306	YET3 ATG20 GGA1 SXM1 YGL114W TRX2 PTR2 MDJ2 ATG34 MRL1 NCE102
GO:0006412	translation	6	0.78%	1.45%	0.999990277112938	SSB1 RPL22b MRPL33 SSB2
